# The effectiveness of interventions that support penicillin allergy assessment and delabeling of adult and pediatric patients by nonallergy specialists: a systematic review and meta-analysis

**DOI:** 10.1016/j.ijid.2022.11.026

**Published:** 2023-04

**Authors:** Neil Powell, Jennie Stephens, Declan Kohl, Rhys Owens, Shadia Ahmed, Crispin Musicha, Mathew Upton, Bridie Kent, Sarah Tonkin-Crine, Jonathan Sandoe

**Affiliations:** 1 Pharmacy Department, Royal Cornwall Hospital, Truro, United Kingdom TR1 3LJ / School of Biomedical Sciences, University of Plymouth, Plymouth, United Kingdom; 2 Intensive Care Department, Royal Cornwall Hospital, Truro, United Kingdom; 3 School of Biomedical Sciences, University of Leeds, Leeds, United Kingdom; 4 Core Medical Trainee, Royal Cornwall Hospital, Truro, United Kingdom; 5 Leeds Institute of Medical Research, University of Leeds, Leeds, United Kingdom; 6 Medical Statistics, Faculty of Health, University of Plymouth, Plymouth, United Kingdom; 7 School of Biomedical Sciences, University of Plymouth, Plymouth, United Kingdom; 8 School of Nursing and Midwifery, University of Plymouth, Plymouth, United Kingdom; 9 Nuffield Department of Primary Care Health Sciences, University of Oxford, Oxford, United Kingdom

**Keywords:** Antimicrobial stewardship, Penicillin allergy assessment, Penicillin allergy delabeling, Nonallergists

## Abstract

**Objectives:**

Penicillin allergy records are often incorrect and may result in harm. We aimed to systematically review the effectiveness and safety of nonallergist health care worker delivery of penicillin allergy delabeling.

**Methods:**

We searched EMBASE/MEDLINE/CINAHL (Ovid), PsycInfo, Web of Science, and Cochrane CENTRAL from inception to January 21, 2022 and unpublished studies and gray literature. The proportion of patients allergic to penicillin delabeled and harmed was calculated using random-effects models.

**Results:**

Overall, 5019 patients were delabeled. Using allergy history alone, 14% (95% confidence interval [CI], 9-21%) of 4350 assessed patients were delabeled without reported harm. Direct drug provocation testing resulted in delabeling in 27% (95% CI, 18-37%) of 4207 assessed patients. Of the 1373 patients tested, 98% were delabeled (95% CI, 97-99%), and nonserious harm was reported in 1% (95% CI, 0-2%). Using skin testing, followed by drug provocation testing, 41% (95% CI, 24-59%) of 2890 assessed patients were delabeled. Of the 1294 tested patients, 95.0% (95% CI, 90-99%) were delabeled, and the reported harm was low (0%; (95% CI 0-1%).

**Conclusion:**

Penicillin allergy delabeling by nonallergists is efficacious and safe. The proportion of assessed patients who can be delabeled increases with the complexity of testing method, but substantial numbers can be delabeled without skin testing.

## Highlights

• Penicillin allergy delabeling by nonallergists is safe.
• Less intensive methods delabeled a smaller proportion of patients.
• Once patients were assessed as suitable for testing, rates of delabeling were high.
• A diverse workforce engaged in delabeling incorrect penicillin allergy records.
• Penicillin allergy delabeling interventions are described.

## Introduction

Approximately 6% of the general population (West *et al.*, 2019) and 15% of hospital inpatients have a record of penicillin allergy (penA; Macy and Contreras, 2014; Powell *et al.*, 2020; Trubiano *et al.*, 2018). Penicillin-based antibiotics are first-line treatment for many infections, but patients with penA labels are usually treated with second-line antibiotics (Powell *et al.*, 2020), which are often more costly, can be less effective in certain clinical circumstances, more toxic, and often have broader spectrum, potentially increasing a patient's risk of future infections with resistant bacteria (Krah *et al.*, 2021). More than 95% of individuals with a penA label can tolerate penicillin (DesBiens *et al.*, 2020; Shenoy *et al.*, 2019).

The assessment of patients with reported penAs has been the role of allergists, but allergy services are limited (Krishna *et al.*, 2017). Traditional penA testing requires skin testing (ST) before drug provocation testing, which remains the main testing method in Europe, making penA testing resource intense (Mirakian *et al.*, 2015; Romano *et al.*, 2020). Direct drug provocation testing (DPT), an oral challenge test, in patients with a low-risk allergy history is less resource intense. Two systematic reviews have confirmed the safety and efficacy of DPT (without previous ST) as a method of delabeling adults, delivered both by allergists and nonallergists (Cooper *et al.*, 2021; DesBiens *et al.*, 2020). ST before DPT has also been successfully delivered by nonallergists (Englert and Weeks, 2019; Wall *et al.*, 2004).

The American Academy of Allergy Asthma and Immunology (2020) with the Infectious Diseases Society of America wrote to the Centers for Medicare and Medicaid Services to urge US hospitals to include verification of penA as part of its mandatory antibiotic stewardship programs. The World Health Organization (2021) has since recommended antibiotic delabeling as an effective antimicrobial stewardship strategy. The enablement of the wider health care workforce to delabel eligible patients is required to deliver penA assessment and delabeling at a large scale. Understanding the wider frameworks that enable nonallergists to safely delabel is required, enabling the development of effective interventions that facilitate penA delabeling by nonallergy specialists.

We systematically reviewed the literature to determine the proportion of patients with a reported penA who were safely delabeled by nonallergy health care workers (HCWs), categorizing the components of interventions using the Effective Practice and Organisation of Care (EPOC) taxonomy of health interventions (Effective Practice and Organisation of Care [EPOC], 2016) and report any measured antimicrobial stewardship and health system impact.

## Methods

This systematic review and meta-analysis were conducted in accordance with the Joanna Briggs Institute methodology for systematic reviews of effectiveness (Tufanaru *et al.*, 2020) and is reported using the Preferred Reporting Items for Systematic Reviews and Meta-Analysis checklist (Liberati *et al.*, 2009).

### Inclusion/exclusion criteria

The inclusion criteria are as follows: (i) any patient (adult/child) with a penA record in any health care context, (ii) having undergone penA delabeling (PADL) using any method; and (iii) by nonallergy specialists, defined as a medical professional whose primary specialization is not in allergy or who has not trained in allergy as part of their specialty (Savic, Khan et al. 2019). The penA assessment and delabeling interventions delivered by immunologists or allergy specialists were excluded. All study designs were included, except case reports.

### Search strategy

The following databases were searched from inception to January 21, 2022 (NP) EMBASE (Ovid), MEDLINE (Ovid), CINAHL (Ovid), PsycInfo, Web of Science, and Cochrane CENTRAL, as was the gray literature. Known experts in the topic were contacted to ensure we have not overlooked relevant literature. The search strategy was reviewed by an experienced information specialist (KO). Only studies published in English were included due to a lack of funding for translation services (Appendix 1).

Titles and abstracts were screened by two independent reviewers (NP, SA, DK, RO, JS) against the inclusion criteria (RAYYAN software; Ouzzani *et al.*, 2016). Full-text citations were assessed against the inclusion criteria by two independent reviewers (NP, RO) using RAYYAN software (Ouzzani *et al.*, 2016; Appendix 2 and 3). Disagreements were resolved through discussion.

### Assessment of methodological quality

Eligible studies were critically appraised by two reviewers (NP, BK) using critical appraisal instruments from the Joanna Briggs Institute (Tufanaru *et al.*, 2020). Authors were contacted to request additional data, where required. Studies were not excluded on the grounds of their risk of bias.

### Data extraction

Data were extracted by one reviewer (NP), using a purpose-built extraction tool in Excel (Microsoft Corporation, 2018) and included the study design, country, setting, population age, gender, inclusion criteria, exclusion criteria, allergy testing method(s), HCW(s) delivering PADL, components of the PADL interventions, details about education and training, number of assessed patients, number tested, number that experienced unintended harm, and any reported antibiotic stewardship or health care system impact. The extraction of data from seven (10%) studies was validated by a second reviewer. Intervention components were categorized using the EPOC taxonomy of health interventions, enabling the grouping of health system interventions by conceptual or practical similarities (EPOC, 2016). Studies that used a risk stratification protocol for allergy testing were categorized in the “packages of care” subcategory. Complex interventions were categorized into the “care pathways” subcategory (Skivington *et al.*, 2021). Governance arrangements were categorized as “authority and accountability for quality of practice”.

### Definitions

See Appendix 4 for definitions for delabeling, ST/DPT, direct DPT (DDPT) and direct delabeling on history alone (DDL), successful delabel, and definitions of harm.

### Data analysis

The population-weighted proportional meta-analysis was conducted on studies with a low/moderate risk of bias to determine the proportion of participants successfully delabeled and the proportion with a positive penA test by the delabel method (DDL, DDPT, and ST/DPT) using the R package meta v 5.2.0 (Schwarzer, 2022). Statistical heterogeneity was assessed using the chi-square test (threshold *P* <0.1) and the I^2^ statistic (I^2^ values <25%, 25-75%, and >75% were considered to represent low, moderate, and high heterogeneity, respectively). Overall estimates were obtained using random-effects models (Tufanaru *et al.*, 2015). A funnel plot was generated to assess publication bias, with funnel plot asymmetry tested using the Egger test (Egger *et al.*, 1997). We used the studentized residual to identify studies that contributed most to heterogeneity (Viechtbauer and Cheung, 2010). Studies with z absolute values >1.96 (Viechtbauer and Cheung, 2010) were excluded from the analysis to assess their influence on the overall estimates. The remaining data are presented in narrative form.

## Results

### Study inclusion

**Figure 1:**
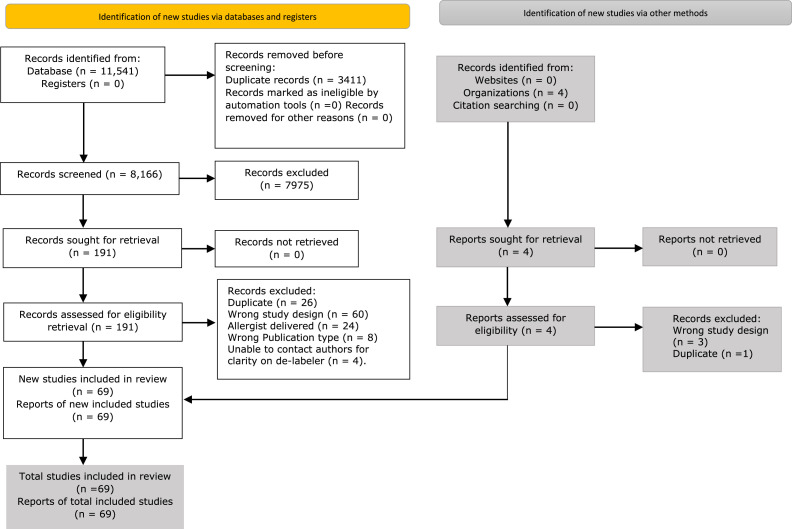
Flow diagram illustrating included and excluded studies.

In total, 11,545 studies were identified, of which 3411 were excluded due to duplication. The review of titles and abstracts by two authors (DK, NP, SA, RO, JS) led to the retrieval of 191 full papers for screening by two authors (NP, RO, JS, MU, STC); 69 were included in the systematic review (Figure 1). A total of 56 studies were case series (Adkinson *et al.*, 1971; Allen *et al.*, 2020; Bauer *et al.*, 2021; Blackwell and Khan, 2020; Blumenthal *et al.*, 2019; Chen *et al.*, 2017; Devchand *et al.*, 2019; du Plessis *et al.*, 2019; Englert and Weeks, 2019; Eischens *et al.*, 2018; Foolad *et al.*, 2019; Gugkaeva *et al.*, 2017; Griffith *et al.*, 2020; Harmon *et al.*, 2020; Ham *et al.*, 2021; Harris *et al.*, 1999; Harper and Sanchez, 2022; Heil *et al.*, 2016; Jones and Bland, 2017; Jones et al., 2019b; Kleris *et al.*, 2018; Kyi *et al.*, 2018; Lecerf *et al.*, 2020; Leis *et al.*, 2017; Lin *et al.*, 2020; Livirya *et al.*, 2022; Lnumerables and Fischer-Cartlidge, 2020; Lo *et al.*, 2020; Louden *et al.*, 2021; Maguire *et al.*, 2020; Marwood *et al.*, 2017; Mitchell *et al.*, 2021; Morjaria *et al.*, 2021; Murphy *et al.*, 2015; Parker *et al.*, 2018; Patel *et al.*, 2019; Phung *et al.*, 2021; Rahbani, 2019; Rahbani and Monroe-Duprey, 2020; Rimawi *et al.*, 2013; Rimawi and Mazer, 2014; Savic *et al.*, 2019; Sigona *et al.*, 2016; Skibba *et al.*, 2014; Smibert *et al.*, 2018; Sneddon *et al.*, 2021; Song *et al.*, 2021; Steenvoorden *et al.*, 2021; Stone *et al.*, 2020; Taremi *et al.*, 2019; Torney and Tiberg, 2018, 2021; Trubiano *et al.*, 2018; Wall *et al.*, 2004; Wong *et al.*, 2018; Wrenn *et al.*, 2017), ten were quasi-experimental studies (Blumenthal *et al.*, 2015; Chen *et al.*, 2018; Gaudreau *et al.*, 2021; Jones *et al.*, 2019a; Nguyen *et al.*, 2019; Ravindran *et al.*, 2017; Shannon and Krop, 2016; Stein *et al.*, 2020; Trubiano *et al.*, 2017; Sacco *et al.*, 2019), two were cohort studies (Chua *et al.*, 2021; Trubiano *et al.*, 2022), and one was a randomized controlled trial (Vyles *et al.*, 2020).

### Methodological quality

Of the 56 case series studies, six, 19, and 31 had a high, moderate, and low risk of bias, respectively. The risk of bias assessments are shown in Appendix 5.

### Characteristics of included studies

The 69 included studies reported on the successful PADL of 5019 patients (adults n = 4314 [Adkinson *et al.*, 1971; Blumenthal *et al.*, 2015, 2019; Blackwell and Khan, 2020; Chen *et al.*, 2017, 2018; Chua *et al.*, 2021; Devchand *et al.*, 2019; du Plessis *et al.*, 2019; Englert and Weeks, 2019; Eischens *et al.*, 2018; Foolad *et al.*, 2019; Gugkaeva *et al.*, 2017; Griffith *et al.*, 2020; Gaudreau *et al.*, 2021; Ham *et al.*, 2021; Harmon *et al.*, 2020; Harper and Sanchez, 2022; Harris *et al.*, 1999; Heil *et al.*, 2016; Jones and Bland, 2017; Jones *et al.*, 2019a; Jones et al., 2019b; Kyi *et al.*, 2018; Leis *et al.*, 2017; Lin *et al.*, 2020; Livirya *et al.*, 2022; Lnumerables and Fischer-Cartlidge, 2020; Lo *et al.*, 2020; Maguire *et al.*, 2020; Marwood *et al.*, 2017; Mitchell *et al.*, 2021; Morjaria *et al.*, 2021; Nguyen *et al.*, 2019; Patel *et al.*, 2019; Parker *et al.*, 2018; Phung *et al.*, 2021; Rimawi and Mazer, 2014; Sacco *et al.*, 2019; Savic *et al.*, 2019; Shannon and Krop, 2016; Sigona *et al.*, 2016; Skibba *et al.*, 2014; Smibert *et al.*, 2018; Sneddon *et al.*, 2021; Song *et al.*, 2021; Steenvoorden *et al.*, 2021; Stone *et al.*, 2020; Taremi *et al.*, 2019; Torney and Tiberg, 2018, 2021; Trubiano *et al.*, 2017, 2018, 2022; Wall *et al.*, 2004; Wrenn *et al.*, 2017]; children n = 461 [Allen *et al.*, 2020; Bauer *et al.*, 2021; Lecerf *et al.*, 2020; Louden *et al.*, 2021; Murphy *et al.*, 2015; Rahbani and Monroe-Duprey, 2020; Stein *et al.*, 2020; Wong *et al.*, 2018; Vyles *et al.*, 2020]; unreported n = 244 [Kleris *et al.*, 2018; Rahbani, 2019; Ravindran *et al.*, 2017]). The studies were from the United States (n = 48; Adkinson *et al.*, 1971; Bauer *et al.*, 2021; Blackwell and Khan, 2020; Blumenthal *et al.*, 2015, 2019; Chen *et al.*, 2017, 2018; Eischens *et al.*, 2018; Englert and Weeks, 2019; Foolad *et al.*, 2019; Gugkaeva *et al.*, 2017; Griffith *et al.*, 2020; Ham *et al.*, 2021; Harmon *et al.*, 2020; Harper and Sanchez, 2022; Harris *et al.*, 1999; Heil *et al.*, 2016; Jones and Bland, 2017; Jones *et al.*, 2019a; Jones et al., 2019b; Kleris *et al.*, 2018; Lecerf *et al.*, 2020; Lnumerables and Fischer-Cartlidge, 2020; Louden *et al.*, 2021; Maguire *et al.*, 2020; Mitchell *et al.*, 2021; Morjaria *et al.*, 2021; Nguyen *et al.*, 2019; Patel *et al.*, 2019; Parker *et al.*, 2018; Rahbani, 2019; Rahbani and Monroe-Duprey, 2020; Ravindran *et al.*, 2017; Rimawi *et al.*, 2013; Rimawi and Mazer, 2014; Sacco *et al.*, 2019; Shannon and Krop, 2016; Sigona *et al.*, 2016; Skibba *et al.*, 2014; Song *et al.*, 2021; Stein *et al.*, 2020; Stone *et al.*, 2020; Taremi *et al.*, 2019; Torney and Tiberg, 2018, 2021; Wall *et al.*, 2004; Wrenn *et al.*, 2017; Vyles *et al.*, 2020), Australia (n = 9; Chua *et al.*, 2021; Devchand *et al.*, 2019; Kyi *et al.*, 2018; Marwood *et al.*, 2017; Phung *et al.*, 2021; Smibert *et al.*, 2018; Trubiano *et al.*, 2017, 2018, 2022), Canada (n = 4; Gaudreau *et al.*, 2021; Leis *et al.*, 2017; Lo *et al.*, 2020; Wong *et al.*, 2018), Ireland (n = 2; Allen *et al.*, 2020; Murphy *et al.*, 2015), New Zealand (n = 2; du Plessis *et al.*, 2019; Livirya *et al.*, 2022), the UK (n = 2; Savic *et al.*, 2019; Sneddon *et al.*, 2021), the Netherlands (n = 1; Lin *et al.*, 2020), and Norway (n = 1; Savic *et al.*, 2019); most were inpatient studies (n = 56; 81.2%; Adkinson *et al.*, 1971; Bauer *et al.*, 2021; Blackwell and Khan, 2020; Blumenthal *et al.*, 2015, 2019; Chen *et al.*, 2017, 2018; Chua *et al.*, 2021; Devchand *et al.*, 2019; du Plessis *et al.*, 2019; Englert and Weeks, 2019; Foolad *et al.*, 2019; Gaudreau *et al.*, 2021; Gugkaeva *et al.*, 2017; Griffith *et al.*, 2020; Ham *et al.*, 2021; Harmon *et al.*, 2020; Harper and Sanchez, 2022; Harris *et al.*, 1999; Heil *et al.*, 2016; Jones and Bland, 2017; Jones *et al.*, 2019a; Jones et al., 2019b; Kleris *et al.*, 2018; Kyi *et al.*, 2018; Lecerf *et al.*, 2020; Leis *et al.*, 2017; Lin *et al.*, 2020; Livirya *et al.*, 2022; Louden *et al.*, 2021; Mitchell *et al.*, 2021; Nguyen *et al.*, 2019; Patel *et al.*, 2019; Parker *et al.*, 2018; Phung *et al.*, 2021; Ravindran *et al.*, 2017; Rahbani, 2019; Rimawi *et al.*, 2013; Rimawi and Mazer, 2014; Sacco *et al.*, 2019; Shannon and Krop, 2016; Sigona *et al.*, 2016; Skibba *et al.*, 2014; Smibert *et al.*, 2018; Song *et al.*, 2021; Steenvoorden *et al.*, 2021; Stein *et al.*, 2020; Stone *et al.*, 2020; Taremi *et al.*, 2019; Torney and Tiberg, 2018, 2021; Trubiano *et al.*, 2017, 2022; Wall *et al.*, 2004; Wrenn *et al.*, 2017; Wong *et al.*, 2018), four in the emergency department only (Eischens *et al.*, 2018; Marwood *et al.*, 2017; Maguire *et al.*, 2020; Vyles *et al.*, 2020), four in the outpatient setting (Allen *et al.*, 2020; Lo *et al.*, 2020; Rahbani and Monroe-Duprey, 2020; Morjaria *et al.*, 2021), three conducted in both the inpatient and the outpatient setting (Lnumerables and Fischer-Cartlidge, 2020; Murphy *et al.*, 2015, Trubiano *et al.*, 2018), one inpatient and peri-op (Sneddon *et al.*, 2021), and one perioperation only (Savic *et al.*, 2019). The clinical settings included general/internal medicine (n = 23; Adkinson *et al.*, 1971; Blumenthal *et al.*, 2015, 2019; Chen *et al.*, 2017, 2018; Chua *et al.*, 2021; Englert and Weeks, 2019; Harmon *et al.*, 2020; Heil *et al.*, 2016; Kyi *et al.*, 2018; Leis *et al.*, 2017; Livirya *et al.*, 2022; Louden *et al.*, 2021; Mitchell *et al.*, 2021; Nguyen *et al.*, 2019; Parker *et al.*, 2018; Rimawi *et al.*, 2013; Sacco *et al.*, 2019; Sneddon *et al.*, 2021; Song *et al.*, 2021; Steenvoorden *et al.*, 2021; Torney and Tiberg, 2021; Trubiano *et al.*, 2022), intensive care (n = 12; Blumenthal *et al.*, 2019; Chen *et al.*, 2018; Heil *et al.*, 2016; Jones *et al.*, 2019a; Leis *et al.*, 2017; Louden *et al.*, 2021; Phung *et al.*, 2021; Rimawi *et al.*, 2013; Rimawi and Mazer, 2014; Stone *et al.*, 2020; Torney and Tiberg, 2021; Trubiano *et al.*, 2022), surgery/general surgery (n = 10; Blumenthal *et al.*, 2015, 2019; Chen *et al.*, 2017, 2018; Chua *et al.*, 2021; Heil *et al.*, 2016; Jones *et al.*, 2019a; Rimawi *et al.*, 2013; Song *et al.*, 2021; Trubiano *et al.*, 2022), oncology (n = 11; Blumenthal *et al.*, 2015, 2019; Chua *et al.*, 2021; Foolad *et al.*, 2019; Jones *et al.*, 2019a; Morjaria *et al.*, 2021; Smibert *et al.*, 2018; Taremi *et al.*, 2019; Trubiano *et al.*, 2017, 2018, 2022), hematology (n = 9; Foolad *et al.*, 2019; Lnumerables and Fischer-Cartlidge, 2020; Lo *et al.*, 2020; Morjaria *et al.*, 2021; Smibert *et al.*, 2018, Taremi *et al.*, 2019, Trubiano *et al.*, 2017, 2018, 2022), emergency department (n = 8; Blumenthal *et al.*, 2019; Eischens *et al.*, 2018; Jones *et al.*, 2019a; Maguire *et al.*, 2020; Marwood *et al.*, 2017; Murphy *et al.*, 2015; Rimawi *et al.*, 2013; Vyles *et al.*, 2020), pediatrics (n = 6; Allen *et al.*, 2020; Bauer *et al.*, 2021; Blumenthal *et al.*, 2019; Lecerf *et al.*, 2020; Stein *et al.*, 2020; Wong *et al.*, 2018), obstetrics and gynecology (n = 5; Blumenthal *et al.*, 2019; Chen *et al.*, 2017; Jones *et al.*, 2019a; Rimawi *et al.*, 2013; Song *et al.*, 2021), perioperative (n = 4; Harris *et al.*, 1999; Rahbani and Monroe-Duprey, 2020; Savic *et al.*, 2019; Sneddon *et al.*, 2021), transplant services (n = 3; Lnumerables and Fischer-Cartlidge, 2020; Lo *et al.*, 2020; Trubiano *et al.*, 2017), infectious diseases (n = 4; Jones et al., 2019b; Sneddon *et al.*, 2021; Torney and Tiberg, 2018; Trubiano *et al.*, 2017), cardiology (n = 2; Blumenthal *et al.*, 2015, 2019), urology (n = 1; Blumenthal *et al.*, 2015), oral maxillofacial surgery (n = 1; Blumenthal *et al.*, 2015), and neurology (n = 1).(Blumenthal *et al.*, 2019). Most studies attempted to delabel those patients with a low-risk allergy history only (n = 26; Allen *et al.*, 2020; Bauer *et al.*, 2021; Blumenthal *et al.*, 2015, 2019; Chua *et al.*, 2021; Devchand *et al.*, 2019; du Plessis *et al.*, 2019; Kyi *et al.*, 2018; Lecerf *et al.*, 2020; Lin *et al.*, 2020; Livirya *et al.*, 2022; Louden *et al.*, 2021; Mitchell *et al.*, 2021; Maguire *et al.*, 2020; Nguyen *et al.*, 2019; Phung *et al.*, 2021; Sacco *et al.*, 2019; Savic *et al.*, 2019; Sigona *et al.*, 2016; Smibert *et al.*, 2018; Sneddon *et al.*, 2021; Song *et al.*, 2021; Steenvoorden *et al.*, 2021; Stein *et al.*, 2020; Trubiano *et al.*, 2018, 2022) and moderate-risk allergy history only (n = 21; Chen *et al.*, 2017; Englert and Weeks, 2019; Foolad *et al.*, 2019; Gugkaeva *et al.*, 2017; Harmon *et al.*, 2020; Harper and Sanchez, 2022; Harris *et al.*, 1999; Heil *et al.*, 2016; Jones and Bland, 2017; Jones *et al.*, 2019a; Jones et al., 2019b; Leis *et al.*, 2017; Marwood *et al.*, 2017; Morjaria *et al.*, 2021; Rimawi *et al.*, 2013; Rimawi and Mazer, 2014; Shannon and Krop, 2016; Taremi *et al.*, 2019; Torney and Tiberg, 2018, 2021; Wall *et al.*, 2004), two studies included low- and moderate-risk history (Chen *et al.*, 2018; Gaudreau *et al.*, 2021), two studies included low-, moderate-, and high-risk allergy history (Ham *et al.*, 2021; Trubiano *et al.*, 2017); the risk category was unclear in 18 studies (Adkinson *et al.*, 1971; Blackwell and Khan, 2020; Eischens *et al.*, 2018; Griffith *et al.*, 2020; Kleris *et al.*, 2018; Lnumerables and Fischer-Cartlidge, 2020; Lo *et al.*, 2020; Murphy *et al.*, 2015; Parker *et al.*, 2018; Patel *et al.*, 2019; Rahbani, 2019; Rahbani and Monroe-Duprey, 2020; Ravindran *et al.*, 2017; Skibba *et al.*, 2014; Stone *et al.*, 2020; Wrenn *et al.*, 2017; Wong *et al.*, 2018; Vyles *et al.*, 2020; Appendix 6).

## Review findings

### Primary outcomes

#### Proportion of patients successfully delabeled and the proportion experiencing harm

In the studies with compete data on numbers of patients assessed for PADL (n = 47), 11,856 patients were assessed for testing, of whom 3720 (31.4%) were delabeled (Adkinson *et al.*, 1971; Allen *et al.*, 2020; Bauer *et al.*, 2021; Blackwell and Khan, 2020; Blumenthal *et al.*, 2019; Chen *et al.*, 2017, 2018; Chua *et al.*, 2021; Devchand *et al.*, 2019; du Plessis *et al.*, 2019; Englert and Weeks, 2019; Foolad *et al.*, 2019; Gaudreau *et al.*, 2021; Griffith *et al.*, 2020; Harmon *et al.*, 2020; Harper and Sanchez, 2022; Harris *et al.*, 1999; Heil *et al.*, 2016; Jones *et al.*, 2019a; Jones et al., 2019b; Kyi *et al.*, 2018; Lecerf *et al.*, 2020; Leis *et al.*, 2017; Lin *et al.*, 2020; Livirya *et al.*, 2022; Lo *et al.*, 2020; Louden *et al.*, 2021; Marwood *et al.*, 2017; Mitchell *et al.*, 2021; Murphy *et al.*, 2015; Nguyen *et al.*, 2019; Patel *et al.*, 2019; Phung *et al.*, 2021; Rimawi *et al.*, 2013; Rimawi and Mazer, 2014; Savic *et al.*, 2019; Shannon and Krop, 2016; Sigona *et al.*, 2016; Sneddon *et al.*, 2021; Song *et al.*, 2021; Steenvoorden *et al.*, 2021; Stone *et al.*, 2020; Taremi *et al.*, 2019; Trubiano *et al.*, 2018; Trubiano *et al.*, 2022; Wrenn *et al.*, 2017; Vyles *et al.*, 2020). In the studies with complete data on the proportion of tested patients delabeled (n = 60), 5072 were tested, of whom 4698 (92.6%) were delabeled and 76 (1.5%) were harmed; no serious reactions were reported (Appendix 7; Adkinson *et al.*, 1971; Allen *et al.*, 2020; Bauer *et al.*, 2021; Blackwell and Khan, 2020; Blumenthal *et al.*, 2019; Chen *et al.*, 2017, 2018; Chua *et al.*, 2021; Devchand *et al.*, 2019; du Plessis *et al.*, 2019; Egger *et al.*, 1997; Foolad *et al.*, 2019; Gaudreau *et al.*, 2021; Gugkaeva *et al.*, 2017; Griffith *et al.*, 2020; Ham *et al.*, 2021; Harmon *et al.*, 2020; Harper and Sanchez, 2022; Harris *et al.*, 1999; Heil *et al.*, 2016; Jones and Bland, 2017; Jones *et al.*, 2019a; Jones et al., 2019b; Kleris *et al.*, 2018; Kyi *et al.*, 2018; Leis *et al.*, 2017; Lecerf *et al.*, 2020; Lin *et al.*, 2020; Livirya *et al.*, 2022; Lnumerables and Fischer-Cartlidge, 2020; Lo *et al.*, 2020; Louden *et al.*, 2021; Maguire *et al.*, 2020; Marwood *et al.*, 2017; Mitchell *et al.*, 2021; Morjaria *et al.*, 2021; Murphy *et al.*, 2015; Parker *et al.*, 2018; Phung *et al.*, 2021; Rahbani and Monroe-Duprey, 2020; Rimawi *et al.*, 2013; Rimawi and Mazer, 2014; Savic *et al.*, 2019; Shannon and Krop, 2016; Sigona *et al.*, 2016; Smibert *et al.*, 2018; Sneddon *et al.*, 2021; Song *et al.*, 2021; Steenvoorden *et al.*, 2021; Stein *et al.*, 2020; Stone *et al.*, 2020; Taremi *et al.*, 2019; Torney and Tiberg, 2018, 2021, Trubiano *et al.*, 2017, 2018, 2022; Wall *et al.*, 2004; Wrenn *et al.*, 2017, Vyles *et al.*, 2020).

#### HCWs

A range of HCWs were involved in penA assessment: pharmacists, doctors, nurses, nurse practitioners, physician associates, medical students, and pharmacy students (Appendix 6). A total of 37 (52%) studies were multidisciplinary (Blumenthal *et al.*, 2016, 2019; Chua *et al.*, 2021; Devchand *et al.*, 2019; du Plessis *et al.*, 2019; Eischens *et al.*, 2018; Foolad *et al.*, 2019; Gaudreau *et al.*, 2021; Harper and Sanchez, 2022; Harris *et al.*, 1999; Leis *et al.*, 2017; Lecerf *et al.*, 2020; Lnumerables and Fischer-Cartlidge, 2020; Jones and Bland, 2017; Jones *et al.*, 2019a; Kleris *et al.*, 2018; Kyi *et al.*, 2018; Maguire *et al.*, 2020; Marwood *et al.*, 2017; Morjaria *et al.*, 2021; Murphy *et al.*, 2015; Patel *et al.*, 2019; Rahbani and Monroe-Duprey, 2020; Rimawi and Mazer, 2014; Savic *et al.*, 2019; Shannon and Krop, 2016; Smibert *et al.*, 2018; Sneddon *et al.*, 2021; Stone *et al.*, 2020; Taremi *et al.*, 2019; Torney and Tiberg, 2018, 2021; Trubiano *et al.*, 2017, 2018, 2022; Wall *et al.*, 2004); the rest were unidisciplinary (Adkinson *et al.*, 1971; Allen *et al.*, 2020; Bauer *et al.*, 2021; Blackwell and Khan, 2020; Chen *et al.*, 2017, 2018; Englert and Weeks, 2019; Gugkaeva *et al.*, 2017; Griffith *et al.*, 2020; Ham *et al.*, 2021; Harmon *et al.*, 2020; Heil *et al.*, 2016; Jones et al., 2019b; Lin *et al.*, 2020; Livirya *et al.*, 2022; Lo *et al.*, 2020; Louden *et al.*, 2021; Mitchell *et al.*, 2021; Nguyen *et al.*, 2019; Parker *et al.*, 2018; Phung *et al.*, 2021; Rahbani, 2019; Ravindran *et al.*, 2017; Rimawi *et al.*, 2013; Sacco *et al.*, 2019; Sigona *et al.*, 2016; Skibba *et al.*, 2014; Song *et al.*, 2021; Stein *et al.*, 2020; Steenvoorden *et al.*, 2021; Wrenn *et al.*, 2017; Wong *et al.*, 2018; Vyles *et al.*, 2020). All multidisciplinary interventions had at least one doctor. Of the unidisciplinary studies, 20 (66%) were delivered by pharmacists(Blackwell and Khan, 2020; Chen *et al.*, 2017, 2018; Englert and Weeks, 2019; Gugkaeva *et al.*, 2017; Griffith *et al.*, 2020; Ham *et al.*, 2021; Harmon *et al.*, 2020; Jones et al., 2019b; Lo *et al.*, 2020; Louden *et al.*, 2021; Mitchell *et al.*, 2021; Nguyen *et al.*, 2019; Parker *et al.*, 2018; Phung *et al.*, 2021; Rahbani, 2019; Sigona *et al.*, 2016; Song *et al.*, 2021; Skibba *et al.*, 2014; Wrenn *et al.*, 2017), 11 (34%) by doctors(Adkinson *et al.*, 1971; Allen *et al.*, 2020; Bauer *et al.*, 2021; Heil *et al.*, 2016; Lin *et al.*, 2020; Livirya *et al.*, 2022; Ravindran *et al.*, 2017; Rimawi *et al.*, 2013; Stein *et al.*, 2020; Steenvoorden *et al.*, 2021; Wood and Wisniewski, 1994; Vyles *et al.*, 2020), and one (3%) by nurses(Lecerf *et al.*, 2020).

#### Interventions

The number of intervention components in each study, grouped by EPOC category, ranged from 1 to 9 (median 5). The most frequently represented EPOC subcategory was ‘packages of care’ (58/69 studies), followed by ‘care pathway’ (44/69), and ‘educational meetings’ (36/69; Appendix 8).

### Secondary outcomes

#### Antimicrobial stewardship

A total of 42 (61%) studies reported antibiotic stewardship outcomes (Appendix 6; Blumenthal *et al.*, 2015; Chen *et al.*, 2018; Chua *et al.*, 2021; Devchand *et al.*, 2019; du Plessis *et al.*, 2019; Englert and Weeks, 2019; Foolad *et al.*, 2019; Eischens *et al.*, 2018; Gugkaeva *et al.*, 2017; Griffith *et al.*, 2020; Ham *et al.*, 2021; Harmon *et al.*, 2020; Harper and Sanchez, 2022; Harris *et al.*, 1999; Heil *et al.*, 2016; Jones *et al.*, 2019a; Kleris *et al.*, 2018; Leis *et al.*, 2017; Lin *et al.*, 2020; Lo *et al.*, 2020; Morjaria *et al.*, 2021; Phung *et al.*, 2021; Rahbani and Monroe-Duprey, 2020; Ravindran *et al.*, 2017; Parker *et al.*, 2018; Patel *et al.*, 2019; Rahbani, 2019; Sacco *et al.*, 2019; Shannon and Krop, 2016; Skibba *et al.*, 2014; Smibert *et al.*, 2018; Stein *et al.*, 2020; Stone *et al.*, 2020; Taremi *et al.*, 2019; Torney and Tiberg, 2018, 2021; Trubiano *et al.*, 2017, 2018, 2022; Wall *et al.*, 2004; Wrenn *et al.*, 2017). A total of 25 (36%; Blumenthal *et al.*, 2015; Chen *et al.*, 2018; Chua *et al.*, 2021; du Plessis *et al.*, 2019; Eischens *et al.*, 2018; Englert and Weeks, 2019; Foolad *et al.*, 2019; Griffith *et al.*, 2020; Ham *et al.*, 2021; Harris *et al.*, 1999; Jones and Bland, 2017; Jones *et al.*, 2019a; Kleris *et al.*, 2018; Leis *et al.*, 2017; Lo *et al.*, 2020; Phung *et al.*, 2021; Ravindran *et al.*, 2017; Sacco *et al.*, 2019; Steenvoorden *et al.*, 2021; Taremi *et al.*, 2019; Torney and Tiberg, 2018, 2021; Trubiano *et al.*, 2017, 2018, 2022) reported increased use of penicillin, of which 10 also reported increased cephalosporin or other beta-lactam usage (Blumenthal *et al.*, 2016; du Plessis *et al.*, 2019; Englert and Weeks, 2019; Foolad *et al.*, 2019; Ham *et al.*, 2021; Harper and Sanchez, 2022; Harris *et al.*, 1999; Jones and Bland, 2017; Ravindran *et al.*, 2017; Sacco *et al.*, 2019; Trubiano *et al.*, 2022). One study reported increased first-line antibiotic use (Eischens *et al.*, 2018). A total of 22 (33%) studies reported reductions in glycopeptides, quinolones, aztreonam, carbapenems, clindamycin, cephalosporins, macrolides, and aminoglycosides (Blumenthal *et al.*, 2016; Chua *et al.*, 2020; Devchand *et al.* 2019; Englert and Weeks, 2019; Foolad *et al.* 2019; Griffith *et al.*, 2020; Ham *et al.*, 2021; Harris *et al.* 1999; Heil *et al.* 2016; Jones and Bland 2017; Jones *et al.*, 2019a; Leis *et al.*, 2017; Morjaria *et al.*, 2021; Rahbani, 2019; Rahbani and Monroe-Duprey, 2020; Sacco *et al.*, 2019; Taremi *et al.*, 2019; Trubiano *et al.,* 2017, 2018, 2022; Torney and Tiberg, 2018; Wall *et al.,* 2004). Others reported reductions in restricted antibiotic use, more narrow-spectrum beta-lactams prescribed or given the preferred regimen (Devchand *et al.* 2019; Gugkaeva *et al.* 2017; Harper and Sanchez, 2022; Smibert *et al.*, 2018), reduced course lengths for deep seated infections, and no impact on intravenous antibiotic use (Shannon and Krop 2016).

#### Health care system impact

A total of 13 studies reported antibiotic cost savings. At the patient level, savings were reported to be between $225 to $7800 per delabeled patient (Foolad *et al.*, 2019; Jones and Bland, 2017; Jones *et al.*, 2019a; Parker *et al.*, 2018; Rimawi *et al.*, 2013). The annual hospital drug savings were reported between $12,400 and $26,000 (Harris *et al.*, 1999; Heil *et al.*, 2016) and the cost savings during the study period were reported to be between $3831 and $24,905 (Harper and Sanchez, 2022; Morjaria *et al.*, 2021; Ravindran *et al.*, 2017); one study reported savings as $74.75 per day per delabeled patient (Harmon *et al.*, 2020) and one reported reduced costs without quantification (Englert and Weeks, 2019). One study reported reduced antibiotic costs, another reported antibiotic costs to be 1.6 and 2.5 times greater for inpatient and outpatient patients allergic to penicillin, respectively (Appendix 6; du Plessis *et al.*, 2019; Englert and Weeks, 2019).

Nine studies reported staff time taken to skin test patients: an hour or less per patient (Jones *et al.*, 2019a; Leis *et al.*, 2017), between 1 and 2 hours (Chen *et al.*, 2018; Jones and Bland, 2017; Marwood *et al.*, 2017; Lo *et al.*, 2020; Morjaria *et al.*, 2021), and between 2 and 2.5 hours (Torney and Tiberg, 2021) and one study reported the time requirement as 0.15 full-time equivalent pharmacist, with 30 minutes a week of pharmacy technician time (Gaudreau *et al.*, 2021). The time to delabel on history alone was between 5 and 15 minutes (Louden *et al.*, 2021; Nguyen *et al.*, 2019; Song *et al.*, 2021; Appendix 6).

Three studies reported the cost of ST to be between $137 and $175 (Harmon *et al.*, 2020; Jones *et al.*, 2019a; Lo *et al.*, 2020), and one reported no increased costs due to absorption by programmatic resources (Morjaria *et al.*, 2021). The cost of DPT is reported to be 35.18 Australian dollars, and direct delabel to have no cost implications (Chua *et al.*, 2021).

Hospital length of stay was reported to be reduced (du Plessis *et al.*, 2019; Gugkaeva *et al.*, 2017; Parker *et al.*, 2018), increased (Vyles *et al.*, 2020), and not affected by PADL (Chua *et al.*, 2021; Leis *et al.*, 2017; Sacco *et al.*, 2019; Shannon and Krop, 2016). Mortality and readmission rates were unchanged (Chua *et al.*, 2021; Harper and Sanchez, 2022; Leis *et al.*, 2017; Shannon and Krop, 2016; Trubiano *et al.*, 2022), as were adverse drug events (Leis *et al.*, 2017; Shannon and Krop, 2016).

## Meta-analysis

### Direct delabeling on history alone on history alone

#### Assessed for delabel through direct delabeling on history alone

A total of 11 studies had a low risk of bias (Bauer *et al.*, 2021; Chua *et al.*, 2021; Devchand *et al.*, 2019; du Plessis *et al.*, 2019; Gaudreau *et al.*, 2021; Griffith *et al.*, 2020; Livirya *et al.*, 2022; Louden *et al.*, 2021; Mitchell *et al.*, 2021; Shannon and Krop, 2016; Song *et al.*, 2021; Taremi *et al.*, 2019) and six had a moderate risk of bias (Harper and Sanchez, 2022; Jones *et al.*, 2019a; Lecerf *et al.*, 2020, Lo *et al.*, 2020; Murphy *et al.*, 2015; Nguyen *et al.*, 2019). Six studies with incomplete data or a high risk of bias were excluded(Ham *et al.*, 2021; Jones et al., 2019b; Patel *et al.*, 2019; Rahbani, 2019; Sacco *et al.*, 2019; Wall *et al.*, 2004). In the meta-analysis, 4350 patients were assessed, of whom 689 (15.8%) were successfully delabeled. The proportion of assessed patients delabeled was 14% (95% confidence interval [CI]; 9.0-21%), and the study heterogeneity was high (I^2^ = 97%, X^2^_17_ ≤0.01; Appendix 9), with evidence of publication bias (Egger test *P*-value = 0.2087; Appendix 10).

#### Appropriate for delabeling through history alone

A total of 12 studies had a low risk of bias (Bauer *et al.*, 2021; Chua *et al.*, 2021; Devchand *et al.*, 2019; du Plessis *et al.*, 2019; Gaudreau *et al.*, 2021; Griffith *et al.*, 2020; Harper and Sanchez, 2022; Livirya *et al.*, 2022; Louden *et al.*, 2021; Mitchell *et al.*, 2021; Song *et al.*, 2021; Taremi *et al.*, 2019) and seven had a moderate risk of bias (Ham *et al.*, 2021; Jones *et al.*, 2019a; Lecerf *et al.*, 2020; Lo *et al.*, 2020; Murphy *et al.*, 2015; Wall *et al.*, 2004). Five studies with incomplete data or a high risk of bias were excluded (Jones *et al.*, 2019a; Nguyen *et al.*, 2019; Patel *et al.*, 2019; Rahbani, 2019; Sacco *et al.*, 2019). Of 713 patients suitable for DDL, 701 (100%; 95% CI 99-100%) were successfully delabeled, with no reports of harm. The study heterogeneity was high (I^2^ = 63%, X^2^_18_ ≤0.01; Appendix 9), and the risk of publication bias low (Egger test *P*-value = 0.0001; Appendix 10).

### Direct DPT

#### Assessed for direct DPT

A total of 15 studies had a low risk of bias (Allen *et al.*, 2020; Bauer *et al.*, 2021; Chua *et al.*, 2021; Devchand *et al.*, 2019; du Plessis *et al.*, 2019; Gaudreau *et al.*, 2021; Harper and Sanchez, 2022; Lin *et al.*, 2020; Livirya *et al.*, 2022; Phung *et al.*, 2021; Savic *et al.*, 2019; Sneddon *et al.*, 2021; Stone *et al.*; 2020; Steenvoorden *et al.*, 2021; Trubiano *et al.*, 2018) and four had a moderate risk of bias (Kyi *et al.*, 2018; Lecerf *et al.*, 2020; Murphy *et al.*, 2015; Sigona *et al.*, 2016). A total of 13 studies with incomplete data or a high risk of bias were excluded (Blumenthal *et al.*, 2016, 2019; Ham *et al.*, 2021; Jones et al., 2019b; Maguire *et al.*, 2020; Patel *et al.*, 2019; Sacco *et al.*, 2019; Smibert *et al.*, 2018; Stein *et al.*, 2020; Trubiano *et al.*, 2017, 2022; Wong *et al.*, 2018; Vyles *et al.*, 2020). Of 4207 patients assessed, 844 (27%; 95% CI 18-37%) were successfully delabeled. The study heterogeneity was high (I^2^ = 98%, X^2^_16_ ≤0.01; Appendix 9), and the risk of publication bias high (Egger test *P*-value = 0.3452; Appendix 10).

#### Tested by direct DPT

A total of 16 had a low risk of bias (Allen *et al.*, 2020; Bauer *et al.*, 2021; Blumenthal *et al.*, 2019; Chua *et al.*, 2021; Devchand *et al.*, 2019; du Plessis *et al.*, 2019; Harper and Sanchez, 2022; Lin *et al.*, 2020; Livirya *et al.*, 2022; Phung *et al.*, 2021; Savic *et al.*, 2019; Stone *et al.*, 2020; Smibert *et al.*, 2018; Sneddon *et al.*, 2021; Steenvoorden *et al.*, 2021; Trubiano *et al.*, 2018) and eight had a moderate risk of bias (Ham *et al.*, 2021; Kyi *et al.*, 2018; Lecerf *et al.*, 2020; Maguire *et al.*, 2020; Murphy *et al.*, 2015; Sigona *et al.*, 2016; Stein *et al.*, 2020; Trubiano *et al.*, 2022). Seven studies with incomplete data or a high risk of bias were excluded (Blumenthal *et al.*, 2016; Jones et al., 2019b; Patel *et al.*, 2019; Sacco *et al.*, 2019; Trubiano *et al.*, 2017; Wong *et al.*, 2018; Vyles *et al.*, 2020). Of 1336 patients tested, 1288 (98%; 95% CI 97-99%) were successfully delabeled. The study heterogeneity was low (I^2^ = 0%, X^2^22 20.29 (*P* = 0.56); Appendix 9) and the risk of publication bias high (Egger test *P*-value = 0.1574; Appendix 10).

#### Harmed by direct DPT

A total of 16 had a low risk of bias (Allen *et al.*, 2020; Bauer *et al.*, 2021; Blumenthal *et al.*, 2019; Chua *et al.*, 2021; Devchand *et al.*, 2019; du Plessis *et al.*, 2019; Harper and Sanchez, 2022; Lin *et al.*, 2020; Livirya *et al.*, 2022; Phung *et al.*, 2021; Savic *et al.*, 2019; Smibert *et al.*, 2018; Sneddon *et al.*, 2021; Steenvoorden *et al.*, 2021; Stone *et al.*, 2020; Trubiano *et al.*, 2018) and nine had a moderate risk of bias (Ham *et al.*, 2021; Kyi *et al.*, 2018; Lecerf *et al.*, 2020; Maguire *et al.*, 2020; Murphy *et al.*, 2015; Sigona *et al.*, 2016; Stein *et al.*, 2020; Trubiano *et al.*, 2022; Vyles *et al.*, 2020). Six studies with incomplete data or a high risk of bias were excluded (Blumenthal *et al.*, 2015; Jones et al., 2019b; Patel *et al.*, 2019; Sacco *et al.*, 2019; Trubiano *et al.*, 2017; Wong *et al.*, 2018). Of 1376 patients tested, 38 (1%;95% CI 0-2%) were harmed. The study heterogeneity was low (I^2^ = 0%, X^2^_24_ = 0.59; Appendix 9) and the risk of publication bias high (Egger test *P*-value = 0.1646; Appendix 10).

### ST, followed by DPT

#### Assessed for delabel through skin testing/DPT

A total of 12 studies had a low risk of bias (Chen *et al.*, 2017, 2018; Devchand *et al.*, 2019; Foolad *et al.*, 2019; Gaudreau *et al.*, 2021; Harmon *et al.*, 2020; Harper and Sanchez, 2022; Leis *et al.*, 2017; Marwood *et al.*, 2017; Rimawi *et al.*, 2013; Rimawi and Mazer, 2014; Taremi *et al.*, 2019) and two had a moderate risk of bias (Adkinson *et al.*, 1971; Lo *et al.*, 2020). Nine studies with incomplete data or a high risk of bias were excluded (Gugkaeva *et al.*, 2017; Ham *et al.*, 2021; Kleris *et al.*, 2018; Lnumerables and Fischer-Cartlidge, 2020; Morjaria *et al.*, 2021; Ravindran *et al.*, 2017; Trubiano *et al.*, 2017; Torney and Tiberg, 2021; Wall *et al.*, 2004). Of 2890 patients assessed, 925 (41%; 95% CI 24-59%) were successfully delabeled. The study heterogeneity was high (I^2^ = 99%, X^2^_13_ = 1161.19 (*P* <0.01; Appendix 9) and the risk of publication bias high (Egger test *P*-value=0.4934; Appendix 10).

#### Tested by skin testing/DPT

A total of 14 studies had a low risk of bias (Chen *et al.*, 2017, 2018; Devchand *et al.*, 2019; Foolad *et al.*, 2019; Gaudreau *et al.*, 2021; Harmon *et al.*, 2020; Harper and Sanchez, 2022; Leis *et al.*, 2017; Lo *et al.*, 2020; Marwood *et al.*, 2017; Rimawi *et al.*, 2013; Rimawi and Mazer, 2014; Taremi *et al.*, 2019; Torney and Tiberg, 2021) and five had a moderate risk of bias (Adkinson *et al.*, 1971; Gugkaeva *et al.*, 2017; Ham *et al.*, 2021; Kleris *et al.*, 2018; Morjaria *et al.*, 2021). Four studies with incomplete data or high risk of bias were excluded (Lnumerables and Fischer-Cartlidge, 2020; Ravindran *et al.*, 2017; Trubiano *et al.*, 2017; Wall *et al.*, 2004). Of 1294 patients tested, 1177 (95.0%; 95% CI 90-99%) were successfully delabeled. The study heterogeneity was high (I^2^ = 87%, X^2^_18_ = 138.65 (*P* <0.01; Appendix 9) and the risk of publication bias low (Egger test *P*-value = 0.0199; Appendix 10).

#### Harmed by skin testing/DPT

A total of 13 studies had a low risk of bias (Chen *et al.*, 2017, 2018; Devchand *et al.*, 2019; Foolad *et al.*, 2019; Gaudreau *et al.*, 2021; Harmon *et al.*, 2020; Harper and Sanchez, 2022; Leis *et al.*, 2017; Marwood *et al.*, 2017; Rimawi *et al.*, 2013; Rimawi and Mazer, 2014; Taremi *et al.*, 2019; Torney and Tiberg, 2021) and eight had a moderate risk of bias (Adkinson *et al.*, 1971; Gugkaeva *et al.*, 2017; Ham *et al.*, 2021; Kleris *et al.*, 2018; Lnumerables and Fischer-Cartlidge, 2020; Lo *et al.*, 2020; Morjaria *et al.*, 2021; Wall *et al.*, 2004) Four studies with incomplete data or high risk of bias were excluded (Blackwell and Khan, 2020; Jones et al., 2019b; Ravindran *et al.*, 2017; Trubiano *et al.*, 2017). Of 1464 patients tested, 19 were harmed (0%; 95% CI 0-1%). The study heterogeneity was low (I^2^ = 21% X^2^_20_ = 25.31 [*P*-value = 0.09]; Appendix 9) and the risk of publication bias was low (Egger test *P*-value = 0.0166; Appendix 10).

Heterogeneity remained unchanged after the sensitivity analysis, except for the proportion of patients delabeled on history alone (Appendix 11). The extraction check by a second reviewer idenfied 3.8% error in data extraction (see appendix 12).

## Discussion

The rates of PADL varied from 14% to 41%, depending on the penA assessment method. Less intensive methods that targeted the smaller population of lowest risk patients delabeled a smaller proportion than those using more formal testing and included higher risk patients. Once patients were assessed as suitable for delabeling, the rates of PADL were high (≥95%), indicating good acceptability of testing and results. PenA assessment by nonallergists was delivered by a diverse workforce to a diverse patient population and demonstrated the significant opportunity to reduce erroneous penA labels, in line with global antibiotic stewardship ambitions (Australian Drug Allergy Committee 2020; Jeimy *et al.*, 2020; Shenoy *et al.*, 2019; Sneddon *et al.*, 2021; World Health Organization, 2021). This review found that penA assessment by nonallergists was safe: of the tested patients, 1.7% had a subsequent reaction, but none were serious.

PADL increased penicillin use and reduced nonpenicillin use, such as quinolones and aztreonam, with associated reduced antibiotic costs. HCW time taken to delabel varied depending on the testing method. Local PADL interventions might need to balance the staff resource available with the potential impact on patient care by prioritizing patients according to greatest need or where PADL has the greatest potential for improved patient care or health system impact (Macy and Contreras, 2014). The potential antibiotic cost savings are likely to offset the HCW and the ST costs (Macy and Contreras, 2014), but the HCW costs are often not/poorly described. PADL is delivered by HCWs and their time has an inherent cost that needs to be adequately described to enable appropriate health-economic analysis. The wider and longer-term impact of PADL, due not only to reduced drug acquisition costs but also savings in terms of potential reductions in length of stay and mortality, are estimated to have been 10 times the cost of allergy testing (Macy and Contreras, 2014; Macy and Shu, 2017). The longer-term impact of PADL on patient, health systems, and antimicrobial resistance requires further study.

Most interventions protocolized penA assessment, with allergists contributing to the development of protocols. The low number of studies reporting direct access to an allergy expert during the day-to-running of PADL provides reassurance of the effectiveness/safety of these protocols without an allergist present. Education was a key theme supporting the appropriate use of the testing protocols.

PADL was commonly delivered by a small team or an individual HCW as an outreach service and always in the hospital setting. Less commonly, the responsible medical team delabeled patients. Individual HCW or small teams limit the reach of PADL across a hospital. The advantage of small teams or individual delivery of PADL is a greater likelihood of the requisite knowledge and motivation, but the delivery of PADL by the wider workforce may enable a broader reach across the hospital. Adequate knowledge, motivation, and competing demands may hinder the delivery of PADL by the wider workforce. Quality improvement of the methodology (Bauer *et al.*, 2021; Louden *et al.*, 2021) and financial incentives (Bauer *et al.*, 2021) have been used to motivate staff, but this adds further expense and time resource to PADL. Whether PADL is safer and more effective as a small team/individual or delivered by the wider workforce needs further study, and the barriers/enablers to the delivery of PADL at large scale need exploration. Given the safety of direct DPT in low-risk patients, there is a potential to extend this to health care settings outside of the hospital, but this requires further study.

There was high heterogeneity between studies, with several possible explanations. Risk stratification before testing was done on both patient factors and allergy history, which varied between studies. The route of DPT administration, location of testing, and HCW(s) undertaking testing also varied. Others have reported oral challenges to be better tolerated than intravenous challenges, challenges in the inpatient setting more likely to be tolerated than in the ambulatory setting, and tolerance in children were reported to be higher than in adults; although, tolerance was reported to be similar between those with and without infection (DesBiens *et al.*, 2020; Harandian *et al.*, 2016). Some studies only assessed using one method and some studies used all three assessment methods, introducing further potential for heterogeneity. The optimization of testing protocols requires further study and harmonization.

We found low heterogeneity between studies assessing the proportion of tested patients who were successfully delabeled and the proportion harmed by DDPT. There was high heterogeneity between studies looking at PADL in those identified suitable for DDL, but after the sensitivity analysis and removal of one study, the recalculated heterogeneity was low. A similar systematic review of the literature, not restricted to nonallergists, reported the successful delabeling of 595 (97%) patients using DDPT and were comparable to our findings providing external validity to these data (DesBiens *et al.*, 2020). We report harm after DDPT to be 2%, comparable to the expected 0.5-2% adverse drug reaction (ADR) rate in patients without a history of penA but lower than other direct DPT studies (DesBiens *et al.*, 2020; Shenoy *et al.*, 2019). We found low heterogeneity between ST/DPT studies when looking at harm from delabeling, but the heterogeneity was high between studies looking at the proportion of tested patients delabeled by ST/DPT. We found the rate of harm to be lower in our study than other studies reporting penicillin tolerability after ST/DPT (1% vs 6%), which may be explained by allergists testing higher risk patients or higher rates of false-positive skin in some studies or differing definitions of harm (DesBiens *et al.*, 2020).

### Limitations

All the studies are from high-income countries (70% from the United States); therefore, the findings may not be generalizable to low- and middle-income countries. However, the proportion of tested patients delabeled and adverse event rates are similar across studies with data from eight countries.

Most studies were case series, with inherent patient selection bias, and the inclusion of conference abstracts limited the review of methodology. Conference abstracts are limited by the extent of reporting and quality (Scherer and Saldanha, 2019). However, the inclusion of abstracts gives a wider and more representative view of the nonallergist delabel activity, which is particularly important because full paper publication of conference abstracts is reported to be low (Scherer and Saldanha, 2019). The high heterogeneity between studies limits the certainty of our findings.

To reduce publication bias, we searched trial registries, unpublished studies, and the bibliographies of included studies and asked known experts in the field for missing studies. Despite this, five of eight funnel plots identified a high risk of publication bias.

The rate of side effects was reported in those delabeled on history alone. Given that the background rate for a penicillin reaction is 0.5-2% (Shenoy *et al.*, 2019), we would expect to see some evidence of harm in the 812 patients delabeled on history alone upon subsequent penicillin re-exposure. It was not clear how many patients went on to receive penicillin after delabeling. The rate of harm in this patient population requires further study.

The statistical power of the I^2^ test is limited in meta-analyses with <20 studies and/or with an average study sample size of <80, with all the meta-analyses in this study below this threshold (Huedo-Medina *et al.*, 2006).

## Conclusion

Nonallergists have used several approaches to assess and PADL, all of which appear to be effective and safe. More comprehensive testing capability allowed a greater proportion of assessed patients to be delabeled. A diverse workforce has delivered penA assessment services outside of allergy/immunology services. The consequences of PADL were reported to be increased use of penicillin and other beta-lactams, with a subsequent reduction in nonbeta-lactam antibiotic use and reduced antibiotic drug costs. PADL is often limited to individual HCWs or small groups of HCWs within a hospital, predominantly delivered as an outreach service, which limits the impact of PADL. The delivery of PADL by the primary health care provider and extending PADL to health care settings outside the hospital will broaden the impact of PADL. A few studies showed provider-delivered PADL to be safe and effective but further studies are required on the hospital-wide implementation of PADL delivered by primary provider teams. The studies were from high-income countries, and data are also needed from low- and middle-income countries.

## Funding

This study was funded by HEE/NIHR ICA Programme Clinical Doctoral Research Fellowship; source of funding: NIHR300542 September 1, 2020. The funder had no further role in the study.

## Ethical approval

The study does not require ethical approval because the meta-analysis is based on published research and the original data are anonymous.

## Acknowledgments

The authors would like to thank Katy Oak, outreach librarian of the Knowledge Spa, Royal Cornwall Hospitals Trust, Truro, for her help in developing the search strategy for this review; Chris Johns, information specialist of the University of Plymouth; and Natalie King, information specialist of Leeds Institute of Health Sciences, for their technical support.

## Author contributions

Screening title and abstracts NP, JS, DK, RO, SA, MU, BK, STC, JS Full paper screening NP, RO Data extraction NP, BK statistics MU Extraction check JS Manuscript review All authors.

## Declaration of Competing Interest

The authors have no competing interests to declare.

## Appendix Supplementary materials

Image, application 1

Image, application 2
