# Supplementary material for: The effectiveness of interventions that support penicillin allergy assessment and delabeling of adult and pediatric patients by nonallergy specialists: a systematic review and meta-analysis
Source: Int J Infect Dis. 2023 Apr;129:152–61. doi: 10.1016/j.ijid.2022.11.026 (PMC10017351; doi:10.1016/j.ijid.2022.11.026)
Supplement: Supplementary file 2 [file mmc2.docx]

**Appendices**

**Appendix 1: Search strategy**

An initial limited search of EMBASE (Ovid) was undertaken (NP) to identify articles on the topic. The text words contained in the titles and abstracts of relevant articles, and the index terms used to describe the articles were used to develop a full search strategy for EMBASE (NP) (see Appendix 1). The search strategy, including all identified keywords and index terms, were adapted for the following databases (NP): MEDLINE (Ovid), CINAHL (Ovid), PsycInfo, Web of Science and Cochrane CENTRAL and the grey literature including: WHO Library database, conference proceedings (ESCMID, Society for Healthcare Epidemiology of America, Healthcare Infection Society and Infection Prevention Society), registered Controlled Trial Registers, technical or research reports from government agencies and the British Library (Ethos) Collection of PhD dissertations. Databases were searched from inception to 21^st^ January 2022 (NP). EMBASE (Ovid) MEDLINE (Ovid), CINAHL (Ovid), PsycInfo, Web of Science and Cochrane CENTRAL. We contacted known experts in the topic to ensure we have not overlooked relevant literature. The search strategy was reviewed by an experienced information specialist (KO). Search terms included index terms as well as keywords for the concepts penicillin allergy, assessment and de-labelling. Backwards reference searches of all included sources of evidence were completed to identify additional studies (NP). Only studies published in English were included due to a lack of funding for translation services. No date limit was set for included studies.

All identified citations were collated and uploaded into Endnote Note v.X9.2 (Clarivate Analytics, PA, USA)^105^ and duplicates removed. Following a pilot test, titles and abstracts were screened by at least two independent reviewers (NP, SA, DK, RO, JS) for assessment against the inclusion criteria for the review using RAYYAN software.^20^ Potentially relevant studies were retrieved in full and their citation details imported into the JBI System for the Unified Management, Assessment and Review of Information (JBI SUMARI) (JBI, Adelaide, Australia).^106^ The full text of selected citations were assessed in detail against the inclusion criteria by two independent reviewers (NP, RO) using RAYYAN software.^20^ Reasons for full text exclusion are reported.

Search conducted on Embase in NICE Healthcare Databases Advances Search platform:

"((penicillin ADJ2 allerg*).ti,ab OR (penicillin ADJ2 hypersensitiv*).ti,ab OR (penicillin ADJ2 anaphylaxis).ti,ab OR (beta-lactam ADJ2 allerg*).ti,ab OR ("beta-lactam" ADJ2 hypersensitiv*).ti,ab OR ("beta-lactam" ADJ2 anaphylaxis*).ti,ab OR ("betalactam" ADJ2 anaphylaxis*).ti,ab OR ("betalactam" ADJ2 hypersensitiv*).ti,ab OR ("betalactam" ADJ2 allerg*).ti,ab OR ("*lactam" ADJ2 allerg*).ti,ab OR ("*lactam" ADJ2 hypersensitiv*).ti,ab OR ("*lactam" ADJ2 anaphylaxis*).ti,ab OR ("antibiotic" ADJ2 anaphylaxis*).ti,ab OR ("antibiotic" ADJ2 hypersensitiv*).ti,ab OR ("antibiotic" ADJ2 allerg*).ti,ab OR ("antimicrobial" ADJ2 allerg*).ti,ab OR ("antimicrobial" ADJ2 hypersensitiv*).ti,ab OR ("antimicrobial" ADJ2 anaphylaxis*).ti,ab OR "PENICILLIN ALLERGY"/ OR ("PENICILLIN DERIVATIVE"/ AND "DRUG HYPERSENSITIVITY"/)) AND (("clinical decision tool").ti,ab OR ("clinical decision making").ti,ab OR ("clinical assessment tool").ti,ab OR ((direct).ti,ab AND (challenge).ti,ab) OR (de-label).ti,ab OR (delabel).ti,ab OR ("test dose").ti,ab OR ("interview").ti,ab OR ("antibiotic stewardship").ti,ab OR ("antimicrobial stewardship").ti,ab OR ("test*").ti,ab OR ("allergy test*").ti,ab OR ("skin test*").ti,ab OR ("assessment").ti,ab OR ("allergy assessment").ti,ab OR ("oral challenge").ti,ab OR ("provocation test").ti,ab OR "ANTIMICROBIAL STEWARDSHIP"/ OR "SKIN TEST"/ OR "PROVOCATION TEST"/ OR "ALLERGY TEST"/)"

**Appendix 2: Included studies**

Adkinson NF, Thompson WL, Maddrey WC, Lichtenstein LM. Routine use of penicillin skin testing on an inpatient service. The New England journal of medicine. 1971;285(1):22–4.

Allen HI, Vazquez-Ortiz M, Murphy AW, Moylett EM. De-labeling penicillin-allergic children in outpatients using telemedicine: Potential to replicate in primary care. The journal of allergy and clinical immunology In practice. 2020;8(5):1750–2.

Bauer ME, MacBrayne C, Stein A, et al. A Multidisciplinary Quality Improvement Initiative to Facilitate Penicillin Allergy Delabeling Among Hospitalized Pediatric Patients. Hospital pediatrics 2021; 11(5): 427-34.

Blackwell W, Khan D. Penicillin Allergy Testing by Allergy Trained Pharmacists in Hospitalized Patients. Journal of allergy and clinical immunology. 2020;145(2):AB161.

Blumenthal KG, Li Y, Hsu JT, Wolfson AR, Berkowitz DN, Carballo VA, et al. Outcomes from an inpatient beta-lactam allergy guideline across a large US health system. Infection control and hospital epidemiology. 2019;40(5):528–35.

Blumenthal KG, Shenoy ES, Varughese C, Hurwitz S, Hooper D, Banerji A. Impact of a clinical guideline for prescribing antibiotics to inpatients with reported penicillin or cephalosporin allergies. Journal of allergy and clinical immunology. 2015;135(2):AB232.

Chen JR, Tarver SA, Alvarez KS, Tran T, Khan DA. A Proactive Approach to Penicillin Allergy Testing in Hospitalized Patients. The journal of allergy and clinical immunology In practice. 2017;5(3):686–93.

Chen JR, Tarver SA, Alvarez KS, Wei W, Khan DA. Improving Aztreonam Stewardship and Cost Through a Penicillin Allergy Testing Clinical Guideline. Open forum infectious diseases. 2018;5(6):ofy106~~.~~

Chua KYL, Vogrin S, Bury S, Douglas A, Holmes NE, Tan N, et al. The Penicillin Allergy Delabeling Program: A Multicenter Whole-of-Hospital Health Services Intervention and Comparative Effectiveness Study. Clinical infectious diseases : an official publication of the Infectious Diseases Society of America. 2020;

Devchand, M., Kirkpatrick CMJ, Stevenson W, Garrett K, Perera D, et al. Evaluation of a pharmacist-led penicillin allergy de-labelling ward round: a novel antimicrobial stewardship intervention. The Journal of antimicrobial chemotherapy. 2019;74(6):1725–30.

du Plessis T, Walls G, Jordan A, Holl, J. D. Implementation of a pharmacist-led penicillin allergy de-labelling service in a public hospital. The Journal of antimicrobial chemotherapy. 2019;74(5):1438–46.

Eischens MR, Wolf LM, Dumkow LE, Anderson AM, Jameson AP, Br, et al. Impact of an Emergency Department Antimicrobial Stewardship Program on the Rate of Beta-Lactam Allergy Challenge. Annals of emergency medicine. 72(4):S61–S61.

Englert E, Weeks A. Pharmacist-driven penicillin skin testing service for adults prescribed nonpreferred antibiotics in a community hospital. American journal of health-system pharmacy : AJHP : official journal of the American Society of Health-System Pharmacists. 2019;76(24):2060–9.

Foolad F, Berlin S, White C, ice, Dishner E, Jiang Y, et al. The Impact of Penicillin Skin Testing on Aztreonam Stewardship and Cost Savings in Immunocompromised Cancer Patients. Open forum infectious diseases. 2019;6(10):ofz371.

Gaudreau S, Bourque G, Cote K, et al. Resources Assessment for Penicillin Allergy Testing Performed by Pharmacists at the Patient's Bedside. The Annals of pharmacotherapy 2021: 10600280211002412.

Griffith NC, Justo JA, Winders HR, Al-Hasan MN, Mediwala KN, Bookstaver PB. Regulatory approval, implementation, and brief assessment of a pharmacist- and pharmacy trainee-administered penicillin allergy assessment and skin testing program. JACCP Journal of the American College of Clinical Pharmacy. 2020;

Gugkaeva Z, Crago JS, Yasnogorodsky M. Next step in antibiotic stewardship: Pharmacist-provided penicillin allergy testing. Journal of clinical pharmacy and therapeutics. 2017;42(4):509–12.

Ham Y, Sukerman ES, Lewis JS, Tucker KJ, Yu DL, Joshi SR. Safety and efficacy of direct two-step penicillin challenges with an inpatient pharmacist-driven allergy evaluation. Allergy and Asthma Proceedings 2021; 42(2): 153-9.

Harmon S, Richardson T, Simons H, Monforte S, Fanning S, Harrington K. The Clinical and Financial Impact of a Pharmacist-Driven Penicillin Skin Testing Program on Antimicrobial Stewardship Practices. Hospital pharmacy. 2020;55(1):58–63.

Harper HM, Sanchez M. Review of Pharmacist Driven Penicillin Allergy Assessments and Skin Testing: A Multi-Center Case-Series. Hospital Pharmacy

Harris AD, Sauberman L, Kabbash L, Greineder DK, Samore MH. Penicillin skin testing: a way to optimize antibiotic utilization. The American journal of medicine. 1999;107(2):166–8.

Heil EL, Bork JT, Schmalzle SA, Kleinberg M, Kewalramani A, Gilliam BL, et al. Implementation of an Infectious Disease Fellow-Managed Penicillin Allergy Skin Testing Service. Open forum infectious diseases. 2016;3(3):ofw155.

Jones BM, Avramovski N, Concepcion AM, Crosby J, Bl, M C. Clinical and Economic Outcomes of Penicillin Skin Testing as an Antimicrobial Stewardship Initiative in a Community Health System. Open forum infectious diseases. 2019;6(4):ofz109.

Jones BM, Bland, M. C. Penicillin skin testing as an antimicrobial stewardship initiative. American journal of health-system pharmacy : AJHP : official journal of the American Society of Health-System Pharmacists. 2017;74(4):232–7.

Jones BM, Gamble K, Sizemore S, Bl, M. C. The impact of pharmacy students performing penicillin allergy reconciliation in a community health system. Open forum infectious diseases. 2019;6:S351.

Kleris R, Sarubbi C, Wrenn R, Anderson D, Lugar PL. Inpatient penicillin allergy evaluation program enriches anti-microbial stewardship aims. Journal of allergy and clinical immunology. 2018;141(2):AB31.

Kyi L, Heke E, McPhee S, Ojaimi S, Barnes S. Direct oral challenge for rapid penicillin de-labelling in acute admitted general medical patients. Internal medicine journal. 2018;48:13.

Lecerf K, Chaparro J, Hehmeyer J, Hussain C, Macias C, Vegh M, et al. Development of a Penicillin Allergy Electronic Decision Support Pathway for Pediatric Inpatient Admissions. Journal of allergy and clinical immunology. 2020;145(2):AB99.

Leis, Palmay L, Ho G, Raybardhan S, Gill S, Kan T, et al. Point-of-Care β-Lactam Allergy Skin Testing by Antimicrobial Stewardship Programs: A Pragmatic Multicenter Prospective Evaluation. Clinical infectious diseases. 2017;65(7):1059–65.

Lin L, Nagtegaal JE, Buijtels PCAM, Jong E. Antimicrobial stewardship intervention: optimizing antibiotic treatment in hospitalized patients with reported antibiotic allergy. The Journal of hospital infection. 2020;104(2):137–43.

Livirya S, Pithie A, Chua I, Hamilton N, Doogue M, Isenman H. Oral amoxicillin challenge for low risk penicillin allergic patients. Internal medicine journal. 2020;

Lnumerables F, Fischer-Cartlidge E. IMPROVING ANTIBIOTIC STEWARDSHIP THROUGH NURSE-DRIVEN PENICILLIN ALLERGY TESTING. Oncology Nursing Forum. 47(2).

Lo SCR, Lacaria K, Mah A, Wong T, Mak R. An algorithm-based approach to routinely delabel penicillin allergy in pre-hematopoietic stem cell transplant patients with low risk of reaction. Allergy, Asthma and Clinical Immunology. 2020;16.

Louden NJ, Hansen LA, Rimal A, Norton LE. Implementation of a Pharmacist-Driven Penicillin and Cephalosporin Allergy Assessment Tool: A Pilot Evaluation. The journal of pediatric pharmacology and therapeutics : JPPT : the official journal of PPAG 2021; 26(7): 696-701.

Maguire M, Hayes BD, Fuh L, Elshaboury R, hi RG, Bor S, et al. Beta-lactam antibiotic test doses in the emergency department. The World Allergy Organization journal. 2020;13(1):100093.

Marwood J, Aguirrebarrena G, Kerr S, Welch SA, Rimmer J. De-labelling self-reported penicillin allergy within the emergency department through the use of skin tests and oral drug provocation testing. Emergency medicine Australasia : EMA. 2017;29(5):509–15.

Mitchell AB, Ness RA, Bennett JG, et al. Implementation and Impact of a beta-Lactam Allergy Assessment Protocol in a Veteran Population. Federal practitioner : for the health care professionals of the VA, DoD, and PHS 2021; 38(9): 420-5.

Morjaria S, Inumerables F, Patel D, et al. Penicillin Allergy Testing: An Outpatient Nurse-Driven Program for Patients With Cancer. Clinical journal of oncology nursing 2021; 25(2): 143-50.

Murphy K, Scanlan B, Coghlan D. Does this child really have a penicillin allergy? Irish Medical Journal. 2015;108(4).

Nguyen CT, Sahbani O, Pisano J, Pursell K, Pettit NN. Impact of a standardized pharmacist-led beta-lactam allergy interview on the quality of allergy documentation. Open forum infectious diseases. 2019;6:S353.

Parker N, Choo HF, Ghodrat M. Implementation of pharmacist-driven penicillin allergy skin testing in a community hospital resulting in a change in scope of practice for pharmacists. JACCP Journal of the American College of Clinical Pharmacy. 2018;1(2):312–3.

Patel R, Saccone N, Stock K, Utley S, Bouknight D. Challenging penicillin allergies: Pharmacist led program in a community hospital. Open forum infectious diseases. 2019;6: S351.

Phung M, Vo T, Murfin B, Galbraith K, Barnes S, Coutsouvelis J. Pharmacist-led penicillin allergic risk categorisation in intensive care patients for the purpose of rapid penicillin allergy delabeling (rapid phire) pilot study. Internal Medicine Journal 2021; 51(SUPPL 4): 13.

Rahbani P. A quality improvement initiative to increase penicillin allergy clarification and decrease aztreonam usage. Open forum infectious diseases. 2019;6: S350.

Rahbani P, Monroe-Duprey L. Clinical Outcome of Penicillin Skin Testing as an Antimicrobial Stewardship Initiative in the pre-surgical clinic in a community hospital. Open Forum Infectious Diseases 2020; 7(SUPPL 1): S683.

Ravindran S, Beshir M, Wang S, i S, Hanson A, O’Driscoll T, et al. Impact of hospital-wide guideline for antimicrobial stewardship in patients with history of beta-lactam allergy at an academic medical center. Journal of allergy and clinical immunology. 2017;139(2): AB29.

Rimawi RH, Cook PP, Gooch M, Kabchi B, Ashraf MS, Rimawi BH, et al. The impact of penicillin skin testing on clinical practice and antimicrobial stewardship. Journal of hospital medicine. 2013;8(6):341–5.

Rimawi RH, Mazer MA. Expanding the pool of healthcare providers to perform penicillin skin testing in the ICU. Intensive care medicine. 2014;40(3):462–3.

Sacco KA, Cochran BP, Epps K, Parkulo M, Gonzalez-Estrada A. Inpatient beta-lactam test-dose protocol and antimicrobial stewardship in patients with a history of penicillin allergy. Annals of allergy, asthma & immunology: official publication of the American College of Allergy, Asthma, & Immunology. 2019;122(2):184–8.

Savic L, Gurr L, Kaura V, Toolan J, Sandoe JAT, Hopkins PM, et al. Penicillin allergy de-labelling ahead of elective surgery: feasibility and barriers. British journal of anaesthesia. 2019;123(1):e110–6.

Shannon KT, Krop LC. Evaluation of the Implementation of an Allergy Assessment Tool as an Antimicrobial Stewardship Initiative. Infectious Diseases in Clinical Practice. 2016;24(6):332–6.

Sigona NS, Steele JM, Miller CD. Impact of a pharmacist-driven beta-lactam allergy interview on inpatient antimicrobial therapy: A pilot project. Journal of the American Pharmacists Association: JAPhA. 2016;56(6):665–9.

Skibba N, Fischer J, Loecker B. Pilot program of pharmacist managed penicillin allergy skin testing on inpatients at a medical center to determine cost-benefit. Pharmacotherapy. 34(6):E115–E115.

Smibert O, Douglas A, Devch, M., Lambros B, Stevenson W, et al. The safety and efcacy of an oral penicillin rechallenge program in cancer patients: A pilot multicenter study. Open forum infectious diseases. 2018;5:S506.

Song Y-C, Nelson ZJ, Wankum MA, Gens KD. Effectiveness and Feasibility of Pharmacist-Driven Penicillin Allergy De-Labeling Pilot Program without Skin Testing or Oral Challenges. Pharmacy (Basel, Switzerland) 2021; 9(3).

Sneddon J, Cooper L, Ritchie N, et al. An algorithm for safe de-labelling of antibiotic allergy in adult hospital in-patients. Clinical and experimental allergy: journal of the British Society for Allergy and Clinical Immunology 2021.

Steenvoorden L, Bjoernestad EO, Kvesetmoen T-A, Gulsvik AK. De-labelling penicillin allergy in acutely hospitalized patients: a pilot study. BMC infectious diseases 2021; 21(1): 1083.

Stein A, MacBrayne C, Yang C, Sarin T, Hicks A, Searns J, et al. Clinical Pathway to Increase Rates of Penicillin Allergy De-labeling. Journal of allergy and clinical immunology. 2020;145(2):AB76.

Stone CA, Stollings JL, Lindsell CJ, Dear ML, Buie RB, Rice TW, et al. Risk-stratified Management to Remove Low-Risk Penicillin Allergy Labels in the ICU. American journal of respiratory and critical care medicine. 2020;201(12):1572–5.

Taremi M, Artau A, Foolad F, et al. Safety, Efficacy, and Clinical Impact of Penicillin Skin Testing in Immunocompromised Cancer Patients. Journal of Allergy and Clinical Immunology: In Practice 2019; 7(7): 2185.

Torney N, Tiberg M. Description of a pharmacist-managed penicillin allergy skin testing (PAST) service at a community teaching hospital. Open forum infectious diseases. 2018;5:S508.

Torney NP, Tiberg MD. Description of a pharmacist-managed/administered penicillin allergy skin testing service at a community hospital. American journal of health-system pharmacy: AJHP : official journal of the American Society of Health-System Pharmacists 2021; 78(12): 1066-73.

Trubiano JA, Smibert O, Douglas A, Devchand, Misha, Lambros B, et al. The Safety and Efficacy of an Oral Penicillin Challenge Program in Cancer Patients: A Multicenter Pilot Study. Open forum infectious diseases. 2018;5(12): ofy306.

Trubiano JA, Thursky K, Stewardson AJ, Urbancic K, Worth LJ, Sutherl, et al. The impact of an integrated antibiotic allergy testing program on antimicrobial stewardship: A multicentre evaluation. Journal of allergy and clinical immunology. 2017;139(2): AB377.

Trubiano, J. A., S. Vogrin, A. Copaescu, M. Nasra, A. Douglas, N. E. Holmes and K. Y. L. Chua (2022). "Direct oral penicillin challenge for penicillin allergy delabeling as a health services intervention: A multicenter cohort study." Allergy 77(3): 1038-1042.

Vyles D, Chiu A, Routes J, Castells M, Phillips EJ, Visotcky A, et al. Oral amoxicillin challenges in low-risk children during a pediatric emergency department visit. Journal of Allergy and Clinical Immunology: In Practice. 2020;8(3):1126.

Wall GC, Peters L, Leaders CB, Wille JA. Pharmacist-managed service providing penicillin allergy skin tests. American journal of health-system pharmacy: AJHP : official journal of the American Society of Health-System Pharmacists. 2004;61(12):1271–5.

Wong J, Timberlake K, Atkinson A, Science M. De-labeling of allergies to B-lactam antibiotics (De-LABeL) program: Development and pilot of an inpatient pediatric program. Open forum infectious diseases. 2018;5:S112.

Wrenn R, Sarubbi C, Kleris R, Drew R, Moehring R, Lugar P, et al. Antimicrobial stewarding with a unique pharmacist-managed penicillin skin testing (PST) service. Open forum infectious diseases. 2017;4:S270.

**Appendix 3: Studies excluded on full text**

**Wrong study design**

Abbo LM, Beekmann SE, Hooton TM, Johannsson B, Polgreen PM. Management of antimicrobial allergies by infectious diseases physicians. JAMA internal medicine. 2013;173(14):1376-8.

Ahmad H, Trytko U, i S. Decreasing Peri-Operative Non-Beta-Lactam Antibiotics with Screening Tool. Journal of Allergy and Clinical Immunology. 2020;145(2): AB56.

Alcenius G, Swihart T, Deighton K, Kak V, Huffman S, VanStee E, et al. Establishing a New Pharmacist-Managed Penicillin Allergy Testing Team. Pharmacotherapy.37(6): E63-E4.

Anstey KM, Choi L, Dawson D, Kleinhenz ME, Otani IM. Enabling antibiotic allergy evaluations and reintroduction of first-line antibiotics for patients with cystic fibrosis. Annals of allergy, asthma & immunology: official publication of the American College of Allergy, Asthma, & Immunology 2021; 127(4): 456-61.

Antoon JW, Grisso AG, Stone CA. Breaking the Mold: Safely Delabeling Penicillin Allergies in Hospitalized Children. Hospital pediatrics 2021; 11(5): e70-e2.

Atluri V, Marsland P, Pottinger P, Johnson LM, Jain R, Rampur L. Improving antibiotic prescribing in interventional radiology using clinical decision support tools to assess penicillin allergies. Open Forum Infectious Diseases 2020; 7: S54.

Barfield R, Meyers T, Bookstaver N, et al. Development and implementation of a pharmacistadministered penicillin allergy assessment and skin testing service in an outpatient internal medicine clinic. JACCP Journal of the American College of Clinical Pharmacy 2020; 3(8): 1537-8.

Berger RE, Singh HK, Loo AS, et al. Improving Antibiotic Stewardship for Inpatients with Reported Beta-Lactam Allergies and Limited Access to Penicillin Skin Testing. Joint Commission Journal on Quality and Patient Safety 2022.

Blumenthal KG, Wickner PG, Hurwitz S, Pricco N, Nee A, ra E, et al. Tackling inpatient penicillin allergies: Assessing tools for antimicrobial stewardship. The Journal of allergy and clinical immunology. 2017;140(1):154-61.e6.

Browne S, Neate K, Doherty S, et al. Antibiotic drug challenges in children; A safe and effective secondary care protocol. Clinical and Experimental Allergy 2021; 51(12): 1679.

Campbell S, Hauler G, Immler EL, Seiti S, Dandache P, Srinivas P. Pharmacist-led Penicillin Allergy Assessment in the Emergency Department Reduced Empiric Fluoroquinolone Use. Clinical infectious diseases : an official publication of the Infectious Diseases Society of America. 2020.

Cherk, Morris K, Collins CA. Partnering with general pediatricians to delabel penicillin allergies in children. Annals of Allergy, Asthma & Immunology. 2020;125(1):105-7.

Clark KE, Bri, E. M, Kapoor O, Pirasteh A. Impact of a Standardized Beta-Lactam Allergy Questionnaire on Aztreonam Use. Journal of pharmacy practice. 2019;32(4):399-403.

Collins CA, Choe D, Mochizuki D, Cannavino CR. Evaluating penicillin allergies in children using a standard EMR-based questionnaire. Annals of allergy, asthma & immunology : official publication of the American College of Allergy, Asthma, & Immunology. 2019;122(6):663-5.

Covington, Baldwin BJ, Warren E. Pharmacy-Led β-Lactam Allergy Interview (BLAI) Reduces Duration of Fluoroquinolones Within a Community Hospital. Annals of Pharmacotherapy. 2019;53(6):588-95.

del Real GA, Rose ME, Hammel J, Gordon SM, Arroliga ME. The penicillin skin test has a high negative predictive value and helps to modify the use of antibiotics in patiens with history of beta-lactam allergy. Journal of Allergy and Clinical Immunology. 2006;117(2):S224-S.

DiLoreto F, Alvarez K, Blackwell W, Khan D. Inpatient Penicillin Allergy Testing Via Clinical Decision Support and Proactive Antibiotic Changes Decreases Aztreonam Use. Journal of Allergy and Clinical Immunology. 2020;145(2):AB174.

Dufrene S, Meek J, Waibel K, Geist C, Acasio D, Nguyen M, et al. Workup and assessment of reported beta-lactam allergy in patients using a penicillin allergy testing protocol. Journal of the American Pharmacists Association. 2018;58(1).

Estep, Ferreira JA, Dupree LH, Aldridge PJ, Jankowski CA. Impact of an antimicrobial stewardship initiative to evaluate β-lactam allergy in patients ordered aztreonam. American Journal of Health-System Pharmacy. 2016;73.

Fabre, Salinas AB, Rosales L, Srinivasan A, Hicks L, Neuhauser M, et al. Implementation of a Nursing Algorithm for Penicillin Allergy Documentation in the Inpatient Setting...Sixth Decennial International Conference on Healthcare-Associated Infections. Infection Control & Hospital Epidemiology. 2020;41(S1).

Gadde J, Spence M, Wheeler B, Adkinson Jr NF. Clinical experience with penicillin skin testing in a large inner-city STD clinic. Journal of the American Medical Association. 1993;270(20):2456-63.

Garcia JFB, Aun MV, Motta AA, Castells M, Kalil J, Giavina-Bianchi P. Algorithm to guide re-exposure to penicillin in allergic pregnant women with syphilis: Efficacy and safety. The World Allergy Organization journal 2021; 14(6): 100549.

Generoso A, Muglia C, Mattappallil A, Khianey R, Capitle E, Wolff AH. Role of non-allergists in the removal of "penicillin-allergic" labels. Journal of Allergy and Clinical Immunology. 2018;141(2 Supplement 1):AB78.

Gjerset BG, Kwong F, Klaustermeyer WB. IMMEDIATE HYPERSENSITIVITY SKIN TESTING FOR PENICILLIN ALLERGY. Journal of Allergy and Clinical Immunology. 1992;89(1):365-.

Grillo JA, Ravin K, DeFelice ML. The effect of a penicillin allergy algorithm on perioperative antibiotic choice. Journal of Allergy and Clinical Immunology. 2016;137(2 SUPPL. 1):AB37.

Harrington N, Drews V, Laude J, Care J, Walsh D. Effect on beta-lactam usage following implementation of clinical pharmacy services to improve beta-lactam allergy history documentation. Open Forum Infectious Diseases. 2019;6(Supplement 2):S350.

Hawthorne A, Entrekin T, Kaletsch L, Jones KA. Pharmacist-led allergy evaluations reduced meropenem use in community hospital. JACCP Journal of the American College of Clinical Pharmacy. 2020;3(1):223.

Hernandez K, Maynard S, Chapin R, Mahoney MV, McCoy C, Lax T, et al. Grading the impact of a standardized b lactam antibiotic allergy assessment protocol for treatment decisions: An antimicrobial stewardship target. Open Forum Infectious Diseases. 2017;4(Supplement 1):S268.

Holmes AK, Bennett NT, Berry TP. Pharmacy driven assessment of appropriate antibiotic selection in patients with reported beta-lactam allergy. JACCP Journal of the American College of Clinical Pharmacy. 2019;2(5):509-14.

Jones BM, Hamlin A, Crosby J, Bland C. Clinical and economic outcome evaluation with penicillin skin testing as an antimicrobial stewardship initiative in a not-for-proft community health system. Open Forum Infectious Diseases. 2018;5(Supplement 1):S507.

Jones BM, Plauche E, Smith SE, Bland CM. Evaluating the utility of a penicillin allergy reconciliation program within an infectious diseases consult population in a community health system. Open Forum Infectious Diseases 2020; 7: S49-S50.

Joshi SR, Alvarez K, Wei W, Tarver SA, Vo K, Khan DA. Readmission rates following removal of penicillin allergy label after inpatient penicillin allergy testing. Journal of Allergy and Clinical Immunology. 2018;141(2 Supplement 1):AB289.

Joyce JG, Allen HI, Moylett EM. Analysis of referrals for paediatric beta-lactam allergy delabelling: Is there sufficient information to risk stratify, facilitating a 'see and treat' single dose oral challenge? Allergy: European Journal of Allergy and Clinical Immunology. 2020;75(SUPPL 109):609.

Katzen S, Woolbert A, Anderson S, Parker M, Anderson R, Slaton M, et al. The incidence and reliability of self reported penicillin allergy in a community hospital. Journal of Investigative Medicine. 2013;61(2):457.

Krey SC, Waise J, Skrupky LP. Confronting the Challenge of Beta-Lactam Allergies: A Quasi-Experimental Study Assessing Impact of Pharmacy-Led Interventions. Journal of Pharmacy Practice. 2019;32(2):139-46.

Kurtz K, Heyerly A, Bokhart G, Simpson W. Impact of a Pharmacist-Driven Penicillin Allergy Skin Testing Protocol on Antimicrobial Stewardship in a Tertiary Care Hospital. Hospital Pharmacy. 2021; 56(3): 136-8.

Kuruvilla M, Sexton ME, Wiley Z, Langfitt T, Lynde G, Wolf F. A streamlined approach to optimize perioperative antibiotic prophylaxis in the setting of penicillin allergy labels. Journal of Allergy and Clinical Immunology. 2020;145(2 Supplement):AB55.

Lambl B, Reyes-Dassum S, Oommen V, Dike O, Freeley M, Finocchiaro D, et al. Antibiotic challenge dose testing improves patient care and lowers costs in a community hospital: A 2-year prospective study. Open Forum Infectious Diseases. 2018;5(Supplement 1):S568.

Lebrun S, Lisung FG, Kambo V, Ilyas N. The allergy that wasn't: Inpatient penicillin testing; an important step for antibiotic stewardship. Journal of Hospital Medicine. 2018;13(4 Supplement 1).

Mann, Wu JY, Shah SS. Implementation of a Pharmacist-Driven Detailed Penicillin Allergy Interview. Annals of Pharmacotherapy. 2020;54(4):364-70.

Margallo, Smith EA, Marks G, Ben-Aderet M, Yang H, Madhusudhan M, et al. Optimizing utilization of beta-lactam surgical prophylaxis through implementation of a structured allergy assessment tool in a presurgical clinic. Infection Control & Hospital Epidemiology. 2019;40(12):1420-2.

Mistry A, Maskill D, Corps C, Savic S, Savic L. Feasibility and utility of testing for penicillin allergy status in patients attending for elective surgery. Clinical and Experimental Allergy. 2016;46(12):1626-

Murphy AW, Moylett EM, Allen HI, Vazquez-Ortiz M. DE-labelling beta-lactam allergy using telemedicine and single dose oral challenge in a low risk paediatric cohort: Outcome of DE-labelling process one year later. Allergy: European Journal of Allergy and Clinical Immunology 2020; 75: 101.

Nistico D, Passanisi S, Oppedisano EM, et al. Direct drug provocation test for the diagnosis of self-reported, mild and immediate drug hypersensitivity reaction in children and adolescents: our real-life experience. MINERVA PEDIATRICS 2021; 73(3): 209-14.

Pierce K, Jones B, Bl, C. Real-world pharmacoeconomic analysis of penicillin skin testing: Scratching the surface at a community hospital. JACCP Journal of the American College of Clinical Pharmacy. 2018;1(2):208.

Piotin A, Godet J, Trubiano JA, et al. Predictive factors of amoxicillin immediate hypersensitivity and validation of PEN-FAST clinical decision rule. Annals of allergy, asthma & immunology : official publication of the American College of Allergy, Asthma, & Immunology 2022; 128(1): 27-32.

Raja AS, Lindsell CJ, Bernstein JA, Codispoti CD, Moellman JJ. The use of penicillin skin testing to assess the prevalence of penicillin allergy in an emergency department setting. Annals of emergency medicine. 2009;54(1):72-7.

Phan, Allen B, Epps K, Alikhil M, Kamataris K, Tucker C. Initiative to reduce aztreonam use in patients with self-reported penicillin allergy: Effects on clinical outcomes and antibiotic prescribing patterns. American Journal of Health-System Pharmacy. 2018;75.

Redmond M. Leading the way in penicillin allergy evaluation. Annals of Allergy, Asthma and Immunology 2020; 125(6): 624-5.

Richardson R, Loprete J, Carr A, et al. De-labelling patients with antibiotic allergy in Sydney adult and paediatric hospitals-towards standardisation and improved patient outcomes. Allergy: European Journal of Allergy and Clinical Immunology 2020; 75: 97.

Shea K, Knight T, Jaso T, Bissett J, Moser MT, Hobbs ALV. The impact of a beta-lactam allergy assessment on aztreonam utilization within a healthcare system. Open Forum Infectious Diseases. 2018;5(Supplement 1):S507-S8.

Shweta FNU, Cano EJ, Virk A, et al. Development of a pathway for removal of inappropriate penicillin allergy labels in hospitalized patients. Open Forum Infectious Diseases 2020; 7: S86-S7.

Staicu ML, Brundige ML, Ramsey A, Brown J, Yamshchikov A, Peterson DR, et al. Implementation of a penicillin allergy screening tool to optimize aztreonam use. Am J Health Syst Pharm. 2016;73(5):298-306.

Swearingen, White C, Weidert S, Hinds M, Narro J, Guarascio A, et al. A multidimensional antimicrobial stewardship intervention targeting aztreonam use in patients with a reported penicillin allergy. International Journal of Clinical Pharmacy. 2016;38(2):213-7.

Turner NA, Wrenn R, Sarubbi C, et al. Evaluation of a Pharmacist-Led Penicillin Allergy Assessment Program and Allergy Delabeling in a Tertiary Care Hospital. JAMA network open 2021; 4(5): e219820.

Vaisman, McCready J, Hicks S, Powis J. Optimizing preoperative prophylaxis in patients with reported β-lactam allergy: a novel extension of antimicrobial stewardship. Journal of Antimicrobial Chemotherapy (JAC). 2017;72(9):2657-61.

Wang SK, Won S, Bandi S, Tobin M, Beshir M, Ravindran S, et al. Assessing outcomes of antimicrobial stewardship interventions along with a hospital-wide beta-lactam allergy guideline trough aztreonam use: A 5-year observation. Open Forum Infectious Diseases. 2018;5(Supplement 1):S508.

Watkins, Amaya L, Wolfe M, Schoen J, Stohs E, May S, et al. Use of a Beta-Lactam Graded Challenge Process at an Academic Medical Center...Sixth Decennial International Conference on Healthcare-Associated Infections. Infection Control & Hospital Epidemiology. 2020;41(S1).

73. Yoon K, Lee M, Patel R, Park Z. Successful Implementation of a Simple Algorithm to Manage Penicillin Allergy in an Acute Care Community Hospital. Annals of Pharmacotherapy. 2018;52(6):603-4.

P32: STANDARDISED TESTING AND COMMUNICATION INCREASE BETA-LACTAM ALLERGY DELABELLING EFFECTIVENESS: RESULTS OF 'DE-LABELLING PATIENTS WITH ANTIBIOTIC ALLERGY IN SYDNEY ADULT AND PAEDIATRIC HOSPITALS'...31st Annual Conference of the Australasian Society of Clinical Immunology and Allergy (ASCIA), 1-3 September, 2021 (Virtual). Internal Medicine Journal 2021; 51: 13-4.

ASSESSMENT OF THE VALIDITY OF THE BETA-LACTAM ANTIBIOTIC ALLERGY ASSESSMENT TOOL FOR USE IN THE RURAL CONTEXT, QLD...The Royal Australasian College of Physicians Congress, Transformation: Adapting for the Future, 29 April-14 May, 2021. Internal Medicine Journal 2021; 51: 16-.

**Wrong publication type**

Ackroyd JF. Skin test for penicillin hypersensitivity. Lancet (London, England). 1989;1(8633):335.

Arikoglu T, Kont AO, Demirhan A, Yuksek BC, Tokmeci N, Kuyucu S. Risk stratification in beta-lactam allergy. CURRENT TREATMENT OPTIONS IN ALLERGY 2021; 8(4): 285-97.

Brown M, Uzoma J, Vansice R, et al. Examining the impact of a penicillin allergy skin testing brochure on inpatient perceptions: A pre-post intervention study. The journal of allergy and clinical immunology In practice 2021; 9(4): 1736-7.e3.

Demain JG, Khan DA. Penicillin Testing in Infants. Journal of Allergy and Clinical Immunology: In Practice. 2020;8(5):1777.

Felix MMR, Kuschnir FC. Direct Oral Provocation Test Is Safe and Effective in Diagnosing Beta-Lactam Allergy in Low-Risk Children With Mild Cutaneous Reactions. Frontiers in Pharmacology. 2020;11.

Kufel WD, Justo JA, Bookstaver PB, Avery LM. Penicillin Allergy Assessment and Skin Testing in the Outpatient Setting. Pharmacy (Basel, Switzerland). 2019;7(3).

Traynor K. Iowa pharmacists fill allergy testing niche. American Journal of Health-System Pharmacy. 2014;71(11):893. Vaisman, McCready J, Powis J. Using In-depth History Screening as an Additional Method to Help Delabel Inappropriate β-Lactam Allergies...Blumenthal KG, Ryan EE, Li Y, Lee H, Kuhlen JL, Shenoy ES. The impact of a reported penicillin allergy on surgical site infection risk. Clin Infect Dis 2018; 66:329–36. Clinical Infectious Diseases. 2018;67(12):1959-60.

**Duplicate**

Allen H, Vazquez-Ortiz M, Murphy A, Moylett E. De-labelling beta-lactam allergy in children in an outpatient setting using a single dose protocol. Archives of Disease in Childhood. 2019;104(Supplement 3):A30-A1.

Bland, C., Jones B, Lin J. Pharmacist-directed penicillin skin testing as an antimicrobial stewardship initiative: Overview of results from a citywide approach. Pharmacotherapy. 2016;36(12): e289-e90.

Blumenthal K, Shenoy E, Varughese C, Hooper D, Banerji A. Outcomes after implementation of an inpatient antibiotic prescribing pathway for patients with penicillin or cephalosporin allergy. Journal of Allergy and Clinical Immunology. 2014;133(2): AB215.

Blumenthal KG, Wickner PG, Hurwitz S, Pricco N, Nee AE, Laskowski K, et al. Improving antibiotic choice in hospitalized medical patients reporting penicillin allergy. Journal of Allergy and Clinical Immunology. 2017;139(2 Supplement 1):AB181.

Campbell S, Hauler G, Immler E, Seiti S, Srinivas P, Dandache P. Pharmacist-driven penicillin allergy assessment in the emergency department-antimicrobial stewardship at the point of prescription. Open Forum Infectious Diseases. 2019;6(Supplement 2):S349-S50.

Chen JR, Tarver SA, Alvarez KS, Nguyen C, Khan DA. Reflexive penicillin allergy testing with in- hospital aztreonam use. Journal of Allergy and Clinical Immunology. 2017;139(2):AB30.

Chen JR, Tarver SA, Alvarez KS, Tran T, Khan DA. Beneficial outcomes of an inpatient penicillin allergy testing protocol. Journal of Allergy and Clinical Immunology. 2016;137(2):AB91.

Chen JR, Tarver SA, Alvarez KS, Tran T, Khan DA. A Proactive Approach to Penicillin Allergy Testing in Hospitalized Patients. The journal of allergy and clinical immunology In practice. 2017;5(3):686-93.

Du Plessis T, Holl, D., Jordan A, Walls G. Beta-lactam allergy assessment and management service: A pharmacist-led approach. Open Forum Infectious Diseases. 2016;3.

Kabchi B, Rimawi RH, Ashraf MS, Cook PP, Siraj DS, Gooch M, et al. The clinical benefit of penicillin skin testing in the medical intensive care unit. American Journal of Respiratory and Critical Care Medicine. 2013;187(MeetingAbstracts).

Grisanti KH, Stukus DR. Allergy testing in children with low-risk penicillin allergy symptoms. Pediatrics. 2018;142:S224.

Ham YY, Joshi S, Sukerman E, Lewis J, Tucker KJ, Yu D. Implementation of pharmacist-driven penicillin allergy evaluation and testing with a focus on bypassing penicillin skin testing at an academic medical center. Open Forum Infectious Diseases 2020; 7: S95.

Jones B, Crosby J, Bland C. Use of penicillin skin testing as an antimicrobial stewardship initiative: Clinical and economic evaluation at a community health system. Open Forum Infectious Diseases. 2017;4(Supplement 1):S491-S2.

Mann K, Shah S, Wu J. Impact of a pharmacist-driven detailed penicillin allergy interview. Open Forum Infectious Diseases. 2018;5(Supplement 1):S508-S9.

Rimmer J, Marwood J, Aguirrebarrena G, Kerr S, Welch SA. De-labelling self-reported penicillin allergy within the emergency department (ed) through the use of skin tests and oral drug provocation testing. Internal Medicine Journal. 2017;47(Supplement 5):11-2.

Sacco K, Cochran B, Tatari M, Alvarez CS, Sagheer TA, Berlioz B, et al. Inpatient penicillin allergy evaluation safely increases utilization of beta lactams. Clinical and Translational Allergy. 2018;8(Supplement 3).

Savic L, Kaura V, Gurr L, Toolan J, Glover N, Hopkins P, et al. Penicillin allergy De-labelling in the elective surgical population (PADLES). Clinical and Experimental Allergy. 2018;48(11):1525.

Searns JB, Stein A, MacBrayne C, et al. Single Dose Oral Amoxicillin Challenge is a Safe and Effective Strategy to Delabel Penicillin Allergies among Low Risk Hospitalized Children. Open Forum Infectious Diseases 2020; 7: S677.

Song Y, Nelson Z, Gens K. Effectiveness and feasibility of pharmacist-driven penicillin allergy de-labeling pilot program. Open Forum Infectious Diseases 2020; 7: S93.

Stone C, Lindsell C, Stollings J, Dear ML, Buie R, Rice T, et al. Risk-Stratified Management Offers a Safe Approach to Removing Low-Risk Penicillin Allergy Labels in the Intensive Care Unit. Journal of Allergy and Clinical Immunology. 2020;145(2 Supplement):AB94.

Stukus D, Chapparo J, Hehmeyer J, Hussain C, Lecerf K, Macias C, et al. Nursing Administered Questionnaire to Identify Pediatric Inpatients Eligible for Dose Graded Penicillin Challenge. Journal of Allergy and Clinical Immunology. 2020;145(2 Supplement):AB99.

Trubiano JA, Grayson ML, Phillips EJ, Stewardson AJ, Thursky KA, Slavin MA. Antibiotic allergy testing improves antibiotic appropriateness in patients with cancer. The Journal of antimicrobial chemotherapy. 2018;73(11):3209-11.

Winders HR, Justo JA, Bookstaver PB, Al-Hasan MN, Griffith NC, Mediwala KN. Regulatory approval, implementation, and brief assessment of a pharmacist- and pharmacy trainee-administered penicillin allergy assessment and skin testing program. JACCP Journal of the American College of Clinical Pharmacy 2020.

**Allergy specialists**

Arroliga ME, Radojicic C, Gordon SM, Popovich MJ, Bashour CA, Melton AL, et al. A prospective observational study of the effect of penicillin skin testing on antibiotic use in the intensive care unit. Infection control and hospital epidemiology. 2003;24(5):347-50.

Arroliga ME, Wagner W, Bobek MB, Hoffman-Hogg L, Gordon SM, Arroliga AC. A pilot study of penicillin skin testing in patients with a history of penicillin allergy admitted to a medical ICU. Chest. 2000;118(4):1106-8.

Artau A, Taremi M, Foolad F, Berlin S, White C, Mulanovich V, et al. Safety, efficacy, and clinical impact of penicillin allergy skin testing in immunocompromised cancer patients at a comprehensive cancer center. Open Forum Infectious Diseases. 2018;5(Supplement 1): S506.

Baynova K, Rivera G, Garnacho J, Lopez JM, Quiralte J, Cimbollek S, et al. The beta-lactam allergy in an intensive care unit: A prospective study. Allergy: European Journal of Allergy and Clinical Immunology. 2015;70(SUPPL. 101):19.

Blumenthal KG, Wolfson AR, Hsu JT, Shenoy ES, Li Y, Schwartz JM, et al. Outcomes of beta-lactam antibiotic test dose procedures for patients with reported beta-lactam allergy performed in a large US healthcare system. Journal of Allergy and Clinical Immunology. 2019;143(2 Supplement):AB25.

Brinez Giraldo T, Agullo Garcia A, Segura Arazuri N, Pano Pardo JR, Garces Sotillo M, Colas Sanz C. Impact of removing the beta-lactam allergy label in patients admitted with the need for the use of antibiotics. Allergy: European Journal of Allergy and Clinical Immunology. 2018;73(Supplement 105):209.

Cunha IM, Marques ML, Gouveia J, Gomes E. Allergy work-up on suspected beta-lactam allergy in children: Safety of oral challenge without skin testing in nonimmediate reactions. Allergy: European Journal of Allergy and Clinical Immunology. 2020;75(SUPPL 109):599-600.

Des Roches A, Chiron R, Paradis J, Blaquiere M, Paradis L. Penicillin allergy in a pediatric population: Usefulness of oral challenge. Journal of Allergy and Clinical Immunology. 2001;107(2):S4-S.

Ben Fadhel N, Aroua F, Chadli Z, et al. Betalactam hypersensitivity: The importance of delabelling in primary care. British journal of clinical pharmacology 2021; 87(12): 4619-24.

Jiang F, Jain R, Pottinger PS, Ayars AG, Altman MC. Automated allergy and infectious disease pharmacy consult to limit the use of aztreonam in patients with reported beta-lactam allergy. Journal of Allergy and Clinical Immunology. 2016;137(2 SUPPL. 1):AB196.

Keller L, Miller M, Huang M, Neville N, Morrisette T, Kalra A. Ruling out penicillin allergy at a university hospital: proof of concept. Journal of Allergy and Clinical Immunology. 2020;145(2 Supplement):AB98.

Knight K, Boardman A, Kane P, Schnitzer G, Fitzsimons R. Removing the burden of 'penicillin allergic' label in children through a nurse-led service. Clinical and Experimental Allergy. 2018;48(11):1545-6.

Kovacs C, Athans V, Lang D, Sobecks R, Rybicki L, Carlstrom K, et al. Penicillin allergy skin testing as an antibiotic stewardship intervention in hematopoietic cell transplant recipients. Open Forum Infectious Diseases. 2017;4(Supplement 1):S266-S7.

Levin AS, Banerji A, Li Y, Blumenthal KG. Risk stratification for outpatient penicillin allergy evaluations. Journal of Allergy and Clinical Immunology. 2018;141(2 Supplement 1):AB290.

Li, Shahabi-Sirjani A, Figtree M, Hoyle P, Fernando SL. Safety of direct drug provocation testing in adults with penicillin allergy and association with health and economic benefits. Annals of Allergy, Asthma & Immunology. 2019;123(5):468-75.

Loprete J, Richardson R, Kane A, Katelaris C, Post J, Wainstein B, et al. De-labelling patients with antibiotic allergy in Sydney adult and paediatric hospitals-towards standardisation and improved patient outcomes. Allergy: European Journal of Allergy and Clinical Immunology. 2020;75(SUPPL 109):97.

Lozo S, Wagner D, Goldberg R, Shah N, Silver RK, Solomonides T. Should Penicillin Allergy Be Confirmed During Pre-operative Testing? Obstetrics and Gynecology. 2019;133:223S-S.

Mendelson LM, Ressler C, Page J, Selcow J, Rosen J. ELECTIVE TESTING OF PENICILLIN ALLERGIC PATIENTS. Journal of Allergy and Clinical Immunology. 1987;79(1):200-.

Ramsey A, Holly AM, Mustafa SS, Staicu ML. Use of a Penicillin Allergy Screening Algorithm Incorporating Direct Challenges to Manage Penicillin Allergic Inpatients. Journal of Allergy and Clinical Immunology. 2019;143(2 Supplement):AB27.

Ramsey A, Holly AM, Staicu ML. Use of telemedicine for penicillin allergy de-labeling. Journal of Allergy and Clinical Immunology. 2018;141(2 Supplement 1):AB33.

Ramsey A, Mustafa SS, Holly AM, Staicu ML. Direct Challenges to Penicillin-Based Antibiotics in the Inpatient Setting. The journal of allergy and clinical immunology In practice. 2020;8(7):2294-301.

Ramsey A, Staicu ML. Use of a Penicillin Allergy Screening Algorithm and Penicillin Skin Testing for Transitioning Hospitalized Patients to First-Line Antibiotic Therapy. Journal of Allergy and Clinical Immunology: In Practice. 2018;6(4):1349-55.

Rothmeier JM, Markus P, Osmon D, Estes L, Hanssen A, Li JT. Pre-operative penicillin allergy testing in orthopedic patients with a history of penicillin allergy. Journal of Allergy and Clinical Immunology. 2002;109(1):S95-S.

Saleh M, Landry C, Do K, Khan I, Chagnon N, Chauret D. Evaluation of the impact of pharmacist-led penicillin allergy assessments on antibiotic utilization in a large community teaching hospital. Canadian Journal of Hospital Pharmacy. 2018;71(1):80.

Sloan A, Conlon N, Redenbaugh V, McCrea P, Barron A, Aoife M, et al. Test diagnose treat, a pilot study to delabel patients with suspected penicillin allergy. Clinical and Translational Allergy. 2018;8(Supplement 3).

Torney N, Tiberg M. Implementation of a pharmacist-managed penicillin skin testing (PST) service at a community teaching hospital. Open Forum Infectious Diseases. 2016;3(Supplement 1).

Vyles D, Adams J, Chiu A, Simpson P, Nimmer M, Brousseau DC. Allergy Testing in Children With Low-Risk Penicillin Allergy Symptoms. Pediatrics. 2017;140(2).

Wang LA, Patel K, Kuruvilla ME, Shih J. Direct amoxicillin challenge without preliminary skin testing for pediatric patients with penicillin allergy labels. Annals of allergy, asthma & immunology : official publication of the American College of Allergy, Asthma, & Immunology. 2020;125(2):226-8.

**Appendix 4 Definitions**

De-labelling was considered to be removal of an allergy record from a patient’s medical records. Skin testing was considered to be skin prick testing or intradermal injection of allergen. A direct oral challenge test was considered to be administration of either a single dose or graded dose of penicillin without prior skin testing. A graded challenge was considered to be initial administration of a low dose (e.g. 10%) of the therapeutic dose followed by administration of gradually increasing amounts until administration of a therapeutic dose was achieved. Direct de-labelling was considered to be removal of penicillin allergy records based on history alone. Successful de-label was defined as receipt of a systematic dose of a penicillin without hypersensitivity adverse drug reaction (ADR). Assessment of whether adverse drug reactions were due to hypersensitivity, or an intolerance was reported according to the study investigators. ADRs that were not thought to be due to hypersensitivity, or were reported as subjective, were excluded. If no formal judgement was reported then we assumed ADRs were hypersensitivity reactions in the analysis, as others have done.^6^

**Appendix 5: Critical Appraisal Results**

*Table: Case Series*

| **Citation** | **Q1** | **Q2** | **Q3** | **Q4** | **Q5** | **Q6** | **Q7** | **Q8** | **Q9** | **Q10** |
| --- | --- | --- | --- | --- | --- | --- | --- | --- | --- | --- |
| Adkinson NF, Thompson WL, Maddrey WC, Lichtenstein LM. 1971. | Y | Y | Y | N | N | N | U | Y | N | N/A |
| Allen HI, Vazquez-Ortiz M, Murphy AW, Moylett EM. 2020. | Y | Y | Y | U | Y | N | N | Y | Y | Y |
| Blackwell W, Khan D. 2020. | Y | U | U | N | U | N | N | Y | N | Y |
| Blumenthal KG, Li Y, Hsu JT, Wolfson AR, Berkowitz DN, Carballo VA, et al. 2019. | Y | Y | Y | U | Y | Y | N | Y | Y | Y |
| Chen JR, Tarver SA, Alvarez KS, Tran T, Khan DA. 2017. | Y | Y | Y | N | Y | Y | Y | Y | Y | Y |
| Devch, M., Kirkpatrick CMJ, Stevenson W, Garrett K, Perera D, et al. 2019. | Y | Y | Y | N | Y | Y | Y | Y | Y | Y |
| du Plessis T, Walls G, Jordan A, Holl, J. D. 2019. | Y | Y | Y | N | Y | Y | Y | Y | Y | Y |
| Eischens MR, Wolf LM, Dumkow LE, Anderson AM, Jameson AP, Br, et al. | Y | Y | U | U | U | N | Y | Y | Y | Y |
| Englert E, Weeks A. 2019. | Y | Y | Y | N | Y | Y | Y | Y | N | Y |
| Foolad F, Berlin S, White C, ice, Dishner E, Jiang Y, et al. 2019. | Y | Y | Y | U | Y | Y | Y | Y | Y | Y |
| Griffith NC, Justo JA, Winders HR, Al-Hasan MN, Mediwala KN, Bookstaver PB. 2020. | Y | Y | Y | N | Y | Y | Y | Y | Y | Y |
| Gugkaeva Z, Crago JS, Yasnogorodsky M. 2017. | Y | Y | Y | N | Y | N | N | Y | Y | N/A |
| Harmon S, Richardson T, Simons H, Monforte S, Fanning S, Harrington K. 2020. | Y | Y | Y | U | Y | N | N | Y | Y | Y |
| Harris AD, Sauberman L, Kabbash L, Greineder DK, Samore MH. 1999. | Y | Y | Y | Y | Y | N | N | Y | Y | Y |
| Heil EL, Bork JT, Schmalzle SA, Kleinberg M, Kewalramani A, Gilliam BL, et al. 2016. | Y | Y | Y | N | Y | N | Y | Y | Y | Y |
| Jones BM, Gamble K, Sizemore S, Bl, M. C. 2019. | Y | U | U | U | U | N | N | Y | N | N/A |
| Jones BM, Bl, M. C. 2017. | Y | Y | Y | U | U | N | N | Y | N | Y |
| Kleris R, Sarubbi C, Wrenn R, Anderson D, Lugar PL. 2018. | Y | Y | Y | U | U | N | N | Y | N | N/A |
| Kyi L, Heke E, McPhee S, Ojaimi S, Barnes S. 2018. | Y | Y | Y | U | Y | N | N | Y | Y | N/A |
| Lecerf K, Chaparro J, Hehmeyer J, Hussain C, Macias C, Vegh M, et al. 2020. | Y | Y | Y | N | U | N | N | N | Y | N/A |
| Leis, Palmay L, Ho G, Raybardhan S, Gill S, Kan T, et al. 2017. | Y | Y | Y | U | Y | Y | Y | Y | Y | Y |
| Lin L, Nagtegaal JE, Buijtels PCAM, Jong E. 2020. | Y | Y | Y | U | Y | Y | Y | Y | Y | Y |
| Livirya S, Pithie A, Chua I, Hamilton N, Doogue M, Isenman H. 2020. | Y | Y | Y | N | N | Y | U | Y | Y | Y |
| Lnumerables F, Fischer-Cartlidge E. | Y | Y | Y | U | Y | N | N | Y | Y | N/A |
| Lo SCR, Lacaria K, Mah A, Wong T, Mak R. 2020. | Y | Y | Y | U | U | N | N | Y | Y | N/A |
| Maguire M, Hayes BD, Fuh L, Elshaboury R, hi RG, Bor S, et al. 2020. | Y | Y | Y | U | Y | N | N | U | Y | Y |
| Marwood J, Aguirrebarrena G, Kerr S, Welch SA, Rimmer J. 2017. | Y | Y | Y | U | N | Y | N | Y | Y | Y |
| Murphy K, Scanlan B, Coghlan D. 2015. | Y | U | U | N | N | N | N | Y | Y | Y |
| Parker N, Choo HF, Ghodrat M. 2018. | N | U | U | U | U | N | N | N | Y | Y |
| Patel R, Saccone N, Stock K, Utley S, Bouknight D. 2019. | Y | U | U | U | U | N | N | U | N | N/A |
| Rahbani P. 2019. | N | U | U | U | U | N | N | N | N | Y |
| Rimawi RH, Cook PP, Gooch M, Kabchi B, Ashraf MS, Rimawi BH, et al. 2013. | Y | Y | Y | U | Y | Y | Y | Y | Y | Y |
| Rimawi RH, Mazer MA. 2014. | Y | Y | Y | N | N | Y | Y | Y | Y | Y |
| Savic L, Gurr L, Kaura V, Toolan J, oe JAT, Hopkins PM, et al. 2019. | Y | Y | Y | N | Y | N | N | Y | Y | Y |
| Sigona NS, Steele JM, Miller CD. 2016. | Y | Y | Y | N | N | N | N | Y | N | Y |
| Skibba N, Fischer J, Loecker B. | Y | Y | Y | U | U | N | N | N | N | N/A |
| Smibert O, Douglas A, Devch, M., Lambros B, Stevenson W, et al. 2018. | U | Y | Y | U | U | Y | Y | Y | Y | Y |
| Stone CA, Stollings JL, Lindsell CJ, Dear ML, Buie RB, Rice TW, et al. 2020. | Y | Y | Y | Y | Y | N | N | Y | Y | Y |
| Torney N, Tiberg M. 2018. | Y | Y | Y | U | U | N | N | Y | N | N/A |
| Trubiano JA, Smibert O, Douglas A, Devch, Misha, Lambros B, et al. 2018. | Y | Y | Y | U | U | Y | Y | Y | Y | Y |
| Wall GC, Peters L, Leaders CB, Wille JA. 2004. | Y | Y | Y | N | Y | N | N | Y | N | N/A |
| Wong J, Timberlake K, Atkinson A, Science M. 2018. | U | Y | Y | U | U | N | N | Y | Y | N/A |
| Wrenn R, Sarubbi C, Kleris R, Drew R, Moehring R, Lugar P, et al. 2017. | Y | Y | Y | U | Y | N | N | Y | N | N/A |
| Bauer ME, MacBrayne C, Stein A, Searns J, Hicks A, Sarin T, et al. 2021. | Y | Y | Y | Y | Y | N | N | Y | Y | Y |
| Gaudreau S, Bourque G, Cote K, Nutu C, Beauchesne M-F, Longpre A-A, et al. 2021. | Y | Y | Y | U | Y | Y | Y | N | Y | Y |
| Ham Y, Sukerman ES, Lewis JS, Tucker KJ, Yu DL, Joshi SR. 2021. | Y | Y | Y | N | Y | Y | Y | Y | N | Y |
| Harper HM, Sanchez M. 2021. | N | Y | Y | N | Y | N | N | N | N | Y |
| Sneddon J, Cooper L, Ritchie N, Steele C, Spears M, McEwen J, et al. 2021. | Y | Y | Y | N | Y | Y | N | Y | Y | Y |
| Song Y-C, Nelson ZJ, Wankum MA, Gens KD. 2021. | Y | Y | Y | N | Y | N | Y | Y | Y | Y |
| Steenvoorden L, Bjoernestad EO, Kvesetmoen T-A, Gulsvik AK. 2021. | Y | Y | Y | Y | Y | Y | N | Y | Y | Y |
| Torney NP, Tiberg MD. 2021. | Y | Y | Y | N | Y | Y | Y | Y | Y | Y |
| Louden NJ, Hansen LA, Rimal A, Norton LE. 2021. | Y | Y | Y | N | Y | N | N | Y | Y | Y |
| Mitchell AB, Ness RA, Bennett JG, Bowden JE, Elliott WV, Gillion AR, et al. 2021. | Y | Y | Y | Y | Y | Y | Y | Y | N | Y |
| Phung M, Vo T, Murfin B, Galbraith K, Barnes S, Coutsouvelis J. 2021. | N | Y | Y | U | Y | N | N | N | Y | Y |
| Rahbani P, Monroe-Duprey L. 2020. | N | U | Y | U | Y | N | N | N | Y | Y |
| Morjaria S, Inumerables F, Patel D, Cohen N, Seo S, Posthumus S, et al. 2021. | Y | Y | Y | Y | Y | Y | Y | Y | Y | Y |
| Taremi M, Artau A, Foolad F, Berlin S, White C, Jiang Y, et al. 2019. | Y | Y | Y | N | N | Y | Y | Y | Y | Y |
| % | 87.71 | 87.71 | 87.71 | 10.52 | 61.4 | 40.35 | 36.84 | 82.45 | 71.92 | 75.43 |
| Y, yes; N, no; U, unclear; NA, not applicable  Questions; 1. Were there clear criteria for inclusion in the case series? 2. Was the condition measured in a standard, reliable way for all participants included in the case series? 3. Were valid methods used for identification of the condition for all participants included in the case series? 4. Did the case series have consecutive inclusion of participants? 5. Did the case series have complete inclusion of participants? 6. Was there clear reporting of the demographics of the participants in the study? 7. Was there clear reporting of clinical information of the participants? 8. Were the outcomes or follow up results of cases clearly reported? 9. Was there clear reporting of the presenting site(s)/clinic(s) demographic information? 10. Was statistical analysis appropriate? | | | | | | | | | | |

*Table: Cohort Study*

| **Citation** | **Q1** | **Q2** | **Q3** | **Q4** | **Q5** | **Q6** | **Q7** | **Q8** | **Q9** | **Q10** | **Q11** |
| --- | --- | --- | --- | --- | --- | --- | --- | --- | --- | --- | --- |
| Chua KYL, Vogrin S, Bury S, Douglas A, Holmes NE, Tan N, et al. 2020. | Y | Y | Y | Y | Y | Y | Y | Y | Y | U | Y |
| Trubiano, J. A., S. Vogrin, A. Copaescu, M. Nasra, A. Douglas, N. E. Holmes and K. Y. L. Chua (2022). | Y | Y | Y | Y | Y | Y | Y | Y | Y | Y | Y |
| % | 100.0 | 100.0 | 100.0 | 100.0 | 100.0 | 100.0 | 100.0 | 100.0 | 100.0 | 50.0 | 100.0 |
| Y, yes; U, unclear.  1. Were the two groups similar and recruited from the same population? 2. Were the exposures measured similarly to assign people to both exposed and unexposed groups? 3. Was the exposure measured in a valid and reliable way? 4. Were confounding factors identified? 5. Were strategies to deal with confounding factors stated? 6. Were the groups/participants free of the outcome at the start of the study (or at the moment of exposure)? 7. Were the outcomes measured in a valid and reliable way? 8. Was the follow up time reported and sufficient to be long enough for outcomes to occur? 9. Was follow-up complete, and if not, were the reasons to loss to follow-up described and explored? 10. Were strategies to address incomplete follow-up utilized? 11. Was appropriate statistical analysis used? | | | | | | | | | | | |

*Table: Quasi-Experimental Study*

| Citation | Q1 | Q2 | Q3 | Q4 | Q5 | Q6 | Q7 | Q8 | Q9 |
| --- | --- | --- | --- | --- | --- | --- | --- | --- | --- |
| Blumenthal KG, Shenoy ES, Varughese C, Hurwitz S, Hooper D, Banerji A. 2015. | Y | Y | U | Y | N | U | Y | Y | Y |
| Chen JR, Tarver SA, Alvarez KS, Wei W, Khan DA. 2018. | Y | Y | Y | Y | N | Y | Y | Y | Y |
| Jones BM, Avramovski N, Concepcion AM, Crosby J, Bl, M C. 2019. | Y | Y | U | Y | N | U | Y | Y | Y |
| Nguyen CT, Sahbani O, Pisano J, Pursell K, Pettit NN. 2019. | Y | U | U | Y | U | U | Y | Y | U |
| Ravindran S, Beshir M, Wang S, i S, Hanson A, O’Driscoll T, et al. 2017. | Y | U | U | Y | U | U | Y | Y | U |
| Sacco KA, Cochran BP, Epps K, Parkulo M, Gonzalez-Estrada A. 2019. | Y | Y | U | Y | N | Y | Y | Y | Y |
| Shannon KT, Krop LC. 2016. | Y | Y | Y | Y | N | Y | Y | Y | Y |
| Stein A, MacBrayne C, Yang C, Sarin T, Hicks A, Searns J, et al. 2020. | Y | U | U | Y | Y | U | Y | Y | N/A |
| Trubiano JA, Thursky K, Stewardson AJ, Urbancic K, Worth LJ, Sutherl, et al. 2017. | Y | U | Y | Y | Y | Y | Y | Y | Y |
| % | 100.0 | 55.55 | 33.33 | 100.0 | 22.22 | 44.44 | 100.0 | 100.0 | 66.66 |
| Y, yes; N, no; U, unclear; NA, not applicable  1. Is it clear in the study what is the 'cause' and what is the 'effect' (i.e., there is no confusion about which variable comes first)? 2. Were the participants included in any comparisons similar? 3. Were the participants included in any comparisons receiving similar treatment/care, other than the exposure or intervention of interest? 4. Was there a control group? 5. Were there multiple measurements of the outcome both pre and post the intervention/exposure? 6. Was follow up complete and if not, were differences between groups in terms of their follow up adequately described and analyzed? 7. Were the outcomes of participants included in any comparisons measured in the same way? 8. Were outcomes measured in a reliable way? 9. Was appropriate statistical analysis used? | | | | | | | | | |

*Table: Randomized Controlled Trial*

| Citation | Q1 | Q2 | Q3 | Q4 | Q5 | Q6 | Q7 | Q8 | Q9 | Q10 | Q11 | Q12 | Q13 |
| --- | --- | --- | --- | --- | --- | --- | --- | --- | --- | --- | --- | --- | --- |
| Vyles D, Chiu A, Routes J, Castells M, Phillips EJ, Visotcky A, et al. 2020. | Y | N | Y | N | N | U | U | U | U | Y | Y | Y | Y |
| % | 100.0 | 0.0 | 100.0 | 0.0 | 0.0 | 0.0 | 0.0 | 0.0 | 0.0 | 100.0 | 100.0 | 100.0 | 100.0 |
| Y, yes; N, no; U, unclear;  1. Was true randomization used for assignment of participants to treatment groups? 2. Was allocation to treatment groups concealed? 3. Were treatment groups similar at the baseline? 4. Were participants blind to treatment assignment? 5. Were those delivering treatment blind to treatment assignment? 6. Were outcomes assessors blind to treatment assignment? 7. Were treatments groups treated identically other than the intervention of interest? 8. Was follow up complete and if not, were differences between groups in terms of their follow up adequately described and analyzed? 9. Were participants analysed in the groups to which they were randomized? 10. Were outcomes measured in the same way for treatment groups? 11. Were outcomes measured in a reliable way? 12. Was appropriate statistical analysis used? 13. Was the trial design appropriate, and any deviations from the standard RCT design (individual randomization, parallel groups) accounted for in the conduct and analysis of the trial? | | | | | | | | | | | | | |

**Appendix 6: Characteristics of Included Studies**

**Summary of Findings table A**

low risk patients (intolerance, delayed non-severe rash, unknown reaction), moderate risk patients (IgE), high risk patients (SCAR), DDL=direct de-label, DPT=direct provocation testing, ST/OC= skin testing followed by oral challenge, ST/-OC=skin testing without oral challenge, DIVC=direct intravenous challenge, ID=Infectious Diseases, OFS=Oral Facial Surgery, ICU=Intensive Care Unit, BMT=Bone Marrow Transplant, O&G= Obstetrics & gynaecology, PenA = penicillin allergy

| Reference | Study type | abstract, brief report, or full paper | Country, State, City | Setting / context | adult or paediatrics | Hospital specialties / wards | targeted all antibiotics, all beta-lactams, penicillin only | Allergy risk category | The penA de-label method used | Healthcare workers involved in the de-label process |
| --- | --- | --- | --- | --- | --- | --- | --- | --- | --- | --- |
| Adkinson NF, Thompson WL, Maddrey WC, Lichtenstein LM. 1971. | Case Series | full paper | USA, Maryland, Baltimore | inpatients | adults | medical service | penicillin | not clear | ST/OC | House Officers, medical students |
| Allen HI, Vazquez-Ortiz M, Murphy AW, Moylett EM. 2020. | Case Series | brief report | Ireland, Galway | outpatients | paediatrics | paediatrics | penicillin | low risk | DPT | primary care physician |
| Bauer ME, MacBrayne C, Stein A, Searns J, Hicks A, Sarin T, et al. 2021. | Case Series | full paper | USA, Colorado, Aurora | inpatients | paediatrics | paediatrics | penicillin | low risk | DDL, DPT | hospital medicine physicians |
| Blackwell W, Khan D. 2020. | Case Series | abstract | USA, Dallas, Texas | inpatients | adults | not reported | penicillin | not clear | ST/OC | pharmacists |
| Blumenthal KG, Li Y, Hsu JT, Wolfson AR, Berkowitz DN, Carballo VA, et al. 2019. | Case Series | full paper | USA, Massachusetts, Boston | inpatients | adults | Internal medicine, Emergency department, Surgery, oncology, Intensive care, Cardiology, Neurology, Obstetrics/Gynaecology, Paediatrics | beta-lactams | low risk | DPT | House staff, physician assistant, attending physician, nurse practitioner |
| Blumenthal KG, Shenoy ES, Varughese C, Hurwitz S, Hooper D, Banerji A. 2015. | Quasi-Experimental Study | full paper | USA, massachusetts, Boston | inpatients | adults | Internal medicine, oncology, cardiology, general surgery, oral maxillofacial surgery, urology | beta-lactams | low risk | DPT | House officers, nurse practitioners, attending hospitalist physicians and physician assistants in internal medicine, oncology, cardiology, general surgery, oral maxillofacial surgery, urology |
| Chen JR, Tarver SA, Alvarez KS, Tran T, Khan DA. 2017. | Case Series | full paper | USA, Texas, Dallas | inpatients | adults | Medical, Surgical, Obstetrics & gynaecology | penicillin | moderate risk | ST/OC | pharmacist |
| Chen JR, Tarver SA, Alvarez KS, Wei W, Khan DA. 2018. | Quasi-Experimental Study | full paper | USA, Texas, Dallas | inpatients | adults | medical surgical and ICU patients | penicillin | low, moderate risk | ST/OC | pharmacist |
| Chua KYL, Vogrin S, Bury S, Douglas A, Holmes NE, Tan N, et al. 2020. | Cohort Study | full paper | Australia, Victoria, Melbourne | inpatients | adults | Medical, surgical, cancer (haem/onc) | penicillin | low risk | DDL, DPT | Trained nursing, pharmacy, and/or medical staff & ward nurses (post challenge observation) |
| Devch, M., Kirkpatrick CMJ, Stevenson W, Garrett K, Perera D, et al. 2019. | Case Series | full paper | Australia, Victoria, Melbourne | inpatients | adults | not reported | penicillin | low risk | DDL, DPT, ST/OC | Infectious diseases physician, allergy nurse and an AMS pharmacist. |
| du Plessis T, Walls G, Jordan A, Holl, J. D. 2019. | Case Series | full paper | New Zealand, Auckland | inpatients | adults | not reported | penicillin | low risk | DDL, DPT | pharmacist, primary treating team |
| Eischens MR, Wolf LM, Dumkow LE, Anderson AM, Jameson AP, Br, et al. | Case Series | abstract | USA, Michigan, Grand Rapids | ED | adults | ED | beta-lactams | not reported | not reported | ED pharmacists & others (not specified) |
| Englert E, Weeks A. 2019. | Case Series | full paper | USA, Pennsylvania, Paoli | inpatients | adults | community hospital setting | penicillin | moderate risk | ST (-OC) | pharmacist |
| Foolad F, Berlin S, White C, ice, Dishner E, Jiang Y, et al. 2019. | Case Series | full paper | USA, Houston, Texas | inpatients | adults | haematology, oncology | penicillin | moderate risk | ST/OC | infectious diseases (ID) physician, ID fellow, ID pharmacist, advanced-practice providers. |
| Gaudreau S, Bourque G, Cote K, Nutu C, Beauchesne M-F, Longpre A-A, et al. 2021. | Quasi-Experimental Study | full paper | Canada, Sherbrooke, Quebec | inpatients | adults | not reported | penicillin | low, moderate risk | DDL, ST/OC | SPT pharmacists, IDT ID physicians, responsible clinicians (permission to proceed), pharm techs (make up testing solutions), allergist & allergy nurse delivered training for pharmacists |
| Griffith NC, Justo JA, Winders HR, Al-Hasan MN, Mediwala KN, Bookstaver PB. 2020 | Case Series | full paper | USA, South Carolina, Columbia | inpatients | adults | not reported | penicillin | not clear | DDL, ST/(+/-)OC | pharmacist, pharmacy trainee |
| Gugkaeva Z, Crago JS, Yasnogorodsky M. 2017 | Case Series | brief report | USA, Tennessee, Columbia | inpatients | adults | not reported | penicillin | moderate risk | ST/OC | pharmacists |
| Ham Y, Sukerman ES, Lewis JS, Tucker KJ, Yu DL, Joshi SR. 2021. | Case Series | full paper | USA, Portland, Oregon | inpatient | adults | not reported | penicillin | low, moderate & high risk | DDL, DPT, ST/OC | pharmacists |
| Harmon S, Richardson T, Simons H, Monforte S, Fanning S, Harrington K. 2020^6^ | Case Series | full paper | USA, Milwaukee, Wisconsin | inpatients | adults | General Medical patients | penicillin | moderate risk | ST/OC/IVC | pharmacist |
| Harper HM, Sanchez M. 2021. | Case Series | full paper | USA, Melbourne, Florida, | inpatient | adults | not reported | penicillin | moderate risk | DDL, DPT, ST, ST/OC | pharmacist did test, ward nurse monitored patient |
| Harris AD, Sauberman L, Kabbash L, Greineder DK, Samore MH. 1999. | Case Series | brief report | USA, Boston, Massachusetts | inpatients | adults | elective surgery (peri-op), general medical | penicillin | moderate risk | ST then required antibiotic | infectious disease physician, pharmacist, pre-op nurses |
| Heil EL, Bork JT, Schmalzle SA, Kleinberg M, Kewalramani A, Gilliam BL, et al. 2016. | Case Series | full paper | USA, Maryland, Baltimore | inpatients | adults | Medical/Surgical 68%, Intermediate Care Unit 9%, Intensive Care Unit 23% | penicillin | moderate risk | ST then required antibiotic | Infectious Disease Fellows, ID attending physician. |
| Jones BM, Avramovski N, Concepcion AM, Crosby J, Bl, M C. 2019. | Quasi-Experimental Study | full paper | USA, Savannah, Georgia | inpatients | adults | Intensive care unit, Progressive care unit, Medical/surgical floor, Oncology, Rehab, Emergency department, Labour and delivery, Outpatient surgery | penicillin | moderate risk | DDL, ST/ (+/-) OC | ID PharmD, ID physicians, nurse |
| Jones BM, Bl, M. C. 2017. | Case Series | brief report | USA, Georgia, Savannah | inpatients | adults | not reported | penicillin | moderate risk | ST/ (+/-) OC | nursing staff, clinical pharmacist, ward physicians |
| Jones BM, Gamble K, Sizemore S, Bl, M. C. 2019. | Case Series | abstract | USA, Georgia, Savannah | inpatients | adults | infectious diseases | penicillin | moderate risk | DDL, DPT, ST/OC | Advanced Pharmacy Practice Experience Students |
| Kleris R, Sarubbi C, Wrenn R, Anderson D, Lugar PL. 2018. | Case Series | abstract | USA, Durham, North Carolina. | inpatients | not clear | not reported | penicillin | not reported | ST/OC | pharmacists and infectious disease doctors |
| Kyi L, Heke E, McPhee S, Ojaimi S, Barnes S. 2018. | Case Series | abstract | Australia, Melbourne, Victoria, | inpatients | adults | General Medical patients | penicillin | low risk | DPT | pharmacists, a consultant Immunologist/Allergist/General Physician and medical registrar |
| Lecerf K, Chaparro J, Hehmeyer J, Hussain C, Macias C, Vegh M, et al. 2020. | Case Series | abstract | USA, Ohio, Columbus | inpatients | paediatrics | paeditaric Infectious Disease inpatient service | penicillin | low risk | DDL, DPT | nurse screening questionnaire, DPT HCW not reported |
| Leis, Palmay L, Ho G, Raybardhan S, Gill S, Kan T, et al. 2017. | Case Series | full paper | Canada, Toronto, Onatario, | inpatients | adults | inpatient and critical care | beta-lactams | moderate risk | ST/OC | Pharmacists ID physicians |
| Lin L, Nagtegaal JE, Buijtels PCAM, Jong E. 2020 | Case Series | full paper | The Netherlands, Amersfoort | inpatients | adults | not reported | penicillin | low risk | DPT | Treating physician |
| Livirya S, Pithie A, Chua I, Hamilton N, Doogue M, Isenman H. 2020. | Case Series | brief report | New Zealand, South Island, Christchurch | inpatients | adults | department of General Medicine | penicillin | low risk | DDL, DPT | Infectious Diseases fellow, medical student |
| Lnumerables F, Fischer-Cartlidge E. | Case Series | abstract | USA, New York, New York | inpatients & outpatients | adults | Bone Marrow Transplant patients, haematology patients | penicillin | not reported | ST/IVC | clinic nurse screening, Infectious Diseases doctor. |
| Lo SCR, Lacaria K, Mah A, Wong T, Mak R. 2020. | Case Series | abstract | Canada, British Columbia, Vancouver | outpatients | adults | pre-hematopoietic stem cell transplant patients | penicillin | not reported but defined by authors as "low risk" | DDL, ST/OC | pharmacist |
| Louden NJ, Hansen LA, Rimal A, Norton LE. 2021. | Case Series | full paper | USA, Minneapolis, Minnesota | inpatients | paediatrics | medical units, including critical care units | penicillin or cephalosporin | low risk | DDL | paediatric pharmacists |
| Maguire M, Hayes BD, Fuh L, Elshaboury R, hi RG, Bor S, et al. 2020. | Case Series | full paper | USA, Massachusetts, Boston | ED | adults | ED | beta-lactams | low risk | DPT | ED provider (medical doctors, nurse practitioners, and physician assistants), Pharmacist, ED nurse |
| Marwood J, Aguirrebarrena G, Kerr S, Welch SA, Rimmer J. 2017. | Case Series | full paper | Australia, New South Wales, Sydney, | ED | adults | ED | penicillin | moderate risk | ST/OC | Nurses, skin testing by emergency physicians |
| Mitchell AB, Ness RA, Bennett JG, Bowden JE, Elliott WV, Gillion AR, et al. 2021. | Case Series | full paper | USA, Memphis, Tennessee | inpatients | adults | inpatient | beta-lactams | low risk | DDL | inpatient clinical pharmacists |
| Morjaria S, Inumerables F, Patel D, Cohen N, Seo S, Posthumus S, et al. 2021. | Case Series | full paper | USA, New York, NY | outpatients | adults | haematology oncology | penicillin | moderate risk | ST/IVC | clinic nurse screened and risk assessed allergy history, if eligible referred to ID service, pharmacy prepared the ST reagents. |
| Murphy K, Scanlan B, Coghlan D. 2015. | Case Series | brief report | Ireland, Dublin, | inpatients & outpatients | paediatrics | ED, inpatient wards and out-patient clinics | penicillin | not reported but defined by authors as "low risk" | DDL, DPT | paediatric doctor, paediatric nurse |
| Trubiano, J. A., S. Vogrin, A. Copaescu, M. Nasra, A. Douglas, N. E. Holmes and K. Y. L. Chua (2022).. | Cohort Study | brief report | Australia, Melbourne | inpatient | adults | medical, surgical, haem/onc, ICU (included 35% immunocompromised) | penicillin | low risk | DPT | AMS-Allergy team |
| Nguyen CT, Sahbani O, Pisano J, Pursell K, Pettit NN. 2019. | Quasi-Experimental Study | abstract | USA, Illinois, Chicago | inpatients | adults | medicine | beta-lactams | low risk | DDL | pharmacists |
| Parker N, Choo HF, Ghodrat M. 2018. | Case Series | abstract | USA, Wyoming, Cheyenne | inpatients | adults | inpatients | penicillin | not reported | ST (unclear if received OC) | pharmacist |
| Patel R, Saccone N, Stock K, Utley S, Bouknight D. 2019. | Case Series | abstract | USA, South Carolina, Charleston | inpatients | adults | not reported | penicillin | not reported | DDL, DPT | Pharmacist, infectious disease physician |
| Phung M, Vo T, Murfin B, Galbraith K, Barnes S, Coutsouvelis J. 2021. | Case Series | abstract | Australia, Melbourne, Victoria | inpatients | adults | ICU | penicillin | low risk | DPT | clinical pharmacists |
| Rahbani P, Monroe-Duprey L. 2020. | Case Series | abstract | USA, Mclean, Virginia | outpatients | paediatrics | outpatient pre-surgical clinic | penicillin | not reported | ST (unclear if received OC) | pharmacist risk stratified patient and read ST result. Nurse administered ST |
| Rahbani P. 2019. | Case Series | abstract | USA, Virginia, Arlington | inpatients | not reported | not reported | antibiotic | not reported | DDL | pharmacist |
| Ravindran S, Beshir M, Wang S, i S, Hanson A, O’Driscoll T, et al. 2017. | Quasi-Experimental Study | abstract | USA, Illinois, Chicago | inpatients | not clear | not reported | beta-lactams | not reported | ST/OC | physicians |
| Rimawi RH, Cook PP, Gooch M, Kabchi B, Ashraf MS, Rimawi BH, et al. 2013. | Case Series | brief report | USA, North Carolina, Greenville | inpatients | adults | medical, surgical, labour, and delivery wards; intensive care units; and emergency department | penicillin | moderate risk | ST/OC | infectious diseases fellow, |
| Rimawi RH, Mazer MA. 2014. | Case Series | brief report | USA, North Carolina, Greenville | inpatients | adults | critical care | penicillin | moderate risk | ST/OC | physicians, medical students, nurses, pharmacists |
| Sacco KA, Cochran BP, Epps K, Parkulo M, Gonzalez-Estrada A. 2019. | Quasi-Experimental Study | full paper | USA, Florida, Jacksonville | inpatients | adults | general internal medicine | penicillin | low risk | DDL, DPT | ward doctors |
| Savic L, Gurr L, Kaura V, Toolan J, oe JAT, Hopkins PM, et al. 2019. | Case Series | full paper | UK, Leeds | peri-operative | adults | peri-operative | penicillin | low risk | DPT | pre-op nurses, anaesthetist |
| Shannon KT, Krop LC. 2016. | Quasi-Experimental Study | full paper | USA, Florida, Clearwater | inpatients | adults | not reported | beta-lactams | moderate risk | ST (-OC) | pharmacist, infectious disease physician, infection control practitioner. |
| Sigona NS, Steele JM, Miller CD. 2016. | Case Series | brief report | USA, New York, Syracuse | inpatients | adults | not reported | beta-lactams | low risk | DDL | Pharmacy resident, clinical pharmacist |
| Skibba N, Fischer J, Loecker B. | Case Series | abstract | USA, South Dakota, Sioux Falls | inpatients | adults | not reported | penicillin | not reported | ST (unclear if received OC) | pharmacist |
| Smibert O, Douglas A, Devch, M., Lambros B, Stevenson W, et al. 2018. | Case Series | abstract | Australia, Victoria, Melbourne | inpatients | adults | 50% of patients had cancer (haem/onc) | penicillin | low risk | DPT | ID and antimicrobial stewardship (AMS) services |
| Sneddon J, Cooper L, Ritchie N, Steele C, Spears M, McEwen J, et al. 2021. | Case Series | brief report | UK, Scotland | inpatient, pre-op | adults | medical wards (Infectious Diseases, Respiratory, Medical Admissions) or attending pre-operative assessment clinics. | penicillin | low risk | DPT | infectious disease teams (consultants, registrars and nurses). |
| Song Y-C, Nelson ZJ, Wankum MA, Gens KD. 2021. | Case Series | full paper | USA, Minneapolis, Minnesota | inpatients | adults | medical/surgical units and labour/delivery and high-risk pregnancy units | penicillin | low risk | DDL | Infectious Diseases pharmacy resident |
| Steenvoorden L, Bjoernestad EO, Kvesetmoen T-A, Gulsvik AK. 2021. | Case Series | full paper | Norway, Oslo | inpatients | adults | internal medicine | penicillin | low risk | DPT | doctors |
| Stein A, MacBrayne C, Yang C, Sarin T, Hicks A, Searns J, et al. 2020. | Quasi-Experimental Study | abstract | USA, Colorado, Denver | inpatients | paediatrics | paediatric inpatient | penicillin | low risk | DPT | physicians |
| Stone CA, Stollings JL, Lindsell CJ, Dear ML, Buie RB, Rice TW, et al. 2020. | Case Series | brief report | USA, Tennessee, Nashville | inpatients | adults | medical intensive care unit | penicillin | not reported but defined by authors as "low risk" | DPT | pharmacist, physician |
| Taremi M, Artau A, Foolad F, Berlin S, White C, Jiang Y, et al. 2019. | Case Series | full paper | USA, Texas, Houston | inpatients | adults | leukaemia and genitourinary medical oncology | penicillin | moderate risk | DDL, ST/OC | Screening by ID pharmacist. PST was performed by the ID physician, ID fellow, or ID advanced practice providers using standard methodology described elsewhere |
| Torney N, Tiberg M. 2018. | Case Series | abstract | USA, Michigan, Traverse City, | inpatients | adults | infectious diseases | penicillin | moderate risk | ST (unclear if received OC) | ID physician, ID pharmacist, PGY2 ID pharmacy resident, PGY1 pharmacy practice residents |
| Torney NP, Tiberg MD. 2021. | Case Series | full paper | USA, Michigan, Traverse City, | inpatients | adults | general medical, ICU, | penicillin | moderate risk | ST/OC | postgraduate year 2 (PGY2) infectious diseases (ID) pharmacy resident and ID pharmacist. |
| Trubiano JA, Smibert O, Douglas A, Devch, Misha, Lambros B, et al. 2018 | Case Series | full paper | Australia, Victoria, Melbourne | inpatients & outpatients | adults | haematology, oncology, | penicillin | low risk | DPT | antibiotic allergy nurse and infectious diseases physician |
| Trubiano JA, Thursky K, Stewardson AJ, Urbancic K, Worth LJ, Sutherl, et al. 2017. | Quasi-Experimental Study | full paper | Australia, Victoria, Melbourne | inpatients | adults | Infectious diseases, haematology, oncology, transplant services | all antibiotics | low, moderate & high risk | ST/OC, DPT, PT | ID physicians and allergy nurses |
| Vyles D, Chiu A, Routes J, Castells M, Phillips EJ, Visotcky A, et al. 2020. | RCT | brief report | USA, Wisconsin, Milwaukee, | ED | paediatrics | ED | penicillin | not reported but defined by authors as "low risk" | DPT | paediatric ED providers |
| Wall GC, Peters L, Leaders CB, Wille JA. 2004. | Case Series | full paper | USA, Iowa, Des Moines | inpatients | adults | not reported | beta-lactams | moderate risk | DDL, ST/OC | pharmacist, ward doctor, ward nurse, |
| Wong J, Timberlake K, Atkinson A, Science M. 2018. | Case Series | abstract | Canada, Ontario, Toronto | inpatients | paediatrics | paediatrics | beta-lactams | not reported | DPT | paediatricians |
| Wrenn R, Sarubbi C, Kleris R, Drew R, Moehring R, Lugar P, et al. 2017. | Case Series | abstract | USA, North Carolina, Durham | inpatients | adults | not reported | penicillin | not clear | ST (unclear if received OC) | pharmacists |

**Summary of Findings table B**

| Reference | summary of interventions | Training details | Any measured antimicrobial stewardship impact- | Any measured healthcare system impact |
| --- | --- | --- | --- | --- |
| Adkinson NF, Thompson WL, Maddrey WC, Lichtenstein LM. 1971. | education | no | not reported | not reported |
| Allen HI, Vazquez-Ortiz M, Murphy AW, Moylett EM. 2020. | expert available, patient pathway |  |  |  |
| Bauer ME, MacBrayne C, Stein A, Searns J, Hicks A, Sarin T, et al. 2021. | clinical care pathway, educational intervention, approval sought from Institutional clinical care guideline and measures review committee and our pharmacy and therapeutics committee, quality improvement team involvement, patient education handout, an electronic medical record (EMR) order set, EHR patient flag, stakeholder engagement, staff education, financial and gift incentive for staff if de-label targets met | no "Because the culture at our institution and many others is to strictly avoid medications to which a patient is reportedly allergic, there was a significant educational component to our quality improvement project." |  |  |
| Blackwell W, Khan D. 2020. | education | no |  |  |
| Blumenthal KG, Li Y, Hsu JT, Wolfson AR, Berkowitz DN, Carballo VA, et al. 2019. | guideline, EHR CDS, expert available | no |  |  |
| Blumenthal KG, Shenoy ES, Varughese C, Hurwitz S, Hooper D, Banerji A. 2015. | Guideline, education, posters, pathway | Targeted educational presentations were delivered to 15 different groups of general inpatient providers throughout the hospital. The presentations introduced the clinical guideline, showed providers how navigate to the guideline electronically, and communicated antibiotic stewardship goals and key concepts about penicillin allergy using interactive clinical vignettes. Instruction on taking an allergy history was provided, with emphasis on improved characterization of rashes (urticaria vs. delayed maculopapular rash vs. severe cutaneous adverse reactions). After each presentation, laminated cards with figures from the clinical guideline were distributed to staff and posted in hospital workrooms. After the initial educational roll-out, additional educational sessions included training AI and ID fellows, paediatrics residents, and a case-based discussion of penicillin allergy with internal medicine residents. Because of house staff turnover, additional annual education and training have been necessary. | Guideline-directed test doses resulted in significantly more patients on penicillins (19% vs 2%, p<0.001) and cephalosporins in the 1st (10% vs 0.6 %, p<0.001), 3rd (30% vs 5%, p<0.001), and 4th (32% vs 7%, p<0.001) generations. Of patients switched to a penicillin (n=33), 79% had primary penicillin allergy. After the test dose, significantly fewer patients were on vancomycin (37% vs 68%%, p<0.001), aminoglycosides (1% vs 6%, p=0.004), aztreonam (0.6% vs 12%, p<0.001) and fluoroquinolones (3% vs 15%, p<0.001). | none reported |
| Chen JR, Tarver SA, Alvarez KS, Tran T, Khan DA. 2017. | Education, EHR CDS, governance, expert available, outreach | no |  |  |
| Chen JR, Tarver SA, Alvarez KS, Wei W, Khan DA. 2018. | Clinical decision support (CDS) integrated in electronic medical records (EMRs), education, access to allergy expert (allergy fellow), protocol | no | 58% increase in penicillin exposure (P = .046). | took 2 hours per patient |
| Chua KYL, Vogrin S, Bury S, Douglas A, Holmes NE, Tan N, et al. 2020. | education, pathway, guideline | no | No difference in the duration of intravenous antibiotic delivery. In the de-labelled group, there was increased utilization post testing (index admission and 90 days) of penicillins (P < .001) and a reduction in cephalosporin (unrestricted and restricted), clindamycin, and restricted antibiotics usage (P < .01) | No difference in LOS, readmission rate, or mortality. Cost if implemented as standard of care: direct de-label, no cost, direct oral challenge ,35.18 AUD/patient |
| Devch, M., Kirkpatrick CMJ, Stevenson W, Garrett K, Perera D, et al. 2019. | Health informatic systems, triage, pathway |  | 106 patients that met the inclusion criteria, 45 patients (42.5%) were prescribed a restricted antibiotic pre-ward round compared with 19 patients (17.9%) 24 h post-ward round (P"0.0002). |  |
| du Plessis T, Walls G, Jordan A, Holl, J. D. 2019. | Education, patient education and patient material, patient pathway | no | The use of penicillins and cephalosporins increased by 29% in the de-labelled group (P<0.001 and P<0.02, respectively), whereas the use of other agents decreased commensurately. Penicillin antibiotics were prescribed for 75 of 270 courses (28%) preintervention compared with 118 of 189 courses (62%) postintervention in this group (P<0.0001). | Inpatient costs: 1.6 times greater cost per antibiotic per day for patients labelled as penicillin allergic. Outpatient costs: The antibiotic cost per day per patient for confirmed allergic patients was 2.5 times greater than for de-labelled patients. LOS Patients who were inaccurately labelled as allergic to penicillin had a median length of stay of 6 days (IQR 2–8 days), compared with patients who were confirmed allergic (median 9 days, IQR3–13.5 days, P<0.0015). |
| Eischens MR, Wolf LM, Dumkow LE, Anderson AM, Jameson AP, Br, et al. | Coordination of care and management of care processes - Teams - Creating and delivering care through a multidisciplinary team of healthcare workers. |  | Rates of first-line treatment also increased from 2010 to 2014 and 2016 (2.9%, 46.6%, 55.8%, p < 0.001) |  |
| Englert E, Weeks A. 2019. | Governance, education, health informatic systems. Patient information/education. Provider referral | no | A decrease in the use of fluoroquinolones and vancomycin and an increase in narrow spectrum penicillin-based antibiotics and first- and second-generation cephalosporins. | Antibiotic acquisition cost reduction in several patients and a long IV antibiotic drug avoided in one patient. |
| Foolad F, Berlin S, White C, ice, Dishner E, Jiang Y, et al. 2019. | Patient information, communication with healthcare providers, team |  | Increased beta-lactam use, including penicillin, and decreased aztreonam use | $1914.93 USD saved per patient switched from aztreonam to another beta-lactam. |
| Gaudreau S, Bourque G, Cote K, Nutu C, Beauchesne M-F, Longpre A-A, et al. 2021. | health informatics to identify patients. training of pharmacists & ID physician to perform the skin-prick test a. decision algorithm. sticker was affixed to the patient’s hospital card to advise the removal of penicillin allergy from the patient’s medical record |  |  | The pharmacist intervention involved an estimated workload of 0.15 FTEs (Interquartile range (IR): 0.12-0.25) for a hospital pharmacist working 36.25 h/week) Pharmacy technician time was estimated to be 30 min/week for the preparation of multiple syringes of the reagents, and ID physicians spent 15 minutes per patient for the intradermal injection, when completed. 15 patients assessed per week and for 2 patients a week tested). Of the 26 patients for whom allergy was ruled out, 16 suggestions were made to change the antibiotic therapy (61.5%); 13 were accepted by the treating physician (81.3%). Of the 13 suggestions to change the antibiotic therapy that were accepted, 9 led to the use of an antibiotic with a narrower spectrum of activity (69.2%). |
| Griffith NC, Justo JA, Winders HR, Al-Hasan MN, Mediwala KN, Bookstaver PB. 2020. | Education, Regulatory approval, provider placed a PAAST consult in the EHR, EHR facilitated referral, “Negative PCN Allergy Skin Test” EHR placeholder, patient education, patient education material, communication with other healthcare providers. | Initial training on allergy evaluation and skin testing was provided on-site by the manufacturer's representative of the penicillin skin testing product, during which the stewardship pharmacists were certified through a “train-the-trainer” program so that they could subsequently provide training to new team members, new residents, and pharmacy students. All pharmacy personnel, including students, were required to conduct a minimum of two skin tests within a six-month period to maintain competency, given the lack of a standardized training program at the time of the study. Trained personnel were also required to maintain basic life support certification. | Vancomycin use significantly decreased after a negative skin test from 16/36 (44%) patients pre-skin test to 6/36 (17%) patients post skin test (P = .01). Use of penicillins significantly increased from 0/36 (0%) patients pre-skin test to 14/36 (39%) patients post-skin test (P < .0001). |  |
| Gugkaeva Z, Crago JS, Yasnogorodsky M. 2017 | Education, governance, outreach & triage | Training programme (didactic and hands-on). The didactic component of the training includes a review of mechanisms of drug hypersensitivity reactions, current AAAAI guidelines for allergy skin testing and the management of anaphylaxis, online videos regarding testing and instructions for appropriately reading and documenting test results. The hands-on training involves practicing the testing procedure on healthy volunteers. All newly trained pharmacists perform at least three consultations under the supervision of an infectious diseases’ pharmacist. | 40 of 51 patients (78%) who tested negative were switched to a narrower-spectrum beta-lactam. Nineteen of 51 patients (37%) were switched to oral beta-lactams and were discharged within 24 hours of testing, indicating that penicillin allergy testing allows us to expedite discharges by expanding our choices of antibiotics. | If it is assumed that the length of stay for those 19 patients was reduced by 1 day, then the estimated savings was ~$30 000/year after subtracting costs associated with the penicillin allergy testing. Savings are estimated based on the average cost of a hospital bed per 24 hours. Any unintended harm associated with the de-label process - not reported |
| Ham Y, Sukerman ES, Lewis JS, Tucker KJ, Yu DL, Joshi SR. 2021. | electronic medical record (EMR) patient identifiers, referral Verbal consent, Pharmacist training, protocols for penicillin allergy evaluation and testing, the allergy physician was also available for discussion of complicated cases. algorithm. patient information inc. advice to communicate testing outcome with other HCPs, the results of the evaluation and testing were documented as a progress note, the allergy section was updated to reflect the results of the test. |  | 54% changed antibiotic treatment as a result of PADL with 50% changing to penicillin and 4% to a cephalosporin. Fluroquinolone, vancomycin, clindamycin and macrolide use reduced. |  |
| Harmon S, Richardson T, Simons H, Monforte S, Fanning S, Harrington K. 2020 | Education, EHR alert (reminder), governance, protocol | no | Pharmacist recommendation to change therapy based on PST results was accepted in 13 of 15 patients where recommendations were made | Cost savings in antimicrobial therapy alone for patients who received PST was US $74.75 per day (although cost of testing is US $175.02) |
| Harper HM, Sanchez M. 2021. | Pharmacists received training on allergy assessment and skin testing. Patient education. The pharmacist would then remove the penicillin allergy from the patient’s EHR, complete a progress note, and contact the patient’s outpatient pharmacy with updated allergy information. |  | 17 (61%) were transitioned to preferred beta-lactam antibiotics | No readmissions due to infectious causes within 30 days were observed, Aztreonam was discontinued in 6 patients, with an avoidance of 89 doses. An average of 4 days of aztreonam were avoided for each of these patients resulting in a total cost avoidance of $3,831.80. |
| Harris AD, Sauberman L, Kabbash L, Greineder DK, Samore MH. 1999. | Health informatic systems |  | The recommendations to change antibiotics were followed in 36 (95%) of the 44 patients. For the 23 patients in the therapeutic antibiotic group whose antibiotics, antibiotic regimens ordered before skin testing included vancomycin in 11 patients, fluoroquinolones (ciprofloxacin or ofloxacin) in 12 patients, and clindamycin in 10 patients. After skin testing, 15 patients in the therapeutic antibiotic group received cephalosporins, 7 received a penicillin, and 1 received both a penicillin and a cephalosporin. Vancomycin (n = 0, P ,0.001), clindamycin (n=0, P ,0.01), and the fluoroquinolones (n = 6, P ,0.02) were used significantly less often after skin testing than they would have been in 36 (95%) of the 44 patients. Of the 13 patients in the prophylactic antibiotic group who were skin test negative, 11 were scheduled to receive vancomycin as preoperative antibiotic prophylaxis and 2 were scheduled to receive ciprofloxacin and gentamicin. All 13 patients instead received cefazolin. | Yearly projected antibiotic savings were about $12,400 |
| Heil EL, Bork JT, Schmalzle SA, Kleinberg M, Kewalramani A, Gilliam BL, et al. 2016. | Education, communication with other healthcare providers. | Education and training on PST, including practical preparation and a didactic lecture. Initial training was given onsite by representatives from ALK-Abello with subsequent trainings and refresher courses led by “super-users”. Training included hands-on demonstration using actual testing supplies and volunteers with reported penicillin allergy from department staff. Super-users were three ID faculty who had received training from ALK-Abello and had the most experience with testing. Competency was assessed via a checklist that was evaluated by one of the super-users. All providers also had access to web-based videos, a slide presentation, and suggested literature. Individualized follow-up training sessions for the fellows before their scheduled penicillin allergy service month were also provided. | Aztreonam use decreased from a mean ± standard deviations of 3.4 ± 0.9 DOT/1000 PD to 1.9 ± 0.9DOT/1000 PD (P = .0015) after PST implementation. Overall, 63% of patients received a narrower spectrum antibiotic, 80% received more effective therapy, and 61% received more cost-effective therapy because of PST testing. | aztreonam savings $26 000 per year. Any unintended harm associated with the de-label process - No patient experienced any serious adverse effects related to non-aztreonam β-lactam antibiotics; 3 patients had a delayed mild rash after switch to a β-lactam (nafcillin, ertapenem, and cefepime, respectively). |
| Jones BM, Avramovski N, Concepcion AM, Crosby J, Bl, M C. 2019. | Patient education, patient material, EHR note (placeholder) | no | Seventy out of the 98 patients who tested negative for PST (71%) had changes directly made to their antimicrobial regimens immediately after PST. The most common change was from carbapenems to penicillins (34/70). For the primary outcome, beta-lactam DOT for the PST group were 666 out of 1094 (60.88%, with 34.82% being a penicillin specifically). Beta-lactam DOT for the control group consisted of 386 out of 984 (39.64%, with 6.4% being a penicillin specifically). The chi-square test of homogeneity for beta-lactam DOT between the 2 groups was significant (P < .00001) after PST (n = 70) | Specifically, for the patients who received antimicrobial changes after PST (n = 70), the cost savings increased to an average of $556.91 per patient. de-label process takes approximately 45 to 60 minutes to complete. charge performing PST $140 |
| Jones BM, Bl, M. C. 2017. | Patient education, education, Health informatics | no | Conversion from a carbapenem to a penicillin or a cephalosporin occurred in 12 patients. Other changes included conversions from vancomycin, aztreonam, or an aminoglycoside to a penicillin or a cephalosporin. Three patients who were not receiving an antimicrobial prior to testing were initiated on a penicillin or a cephalosporin after the test. | The average antimicrobial acquisition cost savings per patient was approximately $314.75; pharmacist can spend as much as 1.5 hours involved in the process, nurses can spend close to an hour administering the test. |
| Jones BM, Gamble K, Sizemore S, Bl, M. C. 2019. | Education | no |  |  |
| Kleris R, Sarubbi C, Wrenn R, Anderson D, Lugar PL. 2018. | multidisciplinary, outreach | no | total of 22 (50%) patients were started on a penicillin/penicillin derivative after clearance of allergy | none reported |
| Kyi L, Heke E, McPhee S, Ojaimi S, Barnes S. 2018. |  |  | none reported | none reported |
| Lecerf K, Chaparro J, Hehmeyer J, Hussain C, Macias C, Vegh M, et al. 2020. | Clinical decision support (CDS) integrated in electronic medical records (EMRs) |  | not reported | not reported |
| Leis, Palmay L, Ho G, Raybardhan S, Gill S, Kan T, et al. 2017. | Education, patient education material, consent | Training in first aid and cardiopulmonary resuscitation, an injection training course (pharmacists only), a half-day observation in the Drug Safety Clinic, and a half-day hands-on training session on BLAST | all 85 patients switched to preferred beta-lactam (NB not stated what the oral challenge was but presume a penicillin - contact authors) The proportion of days of penicillin nearly tripled (11% vs 32%; P < .0002), whereas carbapenem and fluoroquinolone use decreased by more than half (28% vs 13%; P < .0002).Receipt of preferred β-lactam therapy was increased among patients admitted to the critical care unit (odds ratio [OR], 2.1; 95% confidence interval [CI], 1.1–4.0; P = .02) BLAST was associated with a 4.5-fold greater odds of receiving preferred β-lactam therapy (95% CI, 2.4–8.2; P < .0001) | BLAST takes 1 hour at the bedside. With respect to secondary clinical outcomes, there was no significant difference in infection/treatment-related 30-day readmission or death, or hospital length of stay at any of the hospitals and no difference overall before or after adjusting for predictor variables (data not shown). Despite the increase in preferred β-lactam therapy, there was no change in the incidence of adverse drug reactions during the intervention period (3% vs 4%; P = .4). |
| Lin L, Nagtegaal JE, Buijtels PCAM, Jong E. 2020 | Education, education material, EMR CDS. | allergologist from the academic centre to give a talk on antibiotic allergy and the indications for further testing | The preferred antibiotic treatment was given to 40 of the 42 patients challenged. |  |
| Livirya S, Pithie A, Chua I, Hamilton N, Doogue M, Isenman H. 2020. | Patient education material |  |  |  |
| Lnumerables F, Fischer-Cartlidge E. | Coordination of care and management of care processes - Teams - Creating and delivering care through a multidisciplinary team of healthcare workers. |  | none reported | none reported |
| Lo SCR, Lacaria K, Mah A, Wong T, Mak R. 2020. | guideline, education | no | Five de-labelled patients subsequently tolerated courses of penicillin-derivatives. | Average time for testing was 1.56 h, Total cost of reagents was $1234.08 (for 9 patients). |
| Louden NJ, Hansen LA, Rimal A, Norton LE. 2021. | developed electronic allergy assessment tool. Our multidisciplinary team. developed allergy assessment tool. process standardization, allergy assessment tool creation, and staff training. an electronic tool was created to assist with allergy assessments and risk categorisation using Epic (EHR).A documentation template was also developed to minimize pharmacist work time. This template carried over the answers to patient interview questions, patient’s stratifi ed risk category, and associated clinical recommendation into a note accessible in the patient’s EHR. education and training meetings |  |  | all assessments performed by our pharmacists took 10 minutes or less. |
| Maguire M, Hayes BD, Fuh L, Elshaboury R, hi RG, Bor S, et al. 2020. | education, guideline | no | not reported | not reported |
| Marwood J, Aguirrebarrena G, Kerr S, Welch SA, Rimmer J. 2017. | education, resus equipment available, patient and GP informed, note on HER, communication between health providers | no | none reported | Total testing time in the ED was 120 min, including 30 min for skin prick and ID testing and 90 min for observation. For a senior registrar at AUD $55/h this would imply labour costs of AUS $110 per patient (reduced by batching patients in twos or threes) AUD $265 per kit |
| Mitchell AB, Ness RA, Bennett JG, Bowden JE, Elliott WV, Gillion AR, et al. 2021. | Daily report β-lactam allergies. electronic referral. training. On receipt of the PAC consult the ASP/ID pharmacist reviews the patient chart to further assess for eligibility and to determine whether oral challenge alone or skin testing followed by the oral challenge is required based on patient risk stratification. High risk patients discussed with allergy/immunology (A/I) physician. | Each inpatient Clinical Pharmacy Specialist is trained on this BLAA process, which includes patient screening, chart review, patient interviewing, and the BLAA template and note completion. Pharmacists must demonstrate competency in completing 5 BLAA notes with review from the ASP/ID pharmacist. Once training is completed, this process is integrated into the pharmacist’s everyday workflow. |  |  |
| Morjaria S, Inumerables F, Patel D, Cohen N, Seo S, Posthumus S, et al. 2021. | a collaborative team of nurses, pharmacists, administration, informatics, clinical nurse specialists (CNSs), and a physician from the infectious disease service. referral to service, patient info material, patient encouraged to inform other healthcare providers of de-label. standardized order set for PAT was created, as well as a subsequent EHR generated email. Physician and nursing notes were also modified to include structured fields for PAT. bedside hypersensitivity kit. Training and education. instructions and step-by-step procedural guidance. | The clinical nurse specialist was first trained to do PAT by an infectious disease physician skilled in the procedure and was then responsible for training the champions (nurses from the infusion unit). Competency was validated using a train-the-trainer methodology with a skills checklist and three observed successful patient testing experiences. Champions underwent didactic education and hands-on training and then trained other nurse colleagues. | significant decrease in aztreonam use in patients with a hematologic malignancy who underwent HSCT (17 of 46 [37%] before PAT versus 9 of 52 [17%] after PAT). | takes about 1.5 to 2 hours. Reduction in aztreonam use resulted in an approximate cost savings of $24,905. Overall, this program did not have additional costs to the organization because it used existing programmatic resources in the already established infusion centre and focused appointments for these patients during underused hours of the day, when resources were already available. Pharmacy time to prepare the medications was also calculated to be a minimal addition, which did not require new incremental positions to support the program. There is actual revenue generation potential because of billing for PAT and facility use. |
| Murphy K, Scanlan B, Coghlan D. 2015. | guideline, patient information, communication between healthcare providers, consent |  | none reported | none reported |
| . Trubiano, J. A., S. Vogrin, A. Copaescu, M. Nasra, A. Douglas, N. E. Holmes and K. Y. L. Chua (2022). | extensive education was undertaken to enable nursing and pharmacist assessment of inpatients using the AAAT |  | In the de-labelled cohort, there was significantly higher use of any penicillin, beta-lactam/beta-lactamase inhibitors, and narrow-spectrum penicillins, during the index admission and 90 days post-admission. There was a significant reduction in all other antibiotics except fluoroquinolones for the index post-testing period. | There was no difference in length of stay, readmission, or mortality between the groups |
| Nguyen CT, Sahbani O, Pisano J, Pursell K, Pettit NN. 2019. | guideline |  | not reported | The average time to complete an interview was 14.8 minutes |
| Parker N, Choo HF, Ghodrat M. 2018. | role expansion |  | 50% (14/28) have had their antimicrobial regimen changed. | These changes have also resulted in 2.54 days of decreased LOS and a cost savings of around $7,800/patient. |
| Patel R, Saccone N, Stock K, Utley S, Bouknight D. 2019. | guideline, Clinical decision support (CDS) integrated in electronic medical records (EMRs), protocol |  | 36/52 (69%) were switched to a β-lactam | none reported |
| Phung M, Vo T, Murfin B, Galbraith K, Barnes S, Coutsouvelis J. 2021. | validated tool, gave informed consent |  | three of nine de-labelled patients later prescribed penicillin |  |
| Rahbani P, Monroe-Duprey L. 2020. | PCN allergy report, generated from the electronic medical record |  | 39% decrease in vancomycin DOT/1000 in the surgical population |  |
| Rahbani P. 2019. | education, informed consent | no | Allergy assessments lead to a decrease in aztreonam DOT/1000 by 12% | not reported |
| Ravindran S, Beshir M, Wang S, i S, Hanson A, O’Driscoll T, et al. 2017. | guideline |  | Retrospective chart review showed significantly more patients received vancomycin (39.5% to 26.5%, p <0.05) and aztreonam (6% to 4.5%, p <0.05) prior to guideline implementation. Significantly more patients received any beta-lactam agent (21.5% to 35%, p=0.003) and penicillin G (1.5% to 6%, p = 0.03) after the guideline. | The estimated cost of vancomycin and aztreonam use decreased from $25,298 to $17,836. |
| Rimawi RH, Cook PP, Gooch M, Kabchi B, Ashraf MS, Rimawi BH, et al. 2013. | Governance, Health informatic systems, protocol |  | not reported | drug cost savings $225/per patient |
| Rimawi RH, Mazer MA. 2014. | guidelines, education | no | not reported | not reported |
| Sacco KA, Cochran BP, Epps K, Parkulo M, Gonzalez-Estrada A. 2019. | Stakeholder engagement, Education (lecture), protocol, | no | Cephalosporin and penicillin use increased by 121.2% (P = .03) and 256% (P = .04), respectively. The use of b-spectrum antibiotics decreased with vancomycin use down by a statistically significant 14.4% (P = .04). A trend in reduction of quinolones (33.3%, P = .31), carbapenems (81.9%, P =.08), and aztreonam (73.8%, P = .18) was also observed. | The length of stay was not increased by this intervention. The median length of stay was 2.33 days (IQR, 1.68-4.26 days) and 2.07 (IQR, 1.17-4.57 days) in the control and intervention cohorts respectively (not different). |
| Savic L, Gurr L, Kaura V, Toolan J, oe JAT, Hopkins PM, et al. 2019. | protocol, resus equipment and personnel available, communication between healthcare providers |  | not reported | not reported |
| Shannon KT, Krop LC. 2016. | guideline/, education, access to allergy experts | All clinical pharmacists receive education on the hospital-wide protocol and available literature regarding penicillin allergies and are asked to use their clinical judgment to make recommendations with the protocol and educational materials as a guide | no impact on abx course length for all infections. For non-deep-seated infections: 8.6(5.5) and 11.2(6.8) days in the intervention and control groups, respectively (P = 0.018). | There were no significant statistical differences in the mean length of hospital stay, all-cause mortality, 30-day all-cause readmission rate, or adverse events between groups. Similar to all-cause 30-day readmission rates, readmissions secondary to recurrent infection were similar between groups at 5 (8%) of 63 in-patients in the intervention group and 3 (5%) of 63 patients in the control group. Although not statistically significant, there were fewer deaths in the intervention group at 1 (2%) of 63 in-patients compared with 4 (6%) of 63 patients in the control group (P = 0.168). |
| Sigona NS, Steele JM, Miller CD. 2016. | guidelines, health informatics, education |  | not reported | not reported |
| Skibba N, Fischer J, Loecker B. |  |  | Initial analysis shows a positive cost-benefit with penicillin allergy skin testing. | none reported |
| Smibert O, Douglas A, Devch, M., Lambros B, Stevenson W, et al. 2018. | outreach, team |  | 88% (14/16) patients that were prescribed antibiotics received a narrow-spectrum β-lactam. | not reported |
| Sneddon J, Cooper L, Ritchie N, Steele C, Spears M, McEwen J, et al. 2021. | multi-professional steering group, algorithm, supporting information for clinical teams and patients, written consent for the oral challenge, patient information leaflet |  |  |  |
| Song Y-C, Nelson ZJ, Wankum MA, Gens KD. 2021. | A report within the electronic health record (EHR) to identify patients. A de-label flag alerts prescriber to de-label status. Progress note added to the EHR. patient education material. standardized checklist for conducting the interview. Hospital’s Antimicrobial Stewardship MDT Committee oversight. patient consent |  |  | The average time spent during patient interviews was 5.2 min per patient |
| Steenvoorden L, Bjoernestad EO, Kvesetmoen T-A, Gulsvik AK. 2021. | electronic patient record system, Informed consent was obtained from all study participants. an emergency kit with adrenalin was present during the observation period. |  | Twenty-six of the 57 patients who were tested with the oral provocation were under ongoing antibiotic treatment during inclusion. The doctors responsible for in-hospital treatment of these patients were informed. In 11 (42%) of the 26 patients the treating doctor decided it was possible to narrow their ongoing treatment to penicillin immediately after testing. Seven patients were switched to penicillin G/V, the others were changed to either Ampicillin, Amoxicillin or Cloxacillin. For 42% of these patients, their in-hospital antibiotic treatment was changed from a non-penicillin to a penicillin, which shows an immediate effect of inpatient penicillin allergy de-labelling. |  |
| Stein A, MacBrayne C, Yang C, Sarin T, Hicks A, Searns J, et al. 2020. | protocol |  | none reported | none reported |
| Stone CA, Stollings JL, Lindsell CJ, Dear ML, Buie RB, Rice TW, et al. 2020. | protocol |  | several patients went on to safely tolerate multiple doses of a penicillin 7/33 (21%) or a cephalosporin 15/33 (45%). | not reported |
| Taremi M, Artau A, Foolad F, Berlin S, White C, Jiang Y, et al. 2019. | PST team, the study was approved by the institutional Quality Improvement Assessment Board, a report of patients with PCN allergy was generated, patient verbal consent, Patients were given a pocket card on which test results were documented and could be shared with their health care providers at other institutions, protocol, outreach, patient pathway, training (ST so F2F) | no | Of the 39 patients who continued antibiotics, 20 (51%) were changed to PBA immediately after negative PST and oral challenge results, with piperacillin/tazobactam being the most prescribed agent. There were 266 days of PBA use as a direct result of PST. No patient experienced a type I allergic reaction while receiving PBA therapy. PBA agent was used in 40 of readmissions (34%). There were 1220 days of b-lactam use in readmitted patients, including 336 days of PBA therapy. No reported allergic drug reactions were associated with b-lactam during any of the subsequent admissions Of the 95 patients who were negative on PST and oral challenge, 51 (54%) were successfully prescribed PCN-based antimicrobials during initial admission or throughout the follow-up period, which consequently resulted in decreased aztreonam use. |  |
| Torney N, Tiberg M. 2018. | education | no | The most common antibiotics prior to PAST were vancomycin, cefepime, and fluoroquinolones. The most common antibiotics after PAST were penicillin, piperacillin/tazobactam, and amoxicillin/clavulanate. Of the 50 patients that were transitioned to a preferred β-lactam, the number of days of alternative antibiotics avoided ranged from 2 to 180, with a mean of 22.2 days and median of 11 days |  |
| Torney NP, Tiberg MD. 2021. | Allergists consulted as needed. institutional review board approval. training. Testing protocol perform a thorough allergy history. formal request for PAST by a provider. collaboration with pharmacy, ID physicians and the hospitalist. | annual training program changed to incorporate the Penicillin Allergy Assessment and Skin Testing Certificate program, which includes 11 hours of didactic webinars and 4 hours of live demonstration, for a total of 15 hours of continuing education intended for all licensed healthcare practitioners. To maintain competency after the initial training, 2 penicillin allergy skin tests must be completed per year by each individual certified to perform PAST | Of the 90 patients who started PAST, 76 (84.4%) were transitioned to preferred β-lactam therapy. the antibiotics most commonly used prior to PAST were vancomycin, cefepime, and metronidazole The antibiotics most commonly used after PAST were penicillin, piperacillin/tazobactam, and ampicillin/sulbactam. In the 90 patients who started PAST, alternative antibiotics were avoided for a total of 1,568 days, with a median (interquartile range [IQR]) of 11 (6-18) days for each patient. | This entire process took around 2 to 2.5 hours once the team was comfortable compounding the products, performing PAST, and documenting and sharing the results. |
| Trubiano JA, Smibert O, Douglas A, Devch, Misha, Lambros B, et al. 2018 | guideline | no | there was a greater likelihood of administration of a penicillin-based antibiotic being prescribed in the 90 days post challenge (22/26, 84.6%) when compared with the than 90 days pre-challenge (1/31, 3.2%; P < .001). There was a noted reduction in third- and fourth-generation cephalosporin usage post-testing (2/23, 8.7%, vs 16/23, 69.6%; P = .0001). |  |
| Trubiano JA, Thursky K, Stewardson AJ, Urbancic K, Worth LJ, Sutherl, et al. 2017. |  |  | Post AAT (all antibiotic allergies) there was an increase in prescribing of guideline-preferred antibiotic therapies (post AAT, 83% verses pre AAT, 11.6%; P=0.0001). A reduction in glycopeptide, carbapenem, lincosamide and fluoroquinolone antibiotic courses and an increase in penicillin and beta-lactam/beta-lactamase inhibitor use. Prescribers were more likely to select narrow-spectrum penicillins, narrow-spectrum beta-lactams and beta-lactam/beta-lactamase inhibitors post AAT and less likely to select restricted antibiotics. The proportion of appropriate antibiotic prescriptions was higher post AAT (95% versus 62%; OR,13.25 (CI, 5.22-33.61) | none reported |
| Vyles D, Chiu A, Routes J, Castells M, Phillips EJ, Visotcky A, et al. 2020. | guideline |  |  | Children who received an oral challenge had an increased LOS (216 min vs 151 min, p < 0.01, CI 25-103). |
| Wall GC, Peters L, Leaders CB, Wille JA. 2004. | Education, governance, protocol | At the request of the pharmacy department, a board-certified allergist trained selected pharmacists in using the penicillin skin test and served as the program’s medical director. Training sessions were convened with pharmacists every three to four weeks for four months. The training included didactic lectures about hypersensitivity reactions and a review of official guidelines pertaining to allergic drug reactions. Practical training included administering both the scratch test and the intradermal test to volunteers and other staff. The first attempts at intradermal testing were done with 0.9% sodium chloride injection only, then with histamine (to observe the wheal-and-flare reaction). Finally, complete testing with both major and minor determinants was performed. Yearly assessment of intradermal test technique and penicillin allergy knowledge. | vancomycin and levofloxacin avoidance, |  |
| Wong J, Timberlake K, Atkinson A, Science M. 2018. | Guideline, communication with other care providers. |  |  |  |
| Wrenn R, Sarubbi C, Kleris R, Drew R, Moehring R, Lugar P, et al. 2017. | Education, access to expert | no | 16 (76%) negative SPT patients had their current antimicrobial therapy discontinued or de-escalated. | not reported |

**Appendix 7. Efficacy and safety by de-label method**

***Allergy testing methods***

Of those reporting a single testing method, six used DDL on history alone,^38,64,67,73,75,81^ 17 used DDPT,^3,31,37,41,43,46,48,62,65,72,74,76,77,83,84,94,95^ sixteen used ST/DPT,^27,30,33,42,45,49,52,54,56,61,66,68,70,71,82,85^ ten studies reported ST without standardised DPT, or it was unclear if the patients received a DPT.^12,35,39,40,42,44,58-60,88^ Nineteen reported a combination of testing methods (history alone and DDPT ,^32,36,47,51,63,69,91,93^ history alone and ST/DPT or ST(+/-DPT),^29,34,53,78,80,90^ history alone, DDPT and ST/DPT^50,55,57,96^ and one study did not report the testing method.^28^

*Proportion of assessed patients who were de-labelled by DDL, DDPT and ST/DPT*

Nineteen of the twenty-three studies reporting on DDL based on history alone had complete data showing that of the 4972 assessed patients 795 (16.0%) were de-labelled.^29,32,34,36,47,50,51,53,57,63,64,67,69,75,78,81,90,93,96^ Twenty-one of thirty-two studies reporting DDPT had complete data on 4893 assessed patients of which 872 (17.8%) were successfully de-labelled.^3,31,32,36,37,46,47,50,51,57,62,63,69,72-74,76,77,90,93,96^ Sixteen of twenty-five studies reporting ST/DPT had complete data on 4978 assessed patients of which 1685 (33.8%) were successfully de-labelled. ^27,34,45,49,50,52,56,57,61,66,70,71,78,85,90,96^

*Proportion of tested patients who were de-labelled and harmed by DDL, DDPT and ST/DPT*

Nineteen of twenty-three studies reporting DDL had complete data on 744 patients who were offered DDL of whom 732 (98.4%) were successfully de-labelled without reported harm.^29,32,34,47,50,51,53,55,57,63,64,67,69,75,78,80,90,93,96^ Those reported to be unsuccessfully de-labelled were patients who refused to have their allergy label removed.^50,75^ Twenty-five of thirty-one studies reporting on DDPT had complete data on 1361 tested patients, of which 1293 (95.0%) were successfully de-labelled.^3,31,32,37,41,46-48,50,51,55,57,62,63,65,69,72-74,76,77,83,93,94,96^ In twenty-seven of thirty-one studies with complete data on DDPT, 38 (3.5%) of 1398 tested patients experienced harm. ^3,31,32,37,41,46-48,50,51,55,57,62,63,65,69,72-74,76,77,83,93-96^ Twenty-one of twenty-five studies reporting ST/DPT had complete data on 2199 patients of which 1936 (88.0%) were successfully de-labelled.^27,30,34,42,45,49,50,52,54-57,61,66,68,70,71,78,85,90,96^ Twenty-three of twenty-five reported complete data for 2320 tested patients of which 30 (1.3%) experienced harm with the remainder having equivocal or positive skin tests.^27,30,33,34,42,45,49,50,52,54-57,61,66,68,70,71,78,80,85,90,96^

*Skin testing without standardised DPT*

Twelve studies did not report a standardised challenge post skin testing. A DPT was either not given,^12,88^optional,^29,53,60^ or it was not clear whether a DPT was given,^35,39,40,42,44^ or the therapeutic antibiotic was administered which may or may not have been a penicillin.^58,59^ We have excluded these studies from the meta-analysis as this is not a recognised de-label method.^9,10^ Of 611 patients tested, 560 (91.7%) were successfully “de-labelled” with harm reported in 3 (0.5%) patients.

**Efficacy and safety by de-label method table**

*low risk patients (intolerance, delayed non-severe rash, unknown reaction), moderate risk patients (IgE), high risk patients (SCAR). DDL = direct de-label on history alone, DPT= direct provocation, ST/OC= skin testing followed by oral challenge, PT= patch testing

| Reference | Setting / context | adult or paeds | Allergy risk category | The penA de-label method used | Number of patients in study (assessed) | number of pen A patients penA tested | The number of patients with a penicillin allergy record successfully de-labelled | proportion of patients de-labelled (screened) | proportion of patients de-labelled (tested) | number of patients experiencing unintended harm | Proportion experiencing unintended harm |
| --- | --- | --- | --- | --- | --- | --- | --- | --- | --- | --- | --- |
| Adkinson NF, Thompson WL, Maddrey WC, Lichtenstein LM. 1971. | inpatients | adults | not clear | ST/OC | 66 | 66 | 37 | 56.16% | 100% | 1 | 6.25% |
| Allen HI, Vazquez-Ortiz M, Murphy AW, Moylett EM. 2020. | outpatients | paediatrics | low risk | DPT | 136 | 102 | 99 | 73% of screened | 97% of tested | 3 | 2.94% |
| Bauer ME, MacBrayne C, Stein A, Searns J, Hicks A, Sarin T, et al. 2021. | inpatients | paediatrics | low risk | DDL, DPT | 701 | combined = 83, (DDL=31, DPT = 52) | combined = 82, (DDL=31, DPT = 51) | combined = 11.7% (DDL= 4.42%, DPT = 7.28%) | combined = 98.8% (DDL = 100%, DPT = 98.08%) | 1 | DPT = 1.92% |
| Blackwell W, Khan D. 2020. | inpatients | adults | not clear | ST/OC | 1926 | 850 | 754 | 39% of screened | 89% of tested | 15 | 1.80% |
| Blumenthal KG, Li Y, Hsu JT, Wolfson AR, Berkowitz DN, Carballo VA, et al. 2019. | inpatients | adults | low risk | DPT | 862 | 76 | 73 | undeterminable | 96.05% | 3 | 4% |
| Blumenthal KG, Shenoy ES, Varughese C, Hurwitz S, Hooper D, Banerji A. 2015. | inpatients | adults | low risk | DPT | Not stated | 36 | undeterminable (outcomes of challenges not reported) | undeterminable (outcomes of challenges not reported) | undeterminable (outcomes of challenges not reported) | undeterminable (beta-lactam study) | undeterminable (beta-lactam study) |
| Chen JR, Tarver SA, Alvarez KS, Tran T, Khan DA. 2017. | inpatients | adults | moderate risk | ST/OC | 252 | 252 | 228 | 90.5% of screened | 90.5% of tested | 1 | 0.40% |
| Chen JR, Tarver SA, Alvarez KS, Wei W, Khan DA. 2018. | inpatients | adults | low, moderate risk | ST/OC | 77 | 21 | 21 | 27% of screened | 100% of tested | 0 | none identified |
| Chua KYL, Vogrin S, Bury S, Douglas A, Holmes NE, Tan N, et al. 2020. | inpatients | adults | low risk | DDL, DPT | 1225 | Combined (DDL/DPT) = 361 (DDL 161, DPT 200) | Combined = 355 (194 DPT, 161 DDL) | Combined =28.98% (15.84% DPT, 13.14% DDL) | Combined = 98.33% (97% of DPT tested; 100% DDL) | 6 | overall (DDL/DPT combined) = 1.67% DPT =3%, DDL 0% |
| Devch, M., Kirkpatrick CMJ, Stevenson W, Garrett K, Perera D, et al. 2019. | inpatients | adults | low risk | DDL, DPT, ST/OC | 309 | Combined =47 (22 DDL; 21 DPT; 4 ST/OC) | Combined = 38 (DDL = 14, DPT = 20, ST/OC=4) | Combined = 12.30% (DDL = 4.5%, DPT= 6.8%, ST/OC=1.3%) | Combined = 97.43% (DDL=63.64%, DPT =95.24%, ST/OC=100%) | 1 | combined = 2.56% (DPT = 5%) |
| du Plessis T, Walls G, Jordan A, Holl, J. D. 2019. | inpatients | adults | low risk | DDL, DPT | 274 | Combined = 194 (DDL=160, DPT=34) | Combined = 191 (DDL= 160, DPT =31) | Combined = 69.7% (DDL = 58.4%, DPT = 11.3%) | Combined = 98.5% (DDL = 100%; DPT = 91.2%) | 3 | combined = 1.6% (8.8% of DPT cohort) |
| Eischens MR, Wolf LM, Dumkow LE, Anderson AM, Jameson AP, Br, et al. | ED | adults | not reported | not reported | Not reported | undeterminable ("beta-lactam challenge") | undeterminable | undeterminable | undeterminable | undeterminable (beta-lactam study) | undeterminable (beta-lactam study) |
| Englert E, Weeks A. 2019. | inpatients | adults | moderate risk | ST (-OC) | 87 | 23 | 21 | 24.13% | 91.3 | 1 | 4.35% |
| Foolad F, Berlin S, White C, ice, Dishner E, Jiang Y, et al. 2019. | inpatients | adults | moderate risk | ST/OC | 129 | 49 | 46 | 35.65% of screened | 93.88% of tested | 1 | 2.10% |
| Gaudreau S, Bourque G, Cote K, Nutu C, Beauchesne M-F, Longpre A-A, et al. 2021. | inpatients | adults | low, moderate risk | DDL, ST/OC | 406 | combined 125 (DDL 88, ST/OC 37) | combined 112 (DDL 88, ST/OC 24) | combined 27.59% (DDL = 21.67%, ST/OC = 5.91%) | combined = 89.6% (DDL=100%, ST/OC=64.86%) | 3 | combined = 2.4% (ST/OC = 8.1%, DDL = 0) |
| Griffith NC, Justo JA, Winders HR, Al-Hasan MN, Mediwala KN, Bookstaver PB. 2020. | inpatients | adults | not clear | DDL, ST/(+/-)OC | 161 | Combined = 78 (DDL = 36, ST (+/- OC) = 42) | Combined = 72 (DDL = 36, ST(+/-OC) = 36) NB only 3 given OC | Combined = 44.72 % (DDL=22.36, ST= 22.36%) | Combined = 92.30% (ST = 85.71% (but only 3 pts OC), DDL = 100%) | 0 | none identified |
| Gugkaeva Z, Crago JS, Yasnogorodsky M. 2017 | inpatients | adults | moderate risk | ST/OC | unknown | 53 | 51 | undeterminable | 96.22% | 0 | not reported |
| Ham Y, Sukerman ES, Lewis JS, Tucker KJ, Yu DL, Joshi SR. 2021. | inpatient | adults | low, moderate & high risk | DDL, DPT, ST/OC | unknown | 50 | combined = 48 (DDL=20, DPT = 28, ST/OC=1) | undeterminable | combined = 96% (DDL = 100%, DPT = 93.33%, ST/OC = 100%) | 2 (DPT group) | Combined 4% (DPT = 6.67%) |
| Harmon S, Richardson T, Simons H, Monforte S, Fanning S, Harrington K. 2020 | inpatients | adults | moderate risk | ST/OC/IVC | 47 | 31 | 27 | 57% of screened | 87.10% of tested | 2 | 3.70% |
| Harper HM, Sanchez M. 2021. | inpatient | adults | moderate risk | DDL, DPT, ST, ST/OC | 53 | 35 (24 ST/OC, DPT 4, ST (no OC) 2, DDL 5) | 35 (24 ST/OC, DPT 4, ST (no OC) 2, DDL 5) | 66.04% | COMBINED = 100% (ST/OC = 100%, DPT= 100%, ST (no OC) =100%, DDL = 100%) | 0 | 0 |
| Harris AD, Sauberman L, Kabbash L, Greineder DK, Samore MH. 1999. | inpatients | adults | moderate risk | ST then required antibiotic | 100 | 44 | 38 | 38% | 86.36% of tested | 1 | 2.27% |
| Heil EL, Bork JT, Schmalzle SA, Kleinberg M, Kewalramani A, Gilliam BL, et al. 2016. | inpatients | adults | moderate risk | ST then required antibiotic | 90 | 76 | 35 (these are the 55% switched to a penicillin) the rest were switched to a cephalosporin or carbapenem. As we do not know how many were given the optional amoxicillin OC we cannot say how many were de-labelled except that these 35 pts received penicillin | 38.89% screened | 46.05% tested | 0 | 0 |
| Jones BM, Avramovski N, Concepcion AM, Crosby J, Bl, M C. 2019. | inpatients | adults | moderate risk | DDL, ST/(+/-)OC | 116 | Combined (DDL, ST) = 107 (DDL=6, ST=100 (NB only 6 given amox OC) | Combined = 104 (DDL=6, ST=98) | Combined 90.52% (DDL=5.17%, ST=84.48%) | Combined 98.11% (DDL=100%, ST=98%) (NB ST only without OC). 7 patients did undergo OC: 100% negative | 0 | 0 |
| Jones BM, Bl, M. C. 2017. | inpatients | adults | moderate risk | ST/(+/-)OC | not reported | 46 | 36 | undeterminable | 78.26% of tested (ST only) ad switched to either penicillin or cephalosporin therapy (not quantified) | 0 | 0 |
| Jones BM, Gamble K, Sizemore S, Bl, M. C. 2019. | inpatients | adults | moderate risk | DDL, DPT, ST/OC | 162 | Combined = 42 (DDL=34, DPT=2, ST/OC=6) | Combined = 42 (DDL=34, DPT=2, ST/OC=6) | 25.93% of screened (DDL=20.99%, DPT=1.23%, ST/OC=3.70%) | 100% | 0 | 0 |
| Kleris R, Sarubbi C, Wrenn R, Anderson D, Lugar PL. 2018. | inpatients | not clear | not reported | ST/OC | unknown | 47 | 44 | undeterminable | 93.62% | 0 | 0 |
| Kyi L, Heke E, McPhee S, Ojaimi S, Barnes S. 2018. | inpatients | adults | low risk | DPT | 181 | 35 | 34 | 18.78% of screened | 97.14% of tested | 1 | 3% |
| Lecerf K, Chaparro J, Hehmeyer J, Hussain C, Macias C, Vegh M, et al. 2020. | inpatients | paediatrics | low risk | DDL, DPT | 114 | Combined (DPT/DDL) = 22 (6 DPT, 16 DDL) | Combined 22 (DPT=6, DDL=16) | Combined 19.30% (DPT=5.26%, DDL=14.04) | Combined 100% (DPT 100%, DDL 100%) | 0 | 0 |
| Leis, Palmay L, Ho G, Raybardhan S, Gill S, Kan T, et al. 2017. | inpatients | adults | moderate risk | ST/OC | 154 | 90 | 85 | 55.19% of screened | 94.44% of tested | 1 | 1.17% |
| Lin L, Nagtegaal JE, Buijtels PCAM, Jong E. 2020 | inpatients | adults | low risk | DPT | 93 | 42 | 40 | 43.01% of screened | 95.24% of tested | 2 | 5% |
| Livirya S, Pithie A, Chua I, Hamilton N, Doogue M, Isenman H. 2020 | inpatients | adults | low risk | DDL, DPT | 224 | Combined 112 (DDL=71, DPT=41) | Combined 112 (DPT 41, DDL 71) | Combined 50% (DDL=31.7%, DPT=18.30%) | Combined 100% (DPT = 100%, DDL=100%) | 0 | 0 |
| Lnumerables F, Fischer-Cartlidge E. | inpatients & outpatients | adults | not reported | ST/IVC | unknown | 98 | 92 | undeterminable | 93.88% | 3 | 3% |
| Lo SCR, Lacaria K, Mah A, Wong T, Mak R. 2020. | outpatients | adults | not reported but defined by authors as "low risk" | DDL, ST/OC | 15 | Combined 12 (DDL=3, ST/OC=9) | Combined = 10 (DDL= 3, ST/OC=8) | Combined = 66.67% (DDL=20%, ST/OC=53.33%) | Combined = 83.33% (DDL=100%, ST/OC= 88.89%) | 1 | ST/OC = 8.33% |
| Louden NJ, Hansen LA, Rimal A, Norton LE. 2021. | inpatients | paediatrics | low risk | DDL | 11 | 2 | 2 | 18.18% | 100% | 0 | 0 |
| Maguire M, Hayes BD, Fuh L, Elshaboury R, hi RG, Bor S, et al. 2020. | ED | adults | low risk | DPT | undeterminable (beta-lactams reported) | 37 | 33 | undeterminable | 89.19% of tested | 4 | 10.81% |
| Marwood J, Aguirrebarrena G, Kerr S, Welch SA, Rimmer J. 2017. | ED | adults | moderate risk | ST/OC | 103 | 100 | 81 | 78.64% | 81% | 3 | 3.60% |
| Mitchell AB, Ness RA, Bennett JG, Bowden JE, Elliott WV, Gillion AR, et al. 2021. | inpatients | adults | low risk | DDL | 278 | DDL = 62, DPT/ST/OC = 32 (not defined the testing in this group) | DDL = 62, DPT/ST/OC = 24 (not defined the testing in this group) | DDL = 22.3%; Unble to report the allergy testing outcome by testing method (i.e., whether DPT or ST/OC) | 100% | DDL = 0; DPT/ST/OC = no harm reported | 0 |
| Morjaria S, Inumerables F, Patel D, Cohen N, Seo S, Posthumus S, et al. 2021. | outpatients | adults | moderate risk | ST/IVC | unknown | 74 | 72 | indeterminable | 97.30% | 1 | 1.35% |
| Murphy K, Scanlan B, Coghlan D. 2015. | inpatients & outpatients | paediatrics | not reported but defined by authors as "low risk" | DDL, DPT | 40 | Combined = 40 (DDL=2, DPT=38) | Combined =38 (DPT=36, DDL=2) | Combined = 95% (DDL=5%, DPT = 90%) | Combined = 95% (DDL= 100%, DPT = 90%) | 2 | DPT = 5.26% |
| Nguyen CT, Sahbani O, Pisano J, Pursell K, Pettit NN. 2019. | inpatients | adults | low risk | DDL | 107 | unknown | 11 | 10% of screened | 100% | 0 | 0 |
| Parker N, Choo HF, Ghodrat M. 2018. | inpatients | adults | not reported | ST (unclear if received OC) | not reported | 35 | 28 | undeterminable | 80% | 0 | 0 |
| Patel R, Saccone N, Stock K, Utley S, Bouknight D. 2019. | inpatients | adults | not reported | DDL, DPT | 492 | Undeterminable | Combined = 99 (DDL=76, DPT=23) | Combined = 20.12% (DDL= 15.45, DPT= 4.67) | undeterminable ("23 patients de-labelled via DPT, 75 patients via DDL" (no data on numbers challenged)) | not reported | not reported |
| Phung M, Vo T, Murfin B, Galbraith K, Barnes S, Coutsouvelis J. 2021. | inpatients | adults | low risk | DPT | 77 | 10 | 9 | 12.99% | 90% | not reported, potentially 1 patient | 10% |
| Rahbani P, Monroe-Duprey L. 2020. | outpatients | paediatrics | not reported | ST (unclear if received OC) | not reported | 155 | 154 | indeterminable | 99.35% | not reported, potentially 1 patient | 0.65% |
| Rahbani P. 2019. | inpatients | not clear | not reported | DDL | not reported | not reported | undeterminable | undeterminable | undeterminable | 0 | 0 |
| Ravindran S, Beshir M, Wang S, i S, Hanson A, O’Driscoll T, et al. 2017. | inpatients | not clear | not reported | ST/OC | undeterminable (beta-lactams reported) | undeterminable | 200 | undeterminable | undeterminable | 0 | 0 |
| Rimawi RH, Cook PP, Gooch M, Kabchi B, Ashraf MS, Rimawi BH, et al. 2013. | inpatients | adults | moderate risk | ST/OC | 482 | 146 | 145 | 30.08% | 99.32% | 0 | 0 |
| Rimawi RH, Mazer MA. 2014. | inpatients | adults | moderate risk | ST/OC | 579 | 100 | 100 | 17.27% | 100% | 0 | 0 |
| Sacco KA, Cochran BP, Epps K, Parkulo M, Gonzalez-Estrada A. 2019. | inpatients | adults | low risk | DDL, DPT | 57 | undeterminable | undeterminable | undeterminable | not reported | 0 | 0 |
| Savic L, Gurr L, Kaura V, Toolan J, oe JAT, Hopkins PM, et al. 2019. | peri-operative | adults | low risk | DPT | 145 | 56 | 55 | 37.93% | 98.21% of tested | 1 | 1.79% |
| Shannon KT, Krop LC. 2016. | inpatients | adults | moderate risk | ST (-OC) | 148 | 9 | 8 | 5.41% (beta-lactam study, many were given cephalosporins) | 89% of tested | 0 | 0 |
| Sigona NS, Steele JM, Miller CD. 2016. | inpatients | adults | low risk | DDL | 32 | 3 | 3 | 9.38% | 100% | 0 | 0 |
| Skibba N, Fischer J, Loecker B. | inpatients | adults | not reported | ST (unclear if received OC) | not reported | not reported | not reported | undeterminable | not reported | 0 (but beta-lactam challenges) | 0 |
| Smibert O, Douglas A, Devch, M., Lambros B, Stevenson W, et al. 2018. | inpatients | adults | low risk | DPT | undeterminable | 29 | 29 | undeterminable | 100% of tested | 0 | 0 |
| Sneddon J, Cooper L, Ritchie N, Steele C, Spears M, McEwen J, et al. 2021. | inpatient, pre-op | adults | low risk | DPT | 112 | 92 | 90 | 80.36% | 97.82% | 1 | 1.09% |
| Song Y-C, Nelson ZJ, Wankum MA, Gens KD. 2021. | inpatients | adults | low risk | DDL | 66 | 16 | 12 | 18% | 75% | 0 | 0 |
| Steenvoorden L, Bjoernestad EO, Kvesetmoen T-A, Gulsvik AK. 2021. | inpatients | adults | low risk | DPT | 191 | 57 | 55 | 28.80% | 96.49% | 2 | 3.51% |
| Stein A, MacBrayne C, Yang C, Sarin T, Hicks A, Searns J, et al. 2020. | inpatients | paediatrics | low risk | DPT | not reported (increase from 2% to 9.5% of patients de-labelled) | 17 | 17 | undeterminable | 100% | 0 | 0 |
| Stone CA, Stollings JL, Lindsell CJ, Dear ML, Buie RB, Rice TW, et al. 2020. | inpatients | adults | not reported but defined by authors as "low risk" | DPT | 137 | 33 | 33 | 24.09% of screened | 100% of tested | 0 | none identified |
| Taremi M, Artau A, Foolad F, Berlin S, White C, Jiang Y, et al. 2019. | inpatients | adults | moderate risk | DDL, ST/OC | 218 | ST/OC = 100, DDL=6 | ST/OC = 95, DDL =6 | ST/OC = 43.58%, DDL=2.75% | ST/OC =95%, DDL=100% | 1 | ST/OC 1% |
| Torney N, Tiberg M. 2018. | inpatients | adults | moderate risk | ST (unclear if received OC) | not reported | 58 | 56 | undeterminable | 96.56% | 0 | 0 |
| Torney NP, Tiberg MD. 2021. | inpatients | adults | moderate risk | ST/OC | unknown | 90 | 84 | indeterminable | 93.33% | 1 | 1.11% |
| Trubiano JA, Smibert O, Douglas A, Devch, Misha, Lambros B, et al. 2018 | inpatients & outpatients | adults | low risk | DPT | 195 | 46 | 46 | 23.59% of screened | 100% of tested | 0 | 0 |
| Trubiano JA, Thursky K, Stewardson AJ, Urbancic K, Worth LJ, Sutherl, et al. 2017. | inpatients | adults | low, moderate & high risk | ST/OC, DPT, PT | unknown | Combined = 66 | Combined = 55 | undeterminable | 83.33% | 1 | 1.82% |
| Trubiano, J. A., S. Vogrin, A. Copaescu, M. Nasra, A. Douglas, N. E. Holmes and K. Y. L. Chua (2022). | inpatient | adults | low risk | DPT | 1336 | 278 (478-200 from Chua paper (duplicate data) | 264= (458 -194 Chua paper) | 34.28% | 94.96% |  |  |
| Vyles D, Chiu A, Routes J, Castells M, Phillips EJ, Visotcky A, et al. 2020. | ED | paediatrics | not reported but defined by authors as "low risk" | DPT | 376 | 37 | 36 | undeterminable | 97.30% | 1 | 3% |
| Wall GC, Peters L, Leaders CB, Wille JA. 2004. | inpatients | adults | moderate risk | DDL, ST/OC | unknown | Combined = 26 (DDL = 3, ST/OC = 23) | 25 | Combined = 96.15% (ST = 95.65%, DDL = 100%) | 96.15% (of those tested, NB ST /-OC only) | 0 (NB penicillin & other beta-lactam test doses) | 0 |
| Wong J, Timberlake K, Atkinson A, Science M. 2018. | inpatients | paediatrics | not reported | DPT | undeterminable (beta-lactams reported) | undeterminable (beta-lactams reported) | 11 | undeterminable | underminable | 0 | none identified |
| Wrenn R, Sarubbi C, Kleris R, Drew R, Moehring R, Lugar P, et al. 2017. | inpatients | adults | not clear | ST (unclear if received OC) | 107 | 23 | 21 | 21.50% of screened | 91.30% of tested | 0 | 0 |

**Appendix 8. EPOC domains**

| **Domain** | | | **Delivery Arrangements** | | | | | | | | | | | | | | | | | | | | | | | | | |  | | |
| --- | --- | --- | --- | --- | --- | --- | --- | --- | --- | --- | --- | --- | --- | --- | --- | --- | --- | --- | --- | --- | --- | --- | --- | --- | --- | --- | --- | --- | --- | --- | --- |
| **Category** | | | **Coordination of care and management of care processes** | | | | | | | | | | | | | **Information and communication technology (ICT)** | | | | | | **Who provides care and how the healthcare workforce is managed** | | | **How and when care is delivered** | | **Where care is provided and changes to the healthcare environment** | |  | | |
| **Subcategory**  **Reference** | | | **Teams** | | **Shared decision-making** | | | **Communication between providers** | | **Care pathways** | | | **Packages of care** | | | **Health information systems** | | **The use of information and communication technology** | | | | **Role expansion or task shifting** | | | **Triage** | | **Outreach services** | | **Totals** | | |
| Adkinson NF *et al.* 1971. | | | ✓ | | ✓ | | |  | |  | | |  | | |  | |  | | | |  | | |  | |  | | **2** | | |
| Allen HI, *et al.* 2020. | | |  | | ✓ | | |  | | ✓ | | | ✓ | | |  | |  | | | |  | | | ✓ | | ✓ | | **5** | | |
| Bauer ME, *et a*l. 2021. | | |  | | ✓ | | |  | | ✓ | | | ✓ | | | ✓ | |  | | | |  | | |  | |  | | **4** | | |
| Blackwell W, Khan D. 2020. | | |  | |  | | |  | |  | | | ✓ | | |  | |  | | | |  | | |  | |  | | **1** | | |
| Blumenthal KG *et al.* 2019. | | | ✓ | |  | | |  | | ✓ | | | ✓ | | | ✓ | |  | | | |  | | |  | |  | | **4** | | |
| Blumenthal KG *et al.* 2015. | | | ✓ | | ✓ | | |  | | ✓ | | | ✓ | | |  | |  | | | |  | | |  | |  | | **4** | | |
| Chen JR *et al.*2017. | | |  | |  | | |  | | ✓ | | | ✓ | | |  | | ✓ | | | |  | | |  | | ✓ | | **4** | | |
| Chen JR *et al.* 2018. | | |  | |  | | |  | | ✓ | | | ✓ | | |  | |  | | | |  | | |  | | ✓ | | **3** | | |
| Chua KYL *et al.* 2020. | | | ✓ | | ✓ | | |  | | ✓ | | | ✓ | | |  | |  | | | |  | | |  | |  | | **4** | | |
| Devch, M. *et al.* 2019. | | | ✓ | | ✓ | | |  | | ✓ | | | ✓ | | | ✓ | |  | | | |  | | | ✓ | | ✓ | | **7** | | |
| du Plessis T, *et al.*2019. | | | ✓ | | ✓ | | |  | | ✓ | | | ✓ | | |  | |  | | | |  | | |  | |  | | **4** | | |
| Eischens MR, *et al.* | | | ✓ | |  | | |  | |  | | |  | | |  | |  | | | |  | | |  | |  | | **1** | | |
| Englert E, Weeks A. 2019. | | |  | | ✓ | | |  | | ✓ | | | ✓ | | | ✓ | |  | | | |  | | |  | | ✓ | | **5** | | |
| Foolad F, *et al.* 2019. | | | ✓ | | ✓ | | | ✓ | | ✓ | | | ✓ | | |  | |  | | | |  | | |  | | ✓ | | **6** | | |
| Gaudreau S, *et al*. 2021. | | |  | | ✓ | | |  | | ✓ | | |  | | | ✓ | |  | | | |  | | |  | | ✓ | | **4** | | |
| Griffith NC *et al.* 2020. | | |  | | ✓ | | | ✓ | | ✓ | | | ✓ | | | ✓ | | ✓ | | | | ✓ | | |  | | ✓ | | **8** | | |
| Gugkaeva Z. *et al.* 2017 | | |  | | ✓ | | |  | | ✓ | | | ✓ | | |  | |  | | | |  | | |  | | ✓ | | **4** | | |
| Ham Y, *et al.* 2021. | | |  | | ✓ | | | ✓ | | ✓ | | | ✓ | | | ✓ | | ✓ | | | |  | | |  | | ✓ | | **7** | | |
| Harmon S, *et al.* 2020 | | |  | | ✓ | | |  | | ✓ | | | ✓ | | |  | |  | | | |  | | |  | | ✓ | | **4** | | |
| Harper HM, Sanchez M. 2021. | | |  | |  | | | ✓ | | ✓ | | | ✓ | | |  | | ✓ | | | |  | | |  | | ✓ | | **5** | | |
| Harris AD *et al.* 1999. | | | ✓ | |  | | |  | | ✓ | | | ✓ | | | ✓ | |  | | | |  | | |  | |  | | **4** | | |
| Heil EL, *et al.* 2016. | | |  | | ✓ | | | ✓ | | ✓ | | | ✓ | | |  | |  | | | |  | | |  | | ✓ | | **4** | | |
| Jones BM, 2019. | | | ✓ | |  | | |  | | ✓ | | | ✓ | | |  | | ✓ | | | |  | | |  | | ✓ | | **5** | | |
| Jones BM, Bland, MC. 2017. | | | ✓ | |  | | |  | | ✓ | | | ✓ | | | ✓ | |  | | | |  | | |  | | ✓ | | **5** | | |
| Jones BM, *et al.* 2019. | | |  | |  | | |  | |  | | |  | | |  | |  | | | |  | | |  | | ✓ | | **1** | | |
| Kleris R, *et al.*2018. | | | ✓ | |  | | |  | |  | | |  | | |  | |  | | | |  | | |  | | ✓ | | **2** | | |
| Kyi L, *et al.* 2018. | | | ✓ | |  | | |  | |  | | |  | | |  | |  | | | |  | | |  | |  | | **1** | | |
| Lecerf K, *et al*. 2020. | | |  | |  | | |  | | ✓ | | |  | | |  | |  | | | |  | | | ✓ | | ✓ | | **3** | | |
| Leis *et al.* 2017. | | | ✓ | | ✓ | | | ✓ | | ✓ | | | ✓ | | |  | |  | | | |  | | |  | |  | | **5** | | |
| Lin L *et al.* 2020 | | |  | | ✓ | | |  | | ✓ | | | ✓ | | |  | |  | | | |  | | |  | |  | | **3** | | |
| Livirya S, et al. 2020. | | | ✓ | | ✓ | | |  | | ✓ | | | ✓ | | |  | |  | | | |  | | |  | | ✓ | | **5** | | |
| Lnumerables F, Fischer-Cartlidge E. | | | ✓ | | ✓ | | |  | | ✓ | | | ✓ | | |  | |  | | | |  | | |  | | ✓ | | **5** | | |
| Lo SCR *et al* 2020. | | |  | |  | | |  | |  | | | ✓ | | |  | |  | | | |  | | |  | |  | | **1** | | |
| Louden NJ, *et al.* 2021. | | | ✓ | |  | | |  | | ✓ | | | ✓ | | | ✓ | | ✓ | | | |  | | |  | |  | | **5** | | |
| **Domain** | | | **Delivery Arrangements** | | | | | | | | | | | | | | | | | | | | | | | | | |  |  |  |
| **Category** | | | **Coordination of care and management of care processes** | | | | | | | | | | | | | **Information and communication technology (ICT)** | | | | | | **Who provides care and how the healthcare workforce is managed** | | | **How and when care is delivered** | | **Where care is provided and changes to the healthcare environment** | |  |  |  |
| **Subcategory**  **Reference** | | | **Teams** | | **Shared decision-making** | | | **Communication between providers** | | **Care pathways** | | | **Packages of care** | | | **Health information systems** | | **The use of information and communication technology** | | | | **Role expansion or task shifting** | | | **Triage** | | **Outreach services** | |  |  |  |
| Maguire M, *et al.* 2020. | | | ✓ | |  | | |  | | ✓ | | | ✓ | | |  | |  | | | |  | | |  | |  | | 3 |  |  |
| Marwood J, *et al.* 2017. | | | ✓ | | ✓ | | | ✓ | |  | | | ✓ | | |  | | ✓ | | | |  | | |  | | ✓ | | 6 |  |  |
| Mitchell AB, *et al.* 2021. | | |  | |  | | |  | | ✓ | | | ✓ | | | ✓ | |  | | | |  | | | ✓ | | ✓ | | 5 |  |  |
| Morjaria S, *et al.* 2021. | | | ✓ | | ✓ | | | ✓ | | ✓ | | | ✓ | | | ✓ | |  | | | |  | | | ✓ | | ✓ | | 8 |  |  |
| Murphy K *et al* 2015. | | | ✓ | | ✓ | | | ✓ | | ✓ | | | ✓ | | |  | |  | | | |  | | |  | | ✓ | | 6 |  |  |
| Nguyen CT, *et al.* 2019. | | |  | |  | | |  | |  | | | ✓ | | |  | |  | | | |  | | |  | |  | | 1 |  |  |
| Parker N, *et al.*. 2018. | | |  | |  | | |  | |  | | |  | | |  | |  | | | | ✓ | | |  | |  | | 1 |  |  |
| Patel R, *et al.* 2019. | | | ✓ | |  | | |  | |  | | | ✓ | | |  | |  | | | |  | | |  | |  | | 2 |  |  |
| Phung M, *et al.* 2021. | | |  | | ✓ | | |  | |  | | | ✓ | | |  | |  | | | |  | | |  | |  | | 2 |  |  |
| Rahbani P, Monroe-Duprey L. 2020. | | |  | | ✓ | | |  | | ✓ | | | ✓ | | | ✓ | |  | | | |  | | |  | |  | | 4 |  |  |
| Rahbani P. 2019. | | |  | |  | | |  | |  | | |  | | |  | |  | | | |  | | |  | |  | | 0 |  |  |
| Ravindran S, *et al.* 2017. | | |  | |  | | |  | |  | | | ✓ | | |  | |  | | | |  | | |  | |  | | 1 |  |  |
| Rimawi RH, *et al.* 2013. | | |  | |  | | |  | | ✓ | | | ✓ | | | ✓ | |  | | | |  | | |  | | ✓ | | 4 |  |  |
| Rimawi RH, Mazer MA. 2014. | | | ✓ | |  | | |  | |  | | | ✓ | | |  | |  | | | |  | | |  | |  | | 2 |  |  |
| Sacco KA, *et al.* 2019. | | |  | |  | | |  | | ✓ | | | ✓ | | |  | |  | | | |  | | |  | |  | | 2 |  |  |
| Savic L, *et al.* 2019. | | | ✓ | | ✓ | | | ✓ | | ✓ | | | ✓ | | |  | |  | | | |  | | | ✓ | | ✓ | | 7 |  |  |
| Shannon KT, Krop LC. 2016. | | | ✓ | |  | | |  | |  | | | ✓ | | |  | |  | | | |  | | |  | |  | | 2 |  |  |
| Sigona NS, *et al.*2016. | | |  | | ✓ | | |  | | ✓ | | | ✓ | | | ✓ | |  | | | |  | | |  | | ✓ | | 5 |  |  |
| Skibba N, Fischer J, Loecker B. | | |  | |  | | |  | |  | | | ✓ | | |  | |  | | | |  | | |  | | ✓ | | 2 |  |  |
| Smibert O, *et al.* 2018. | | | ✓ | |  | | |  | |  | | |  | | |  | |  | | | |  | | |  | | ✓ | | 2 |  |  |
| Sneddon J, *et al.* 2021. | | |  | | ✓ | | |  | | ✓ | | | ✓ | | |  | |  | | | |  | | |  | |  | | 3 |  |  |
| Song Y-C, *et al.* 2021. | | |  | | ✓ | | |  | | ✓ | | | ✓ | | | ✓ | | ✓ | | | |  | | |  | |  | | 5 |  |  |
| Steenvoorden L *et al.* 2021. | | |  | | ✓ | | |  | |  | | | ✓ | | | ✓ | |  | | | |  | | |  | |  | | 3 |  |  |
| Stein A, *et al.* 2020. | | |  | |  | | |  | | ✓ | | | ✓ | | |  | |  | | | |  | | |  | |  | | 2 |  |  |
| Stone CA, *et al.*2020. | | | ✓ | | ✓ | | |  | | ✓ | | | ✓ | | |  | |  | | | |  | | |  | |  | | 4 |  |  |
| Torney N, Tiberg M. 2018. | | | ✓ | |  | | |  | |  | | | ✓ | | |  | |  | | | |  | | |  | |  | | 2 |  |  |
| Torney N, Tiberg M. 2021. | | |  | |  | | |  | | ✓ | | | ✓ | | |  | |  | | | |  | | |  | |  | | 2 |  |  |
| Trubiano JA, *et al.*2018 | | | ✓ | | ✓ | | |  | | ✓ | | | ✓ | | |  | |  | | | |  | | |  | |  | | 4 |  |  |
| Trubiano JA, *et al.* 2017. | | | ✓ | |  | | |  | |  | | | ✓ | | |  | |  | | | |  | | |  | |  | | 2 |  |  |
| Trubiano J, *et al.* 2022. | | |  | |  | | |  | |  | | | ✓ | | |  | |  | | | |  | | |  | | ✓ | | 2 |  |  |
| Vyles D, *et al.*2020. | | |  | | ✓ | | |  | |  | | | ✓ | | |  | |  | | | |  | | |  | |  | | 2 |  |  |
| Wall GC, *et al.* 2004. | | | ✓ | | ✓ | | |  | |  | | | ✓ | | |  | |  | | | |  | | |  | |  | | 3 |  |  |
| Wong J, *et al.* 2018. | | |  | |  | | | ✓ | | ✓ | | | ✓ | | |  | |  | | | |  | | |  | |  | | 3 |  |  |
| Wrenn R, *et al.*2017. | | |  | |  | | |  | |  | | |  | | |  | |  | | | |  | | |  | |  | | 0 |  |  |
| Taremi M, *et al.* 2019. | | | ✓ | | ✓ | | | ✓ | | ✓ | | | ✓ | | |  | | ✓ | | | |  | | |  | | ✓ | | 7 |  |  |
| Total | | | 32 | | 34 | | | 12 | | 44 | | | 58 | | | 17 | | 6 | | | | 2 | | | 6 | | 31 | |  |  |  |
| **Domain** | **Finance arrangements** | | | | | **Governance arrangements** | | | | | **Implementation Strategies** | | | | | | | | | | | |  | | | | | |  |  |  |
| **Category** | **Targeted financial incentives (health professionals and healthcare organisations)** | | | | | **Authority and accountability for health professionals** | | | | | **Interventions targeted at healthcare workers** | | | | | | | | | | | |  | | | | | |  |  |  |
| **Subcategory**  **Reference** | **Pay for performance / target payments** | | | | | **Authority and accountability for quality of practice** | | | | | **Continuous quality improvement** | | | **Reminders** | | | | | **Educational meetings** | **Educational materials** | | | **Other** | | | | | | Total |  |  |
| Adkinson NF *et al.* 1971. |  | | | | |  | | | | |  | | |  | | | | | ✓ |  | | |  | | | | | | 1 |  |  |
| Allen HI, *et al.* 2020. |  | | | | |  | | | | |  | | |  | | | | |  |  | | | Expert available | | | | | | 0 |  |  |
| Bauer ME, *et a*l. 2021. | ✓ | | | | | ✓ | | | | | ✓ | | |  | | | | | ✓ |  | | | Patient education materials | | | | | | 4 |  |  |
| Blackwell W, Khan D. 2020. |  | | | | |  | | | | |  | | |  | | | | | ✓ |  | | |  | | | | | | 1 |  |  |
| Blumenthal KG *et al.* 2019. |  | | | | |  | | | | |  | | | ✓ | | | | |  |  | | | Expert available | | | | | | 1 |  |  |
| Blumenthal KG *et al.* 2015. |  | | | | |  | | | | |  | | |  | | | | | ✓ | ✓ | | |  | | | | | | 2 |  |  |
| Chen JR *et al.*2017. |  | | | | | ✓ | | | | |  | | | ✓ | | | | | ✓ |  | | | Expert available | | | | | | 3 |  |  |
| Chen JR *et al.* 2018. |  | | | | |  | | | | |  | | | ✓ | | | | | ✓ |  | | | Expert available | | | | | | 2 |  |  |
| Chua KYL *et al.* 2020. |  | | | | |  | | | | |  | | |  | | | | | ✓ |  | | |  | | | | | | 1 |  |  |
| Devch, M. *et al.* 2019. |  | | | | |  | | | | |  | | |  | | | | |  |  | | |  | | | | | | 0 |  |  |
| du Plessis T, *et al.*2019. |  | | | | |  | | | | |  | | |  | | | | | ✓ |  | | | Patient education and patient material | | | | | | 1 |  |  |
| Eischens MR, *et al.* |  | | | | |  | | | | |  | | |  | | | | |  |  | | |  | | | | | | 0 |  |  |
| Englert E, Weeks A. 2019. |  | | | | | ✓ | | | | |  | | |  | | | | | ✓ |  | | | Patient education and patient material | | | | | | 2 |  |  |
| Foolad F, *et al.* 2019. |  | | | | |  | | | | |  | | |  | | | | |  |  | | | Patient information | | | | | | 0 |  |  |
| Gaudreau S, *et al*. 2021. |  | | | | |  | | | | |  | | | ✓ | | | | | ✓ |  | | |  | | | | | | 2 |  |  |
| Griffith NC *et al.* 2020. |  | | | | |  | | | | |  | | |  | | | | | ✓ |  | | | Patient education and patient material | | | | | | 1 |  |  |
| Gugkaeva Z. *et al.* 2017 |  | | | | | ✓ | | | | |  | | |  | | | | | ✓ |  | | |  | | | | | | 2 |  |  |
| Ham Y, *et al.* 2021. |  | | | | |  | | | | |  | | |  | | | | | ✓ |  | | | Access to allergy expert, patient education | | | | | | 1 |  |  |
| Harmon S, *et al.* 2020 |  | | | | | ✓ | | | | |  | | | ✓ | | | | | ✓ |  | | |  | | | | | | 3 |  |  |
| Harper HM, Sanchez M. 2021. |  | | | | |  | | | | |  | | |  | | | | | ✓ |  | | | Patient education | | | | | | 1 |  |  |
| Harris AD *et al.* 1999. |  | | | | |  | | | | |  | | |  | | | | |  |  | | |  | | | | | | 1 |  |  |
| Heil EL, *et al.* 2016. |  | | | | |  | | | | |  | | |  | | | | | ✓ |  | | |  | | | | | | 0 |  |  |
| Jones BM, 2019. |  | | | | |  | | | | |  | | |  | | | | |  |  | | | Patient education and patient material | | | | | | 1 |  |  |
| Jones BM, Bland, MC. 2017. |  | | | | |  | | | | |  | | |  | | | | | ✓ |  | | | Patient education | | | | | | 1 |  |  |
| Jones BM, *et al.* 2019. |  | | | | |  | | | | |  | | |  | | | | | ✓ |  | | |  | | | | | | 1 |  |  |
| Kleris R, *et al.*2018. |  | | | | |  | | | | |  | | |  | | | | |  |  | | |  | | | | | | 0 |  |  |
| Kyi L, *et al.* 2018. |  | | | | |  | | | | |  | | |  | | | | |  |  | | |  | | | | | | 0 |  |  |
| Lecerf K, *et al*. 2020. |  | | | | |  | | | | |  | | | ✓ | | | | |  |  | | |  | | | | | | 1 |  |  |
| Leis *et al.* 2017. |  | | | | |  | | | | |  | | |  | | | | | ✓ |  | | | Patient education material | | | | | | 1 |  |  |
| Lin L *et al.* 2020 |  | | | | |  | | | | |  | | | ✓ | | | | | ✓ | ✓ | | |  | | | | | | 3 |  |  |
| Livirya S, et al. 2020. |  | | | | |  | | | | |  | | |  | | | | |  |  | | | Patient education material | | | | | | 0 |  |  |
| Lnumerables F, Fischer-Cartlidge E. |  | | | | |  | | | | |  | | |  | | | | |  |  | | |  | | | | | | 0 |  |  |
| Lo SCR *et al* 2020. |  | | | | |  | | | | |  | | |  | | | | | ✓ |  | | |  | | | | | | 1 |  |  |
| Louden NJ, *et al.* 2021. |  | | | | |  | | | | | ✓ | | |  | | | | | ✓ |  | | |  | | | | | | 2 |  |  |
| Maguire M, *et al.* 2020. |  | | | | |  | | | | |  | | |  | | | | | ✓ |  | | |  | | | | | | 1 |  |  |
| Marwood J, *et al.* 2017. |  | | | | |  | | | | |  | | |  | | | | | ✓ |  | | | Patient material | | | | | | 1 |  |  |
| Mitchell AB, *et al.* 2021. |  | | | | |  | | | | |  | | |  | | | | | ✓ |  | | | Access to allergy expert | | | | | | 1 |  |  |
| Morjaria S, *et al.* 2021. |  | | | | |  | | | | |  | | |  | | | | | ✓ |  | | | Patient material, bedside hypersensitivity kit | | | | | | 1 |  |  |
| Murphy K *et al* 2015. |  | | | | |  | | | | |  | | |  | | | | |  |  | | | Patient material | | | | | | 0 |  |  |
| Nguyen CT, *et al.* 2019. |  | | | | |  | | | | |  | | |  | | | | |  |  | | |  | | | | | | 0 |  |  |
| Parker N, *et al.*. 2018. |  | | | | |  | | | | |  | | |  | | | | |  |  | | |  | | | | | | 0 |  |  |
| Patel R, *et al.* 2019. |  | | | | |  | | | | |  | | | ✓ | | | | |  |  | | |  | | | | | | 1 |  |  |
| Phung M, *et al.* 2021. |  | | | | |  | | | | |  | | |  | | | | |  |  | | |  | | | | | | 0 |  |  |
| Rahbani P, Monroe-Duprey L. 2020. |  | | | | |  | | | | |  | | |  | | | | |  |  | | |  | | | | | | 0 |  |  |
| Rahbani P. 2019. |  | | | | |  | | | | |  | | |  | | | | |  | ✓ | | |  | | | | | | 1 |  |  |
| Ravindran S, *et al.* 2017. |  | | | | |  | | | | |  | | |  | | | | |  |  | | |  | | | | | | 0 |  |  |
| Rimawi RH, *et al.* 2013. |  | | | | | ✓ | | | | |  | | |  | | | | |  |  | | |  | | | | | | 1 |  |  |
| Rimawi RH, Mazer MA. 2014. |  | | | | |  | | | | |  | | |  | | | | | ✓ |  | | |  | | | | | | 1 |  |  |
| Sacco KA, *et al.* 2019. |  | | | | | ✓ | | | | |  | | |  | | | | | ✓ |  | | |  | | | | | | 2 |  |  |
| Savic L, *et al.* 2019. |  | | | | |  | | | | |  | | |  | | | | |  |  | | | Resus equipment and personnel available | | | | | | 0 |  |  |
| Shannon KT, Krop LC. 2016. |  | | | | |  | | | | |  | | |  | | | | | ✓ |  | | | Access to allergy experts (pharmacist) | | | | | | 1 |  |  |
| Sigona NS, *et al.*2016. |  | | | | |  | | | | |  | | |  | | | | | ✓ |  | | |  | | | | | | 1 |  |  |
| Skibba N, Fischer J, Loecker B. |  | | | | |  | | | | |  | | |  | | | | |  |  | | |  | | | | | | 0 |  |  |
| Smibert O, *et al.* 2018. |  | | | | |  | | | | |  | | |  | | | | |  |  | | |  | | | | | | 0 |  |  |
| Sneddon J, *et al.* 2021. |  | | | | | ✓ | | | | |  | | |  | | | | |  | ✓ | | | Patient material | | | | | | 2 |  |  |
| Song Y-C, *et al.* 2021. |  | | | | | ✓ | | | | |  | | |  | | | | |  |  | | | Patient material | | | | | | 1 |  |  |
| Steenvoorden L *et al.* 2021. |  | | | | |  | | | | |  | | |  | | | | |  |  | | | Emergency kit with adrenalin available | | | | | | 0 |  |  |
| Stein A, *et al.* 2020. |  | | | | |  | | | | |  | | |  | | | | |  |  | | |  | | | | | | 0 |  |  |
| Stone CA, *et al.*2020. |  | | | | |  | | | | |  | | |  | | | | |  |  | | |  | | | | | | 0 |  |  |
| Torney N, Tiberg M. 2018. |  | | | | |  | | | | |  | | |  | | | | | ✓ |  | | |  | | | | | | 1 |  |  |
| Torney N, Tiberg M. 2021. |  | | | | | ✓ | | | | |  | | |  | | | | | ✓ |  | | | Access to allergy expert | | | | | | 2 |  |  |
| Trubiano JA, *et al.*2018^,^ |  | | | | |  | | | | |  | | |  | | | | |  |  | | |  | | | | | | 0 |  |  |
| Trubiano JA, *et al.* 2017. |  | | | | |  | | | | |  | | |  | | | | |  |  | | |  | | | | | | 0 |  |  |
| Trubiano J, *et al.* 2022. |  | | | | |  | | | | |  | | |  | | | | | ✓ |  | | |  | | | | | | 1 |  |  |
| Vyles D, *et al.*2020. |  | | | | |  | | | | |  | | |  | | | | |  |  | | |  | | | | | | 0 |  |  |
| Wall GC, *et al.* 2004. |  | | | | | ✓ | | | | |  | | |  | | | | | ✓ |  | | |  | | | | | | 2 |  |  |
| Wong J, *et al.* 2018. |  | | | | |  | | | | |  | | |  | | | | |  |  | | |  | | | | | | 0 |  |  |
| Wrenn R, *et al.*2017. |  | | | | |  | | | | |  | | |  | | | | | ✓ |  | | | Access to expert | | | | | | 1 |  |  |
| Taremi M, *et al.* 2019. |  | | | | | ✓ | | | | |  | | |  | | | | | ✓ |  | | |  | | | | | | 2 |  |  |
| Total | 1 | | | | | 12 | | | | | 2 | | | 8 | | | | | 36 | 4 | | | Expert 9; patient education/material 17; access to resus equipment/personal 3 | | | | | |  |  |  |

**Appendix 9. Meta-analysis forest plots**


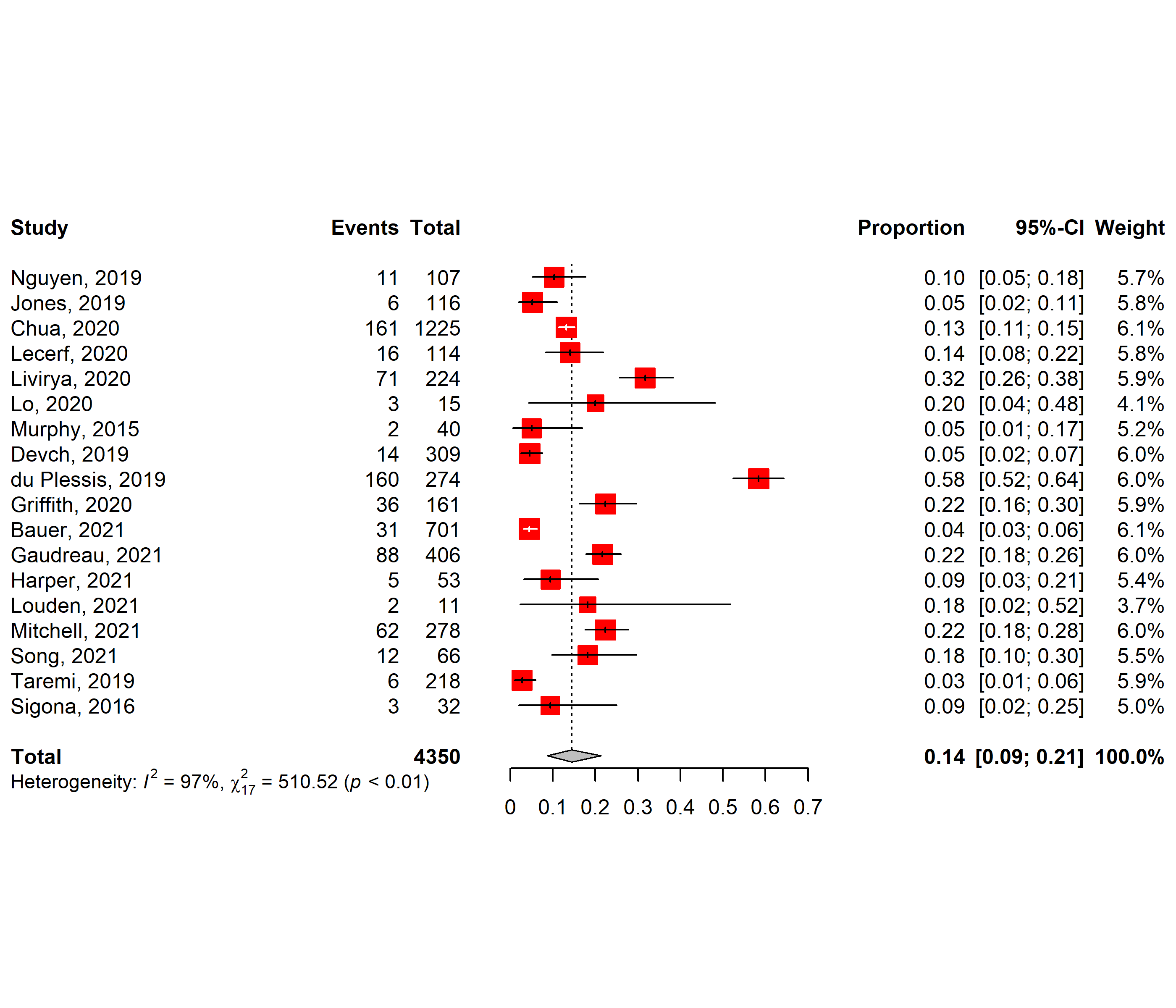


Figure A1. Forest plot showing the proportion of assessed patients successfully de-labelled by direct de-label (DDL)


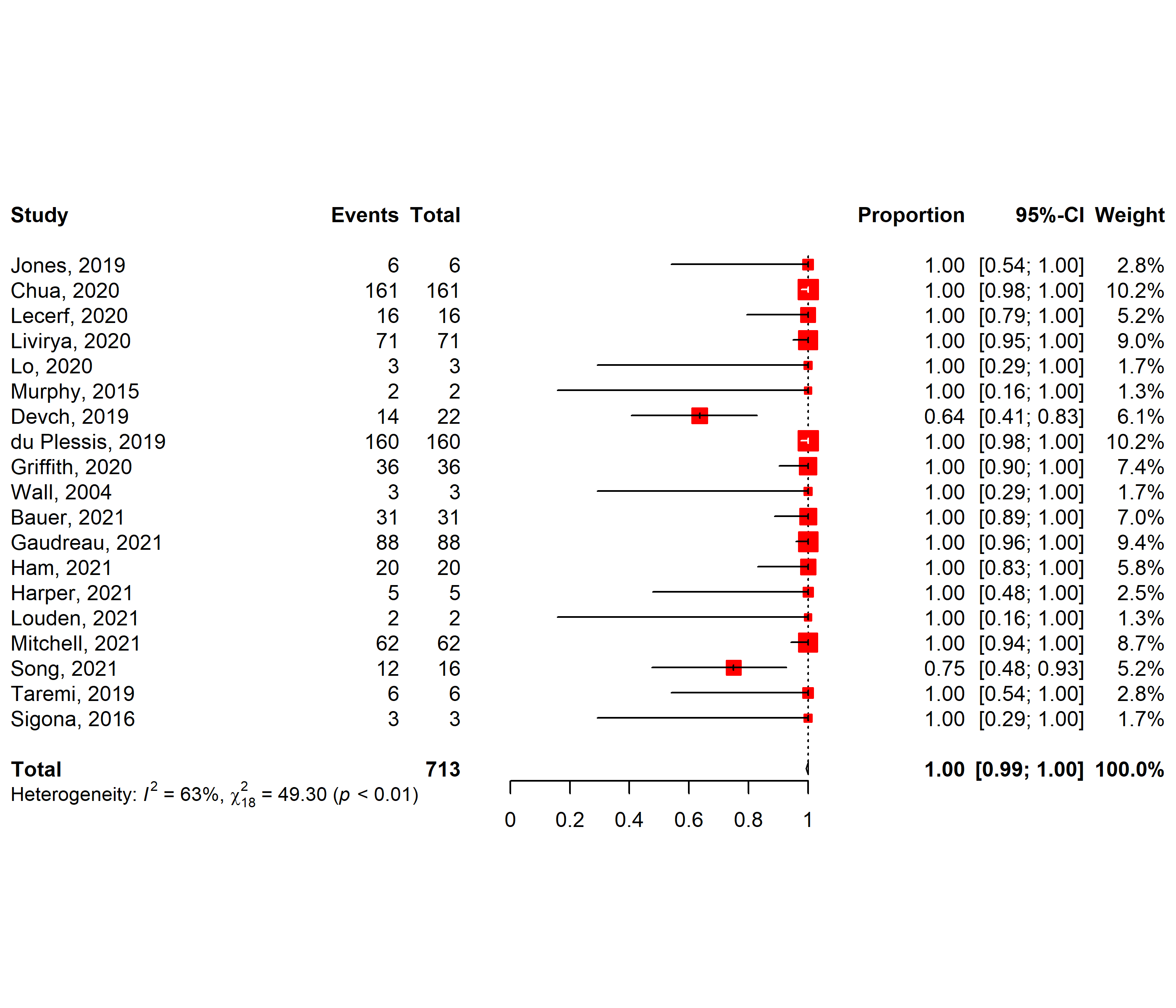


Figure A2. Forest plot showing the proportion of patients identified for DDL that were successfully de-labeled on history alone.


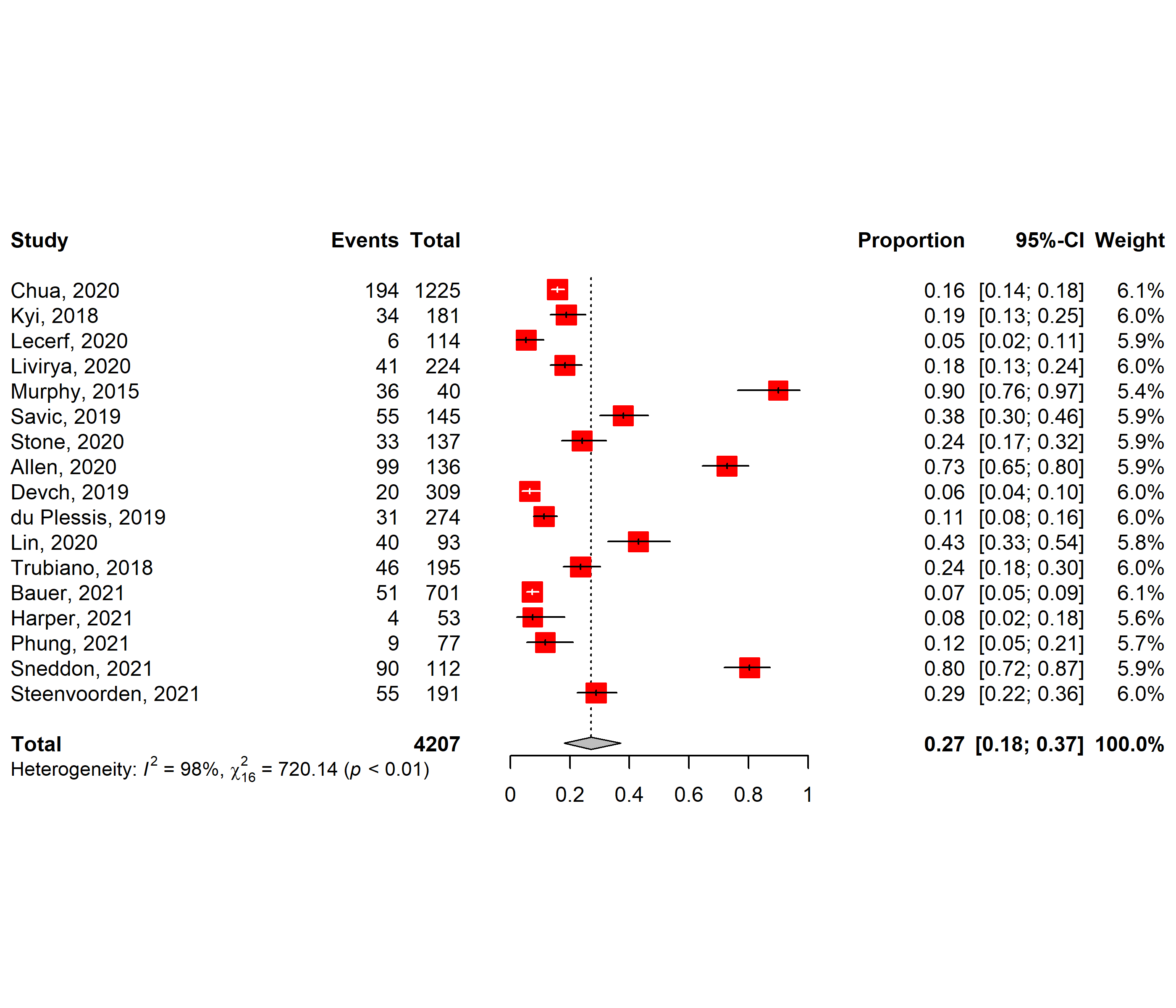


Figure A3. Forest plot showing the proportion of assessed patients successfully de-labelled by direct provocation testing (DPT).


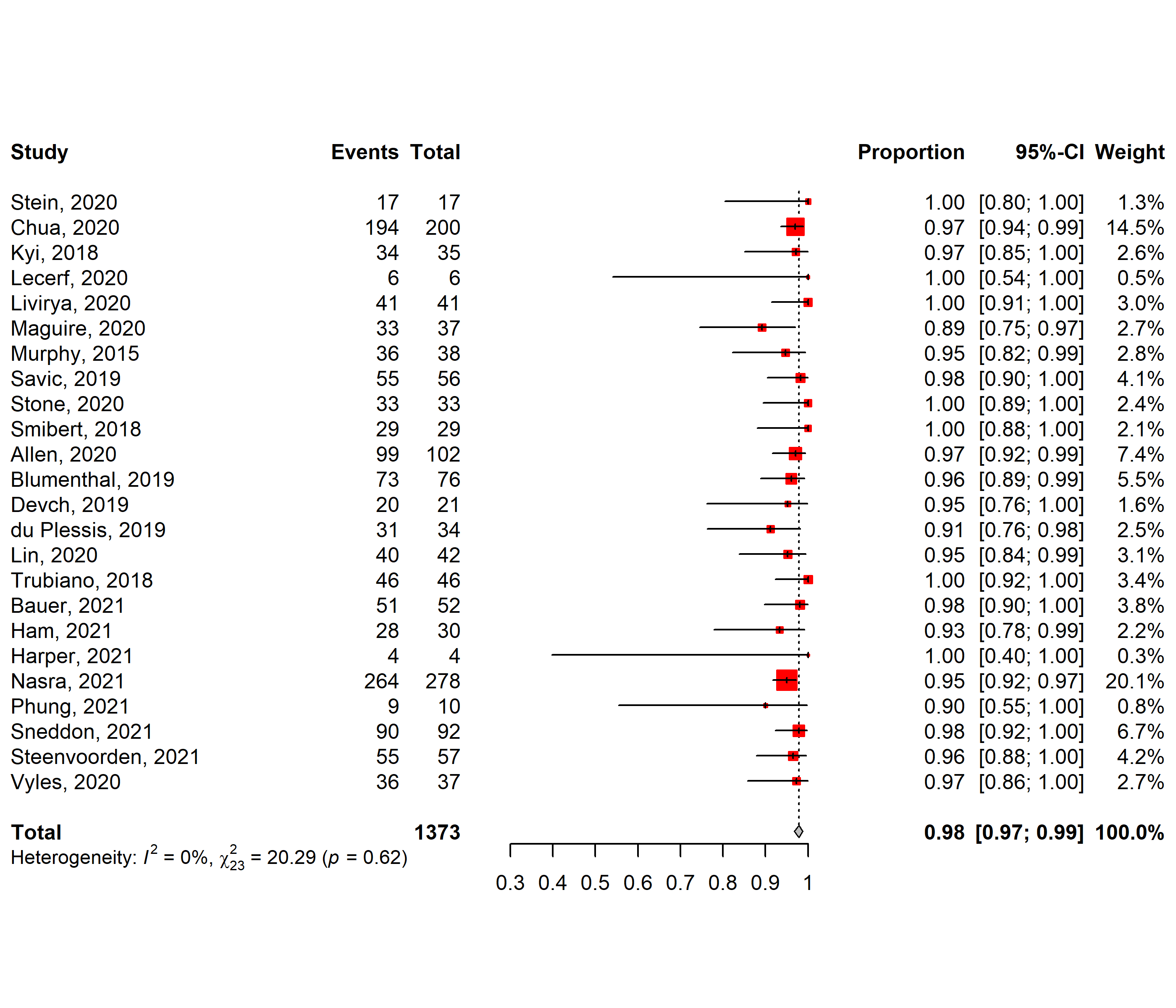


Figure A4. Forest plot showing the proportion of patients identified for DPT that were successfully de-labeled by DPT.


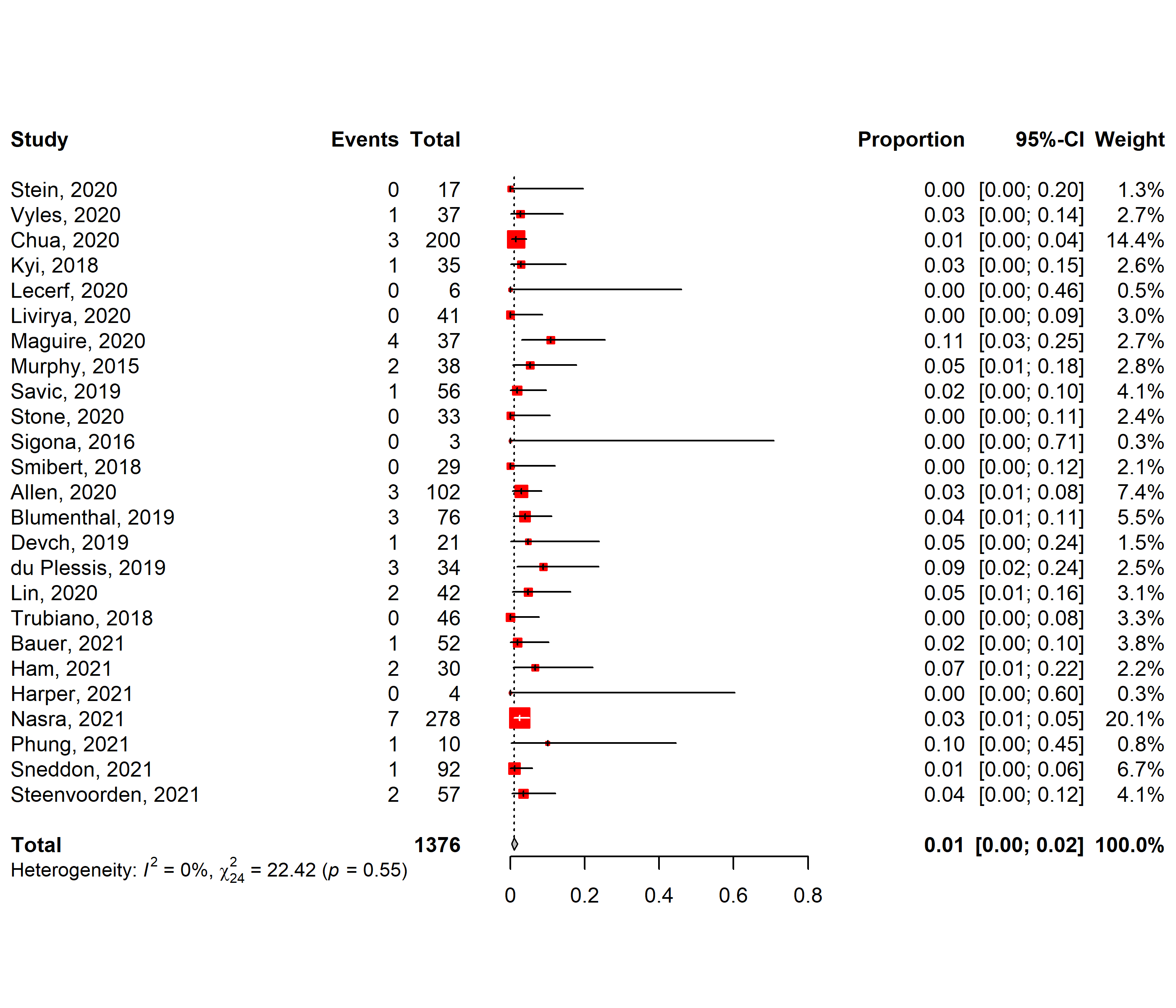


Figure A5 Forest plot showing the proportion of patients harmed via DPT


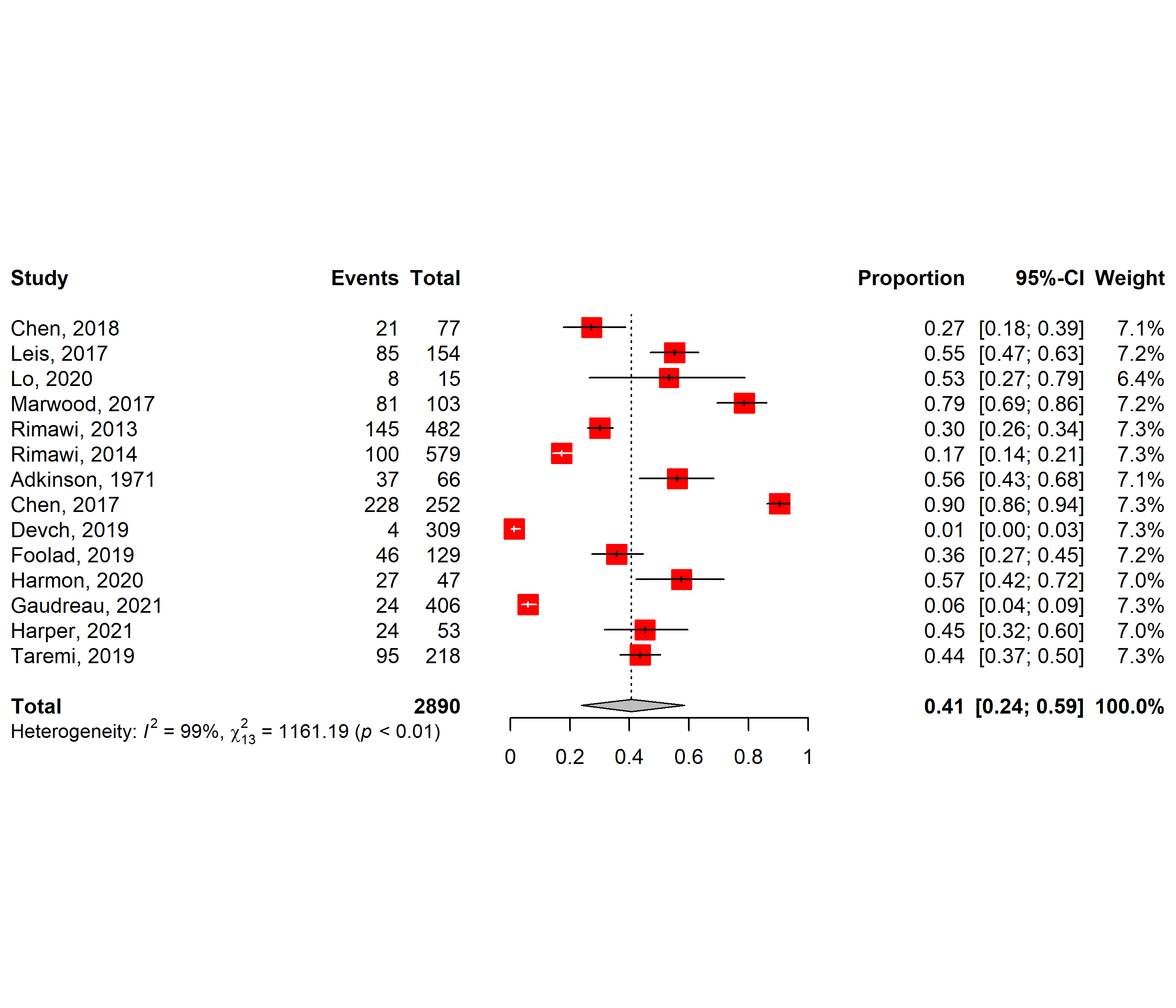


Figure A6 Forest plot showing the proportion of assessed patients successfully de-labelled by ST/OC or IVC.


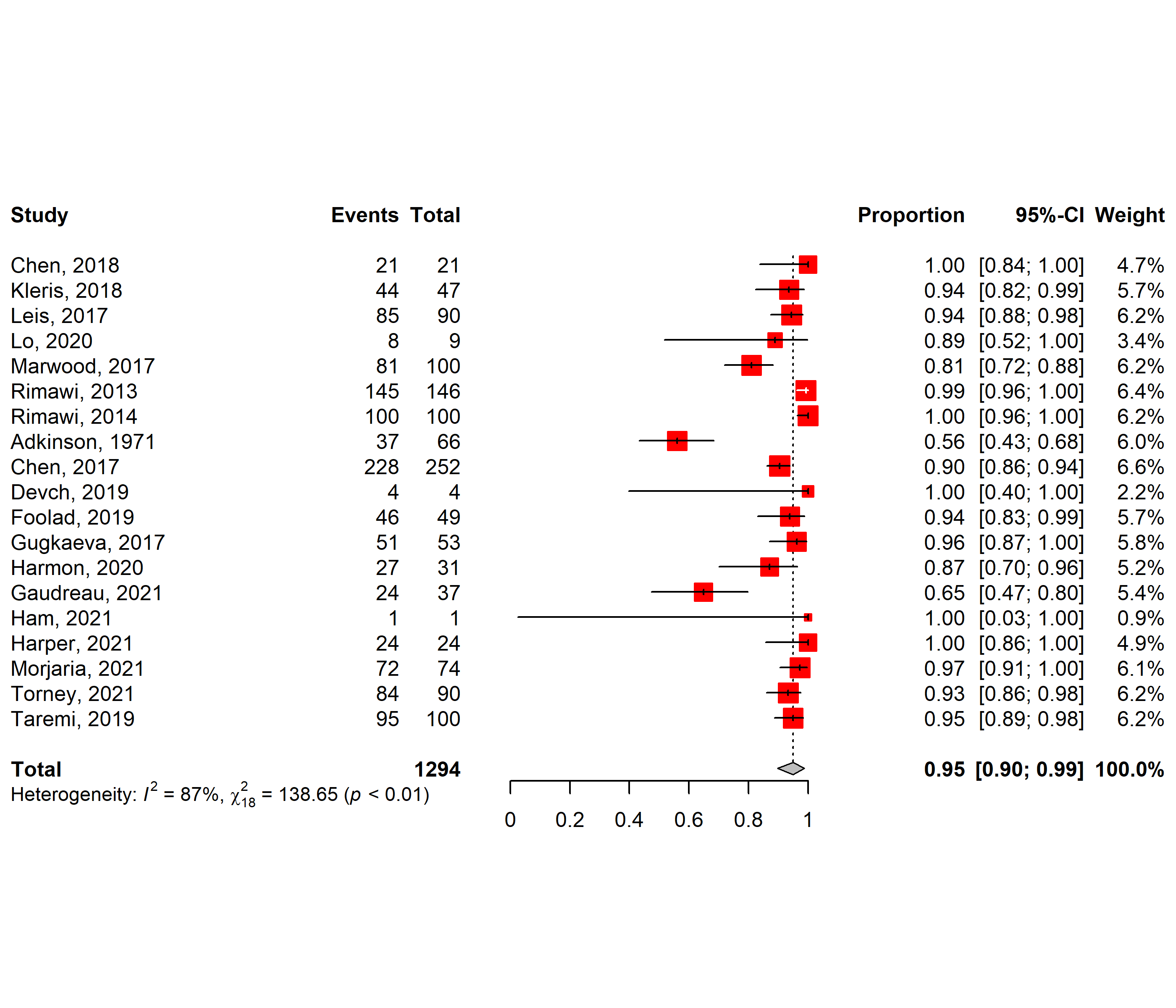


Figure A7 Forest plot showing the proportion of tested patients successfully de-labelled by ST/OC or IVC.


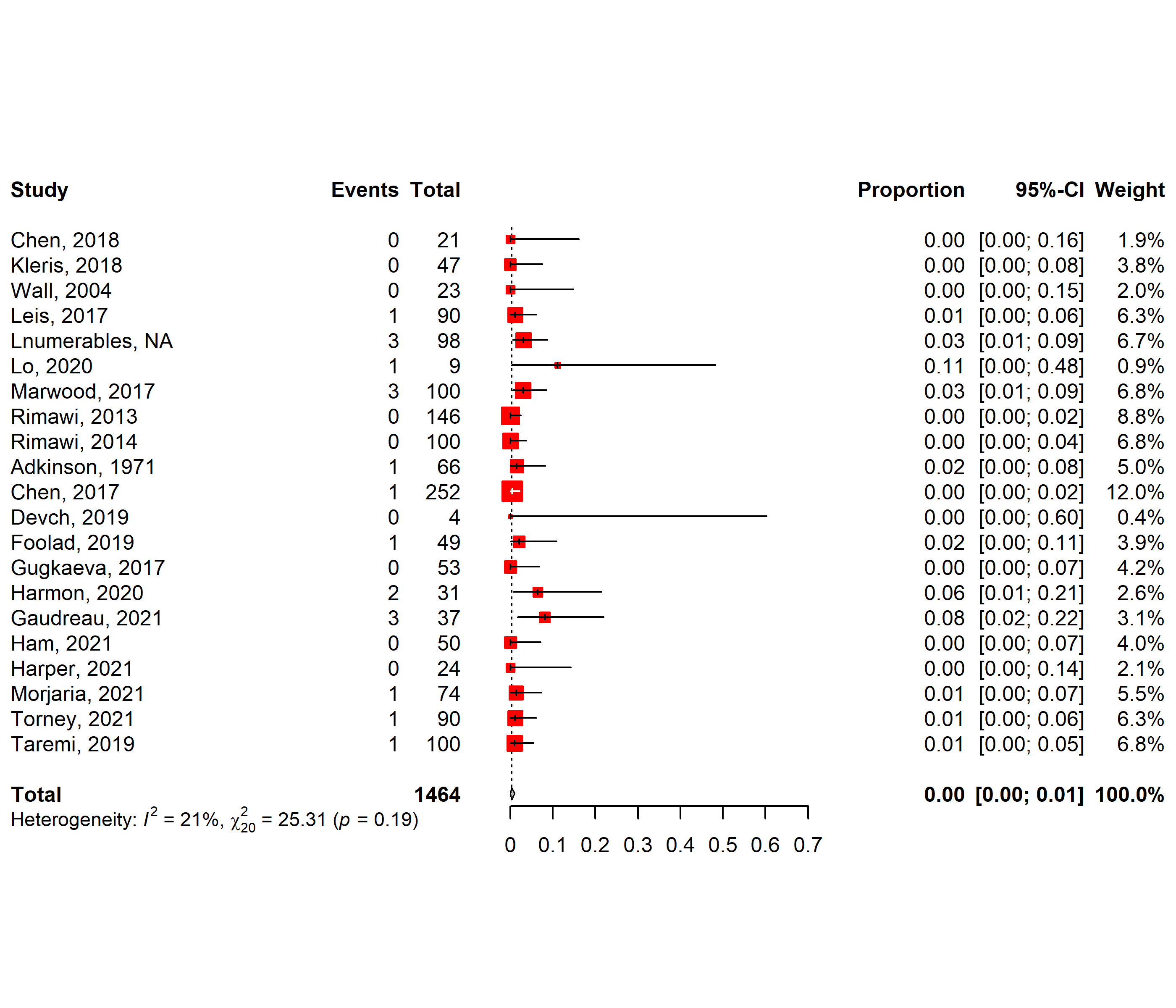


Figure A8 Forest plot showing the proportion of patients harmed via ST/OC or IVC.

**Appendix 10. Funnel plots**


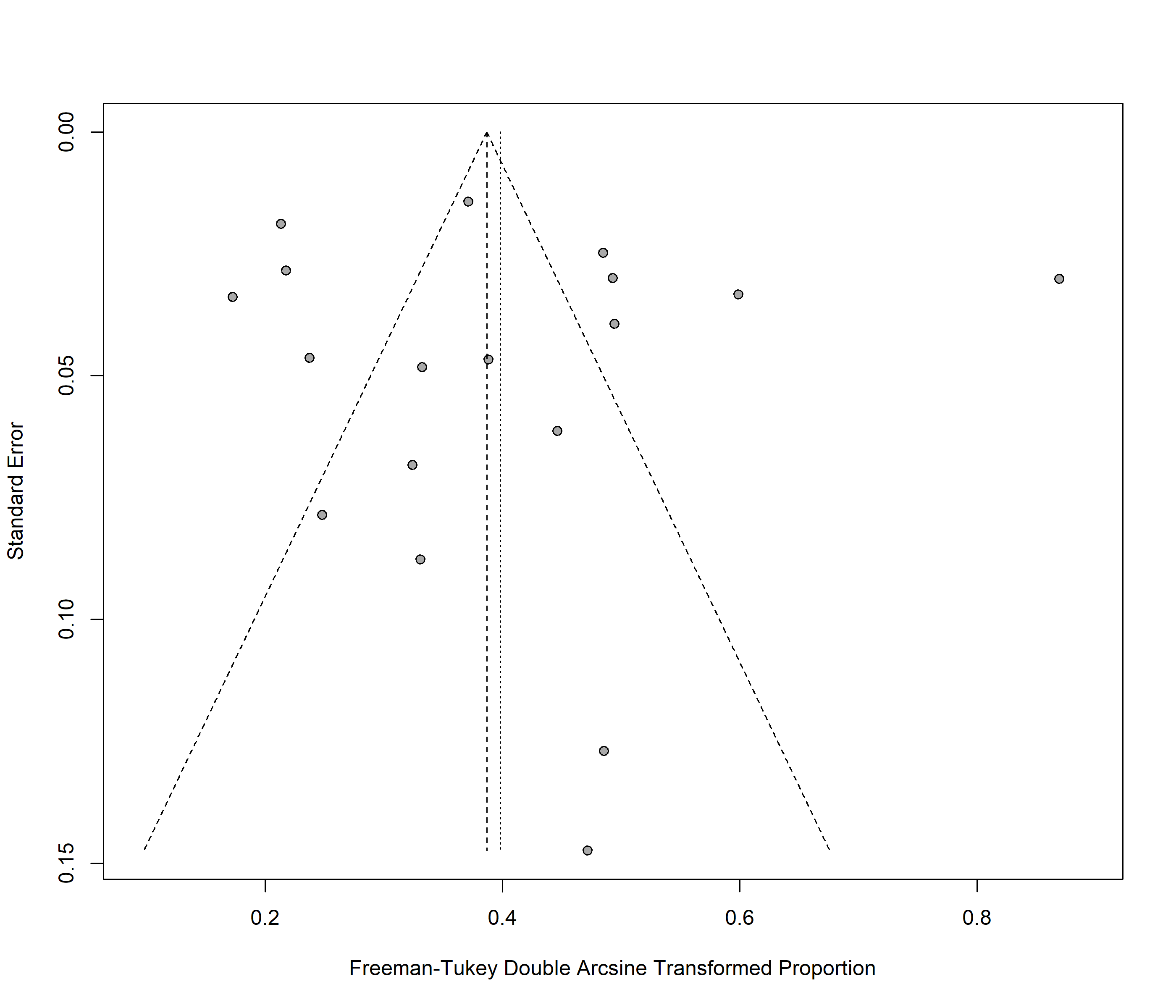

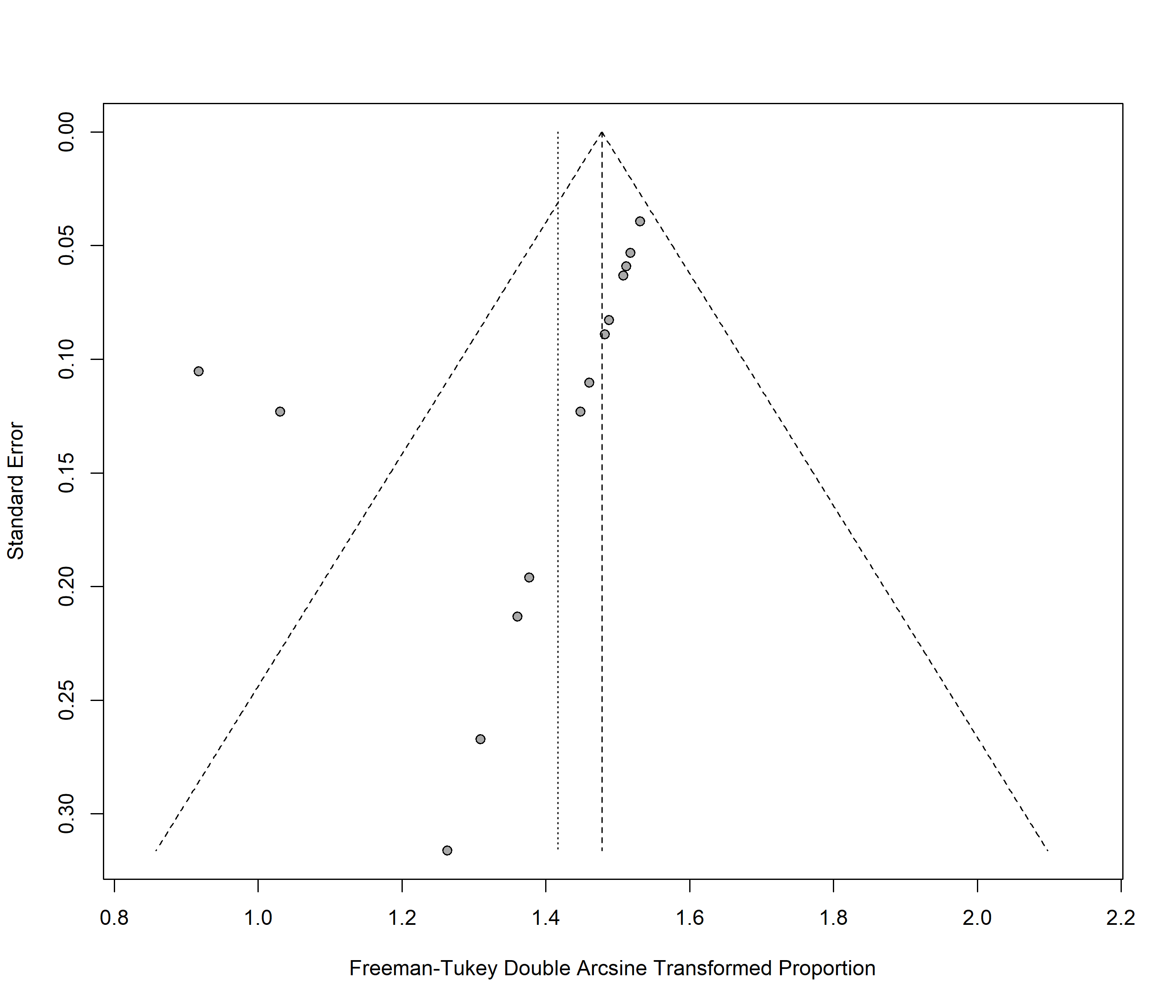


Fig. 9A. Proportion of patients assessed de-labelled by direct de-label Fig. 9B. Proportion of patients successfully de-labelled by direct de-label

Egger's test p-value 0.2084 Egger's test p-value0.0001


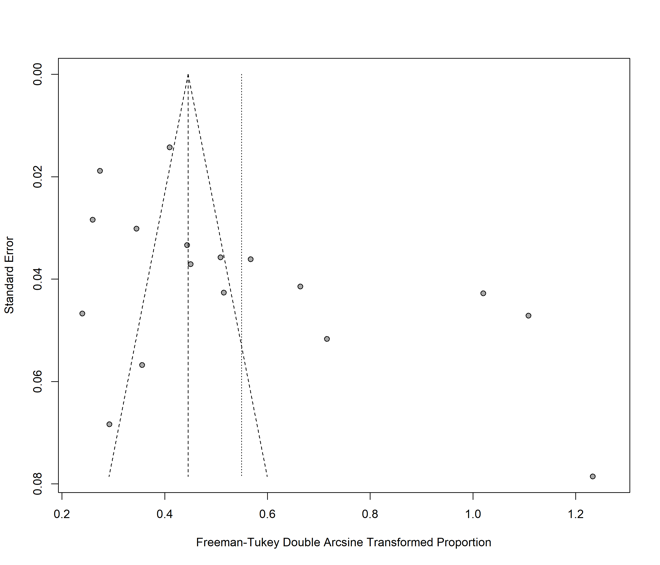

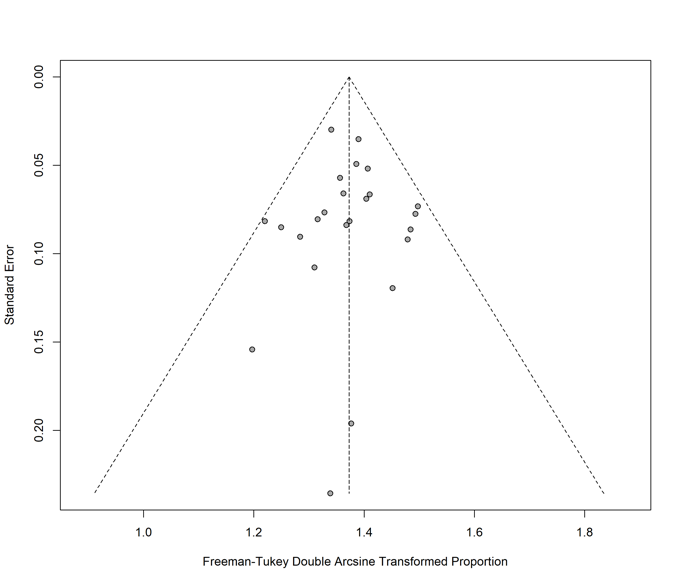

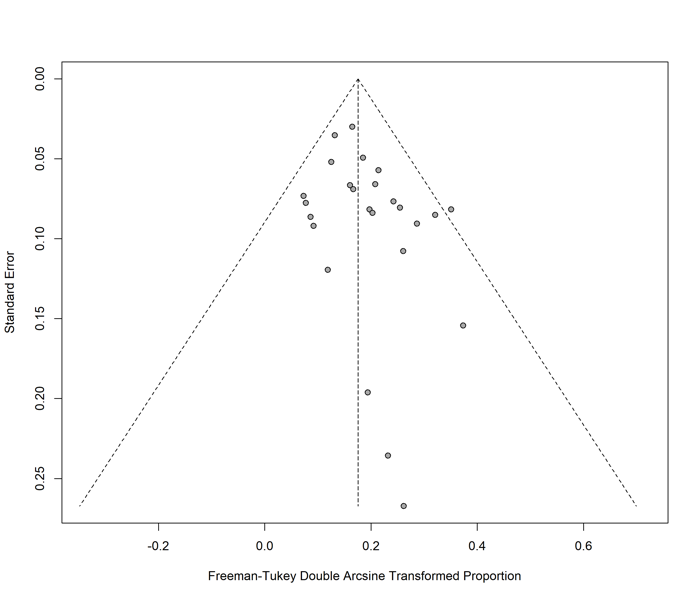


Fig 10 A. Proportion of assessed PenA de-labelled by DPT Fig 10 B. Proportion of PenA tested via DPT de-labelled by DPT Fig 10 C. Proportion of PenA de-labelled by DPT harmed

Egger's test p-value 0.3452 Egger's test p-value 0.1574 Egger's test p-value 0.1646


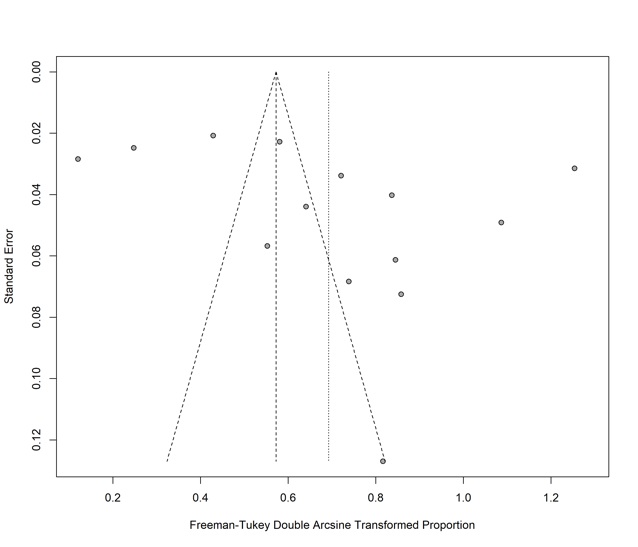

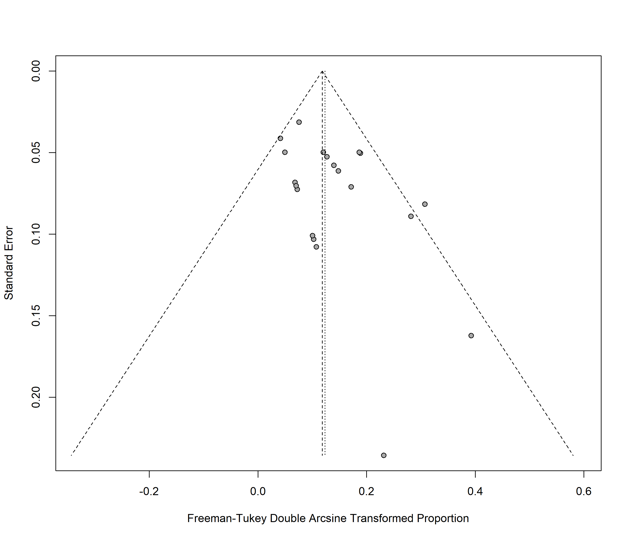

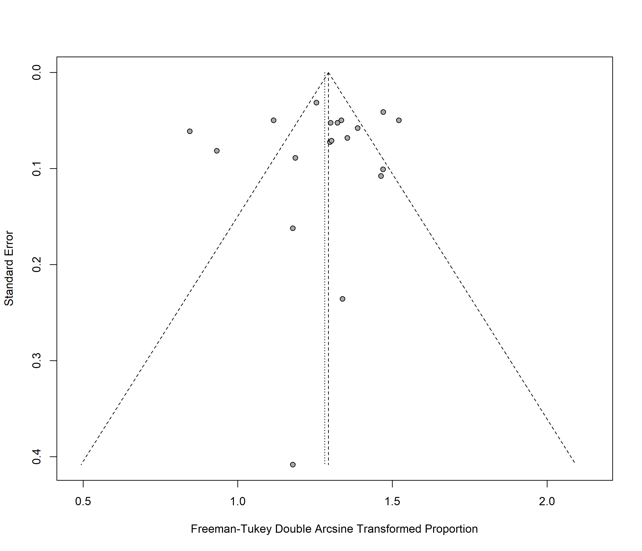


Fig. 11A. Proportion of assessed PenA de-labelled by ST/OC Fig11 B. Proportion of PenA tested via ST/OC de-labelled by ST/OC Fig11 C. Proportion of PenA de-labelled by ST/OC with harm

| **Forest plot** | **Egger's test p-value** |
| --- | --- |
| Proportion of patients assessed de-labelled by direct de-label | 0.2084 |
| Proportion of patients successfully de-labelled by direct de-label | 0.0001 |
| Proportion of assessed PenA de-labelled by DPT | 0.3452 |
| Proportion of PenA tested via DPT de-labelled | 0.1574 |
| Proportion of PenA de-labelled by DPT harmed | 0.1646 |
| Proportion of assessed PenA de-labelled by ST/OC | 0.4934 |
| Proportion of PenA tested via ST/OC de-labelled by ST/OC | 0.0199 |
| Proportion of PenA de-labelled by ST/OC with harm | 0.0166 |

Egger's test p-value 0.4934 Egger's test p-value 0.0199 Egger's test p-value 0.0166

Funnel plots

The table gives the p-values from Egger’s regression, a test for publication bias. The values < 0.05 mean we reject the null hypothesis that there was no publication bias in the associated studies.

**Appendix 11 Sensitivity analysis**


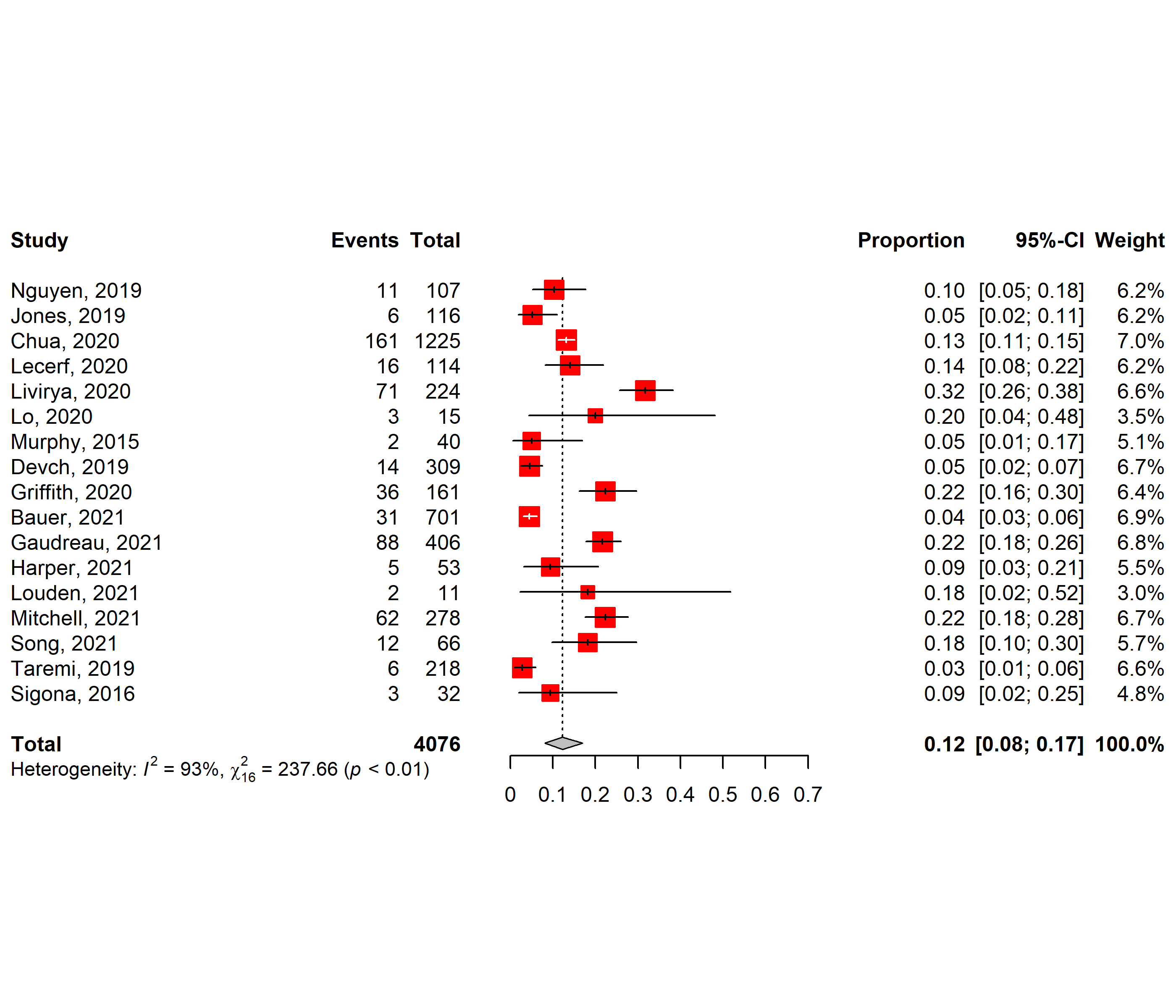
Figure 12. Forest plot showing the proportion of assessed patients successfully de-labelled by direct de-label (DDL). (du Plessis 2019 removed).


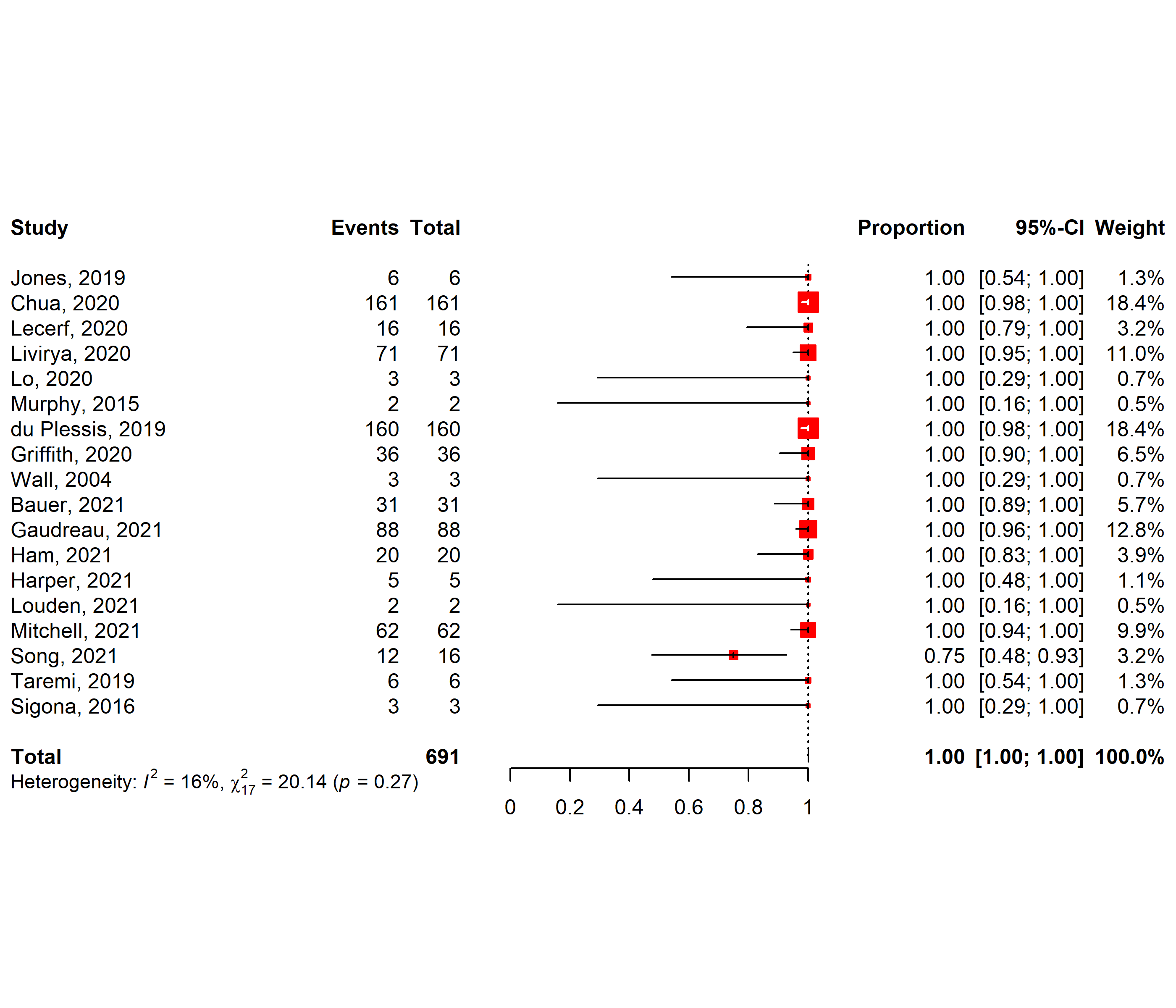
Figure 13. Forest plot showing the proportion of patients identified for DDL that were successfully de-labeled on history alone. (Devch 2019 removed).


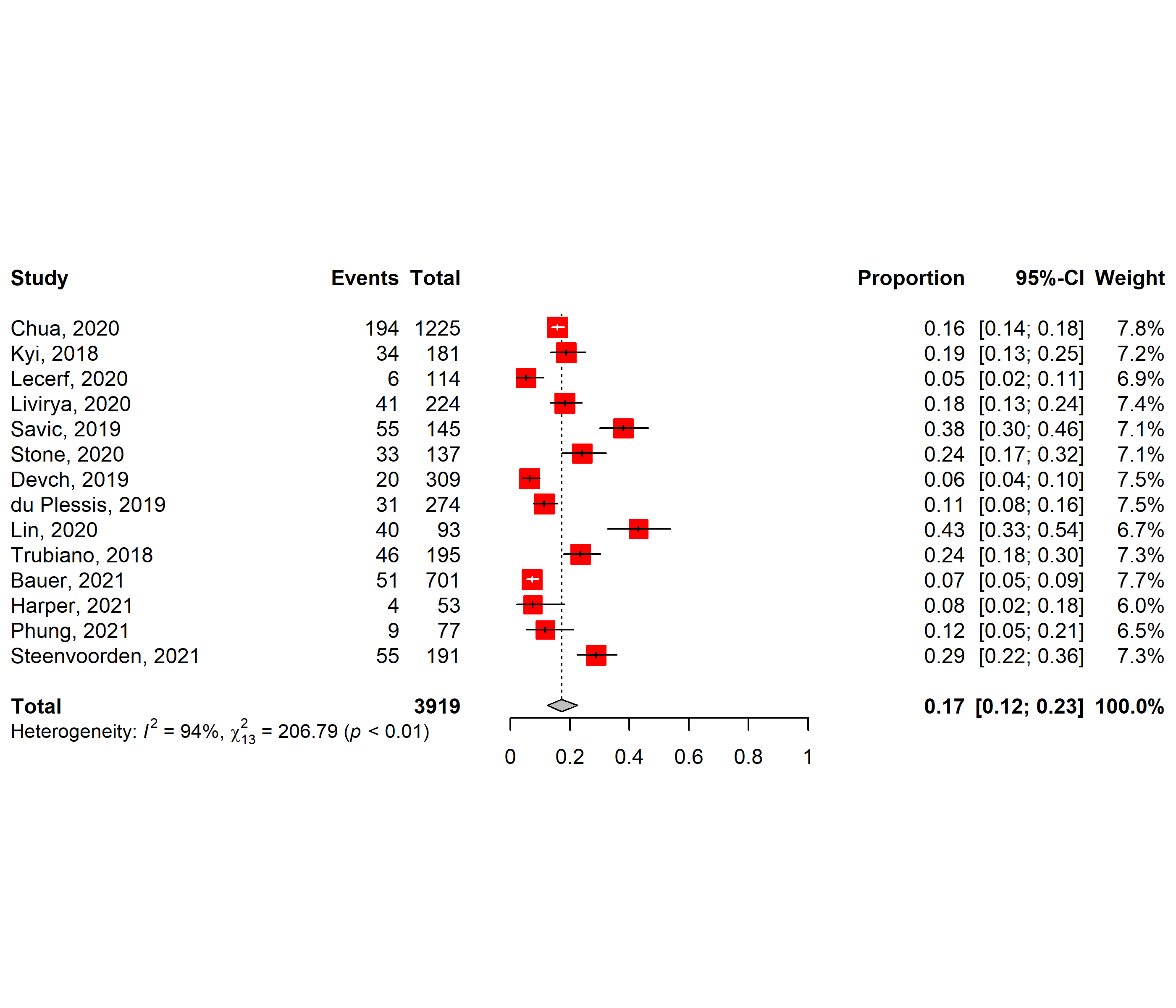
Figure 14. Forest plot showing the proportion of assessed patients successfully de-labelled by direct provocation testing (DPT). (Murphy 2015, Allen 2020, Sneddon 2021 removed)


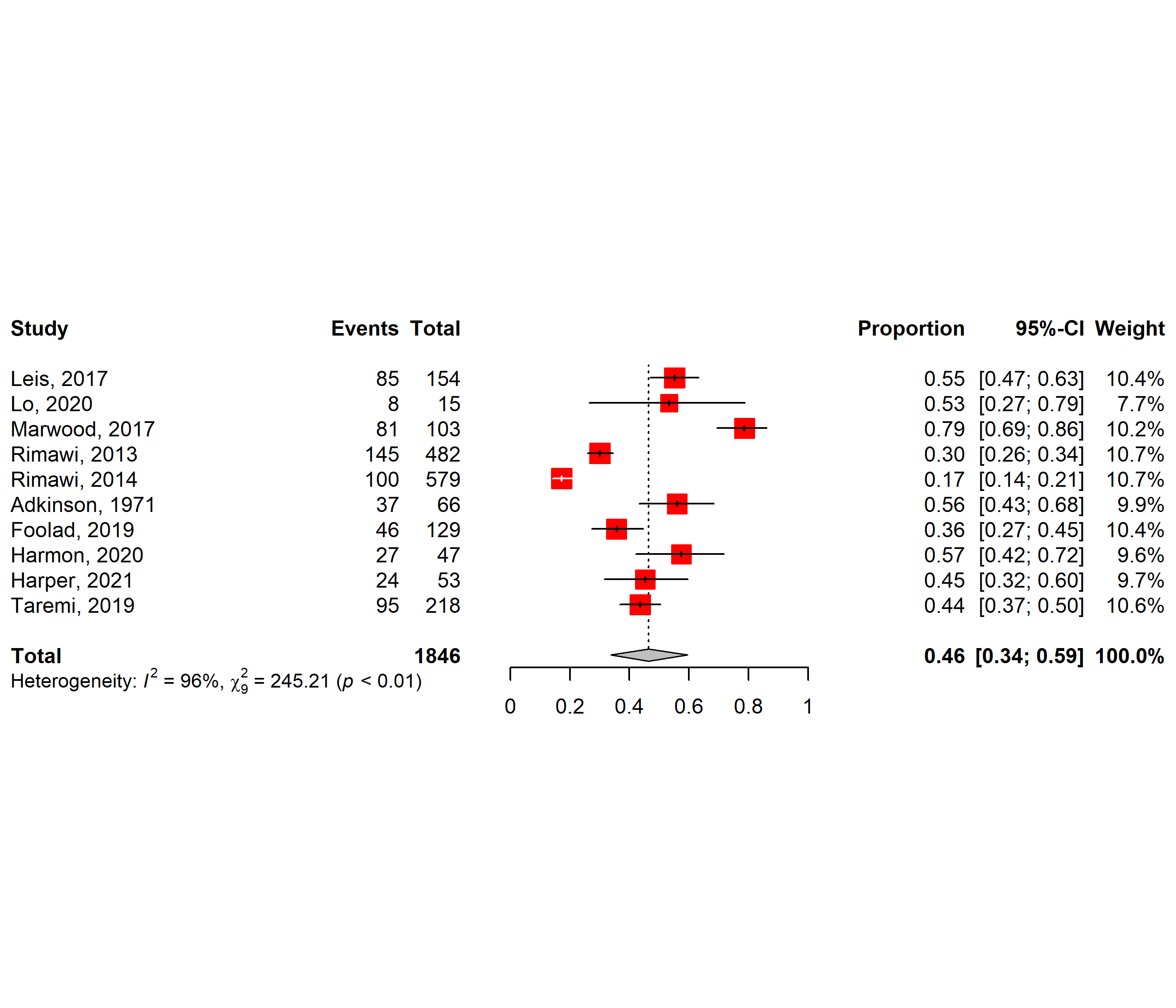


Figure 15 Forest plot showing the proportion of assessed patients successfully de-labelled by ST/OC or IVC. (Chen 2017, Devch 2019, Gaudreau 2021, removed).


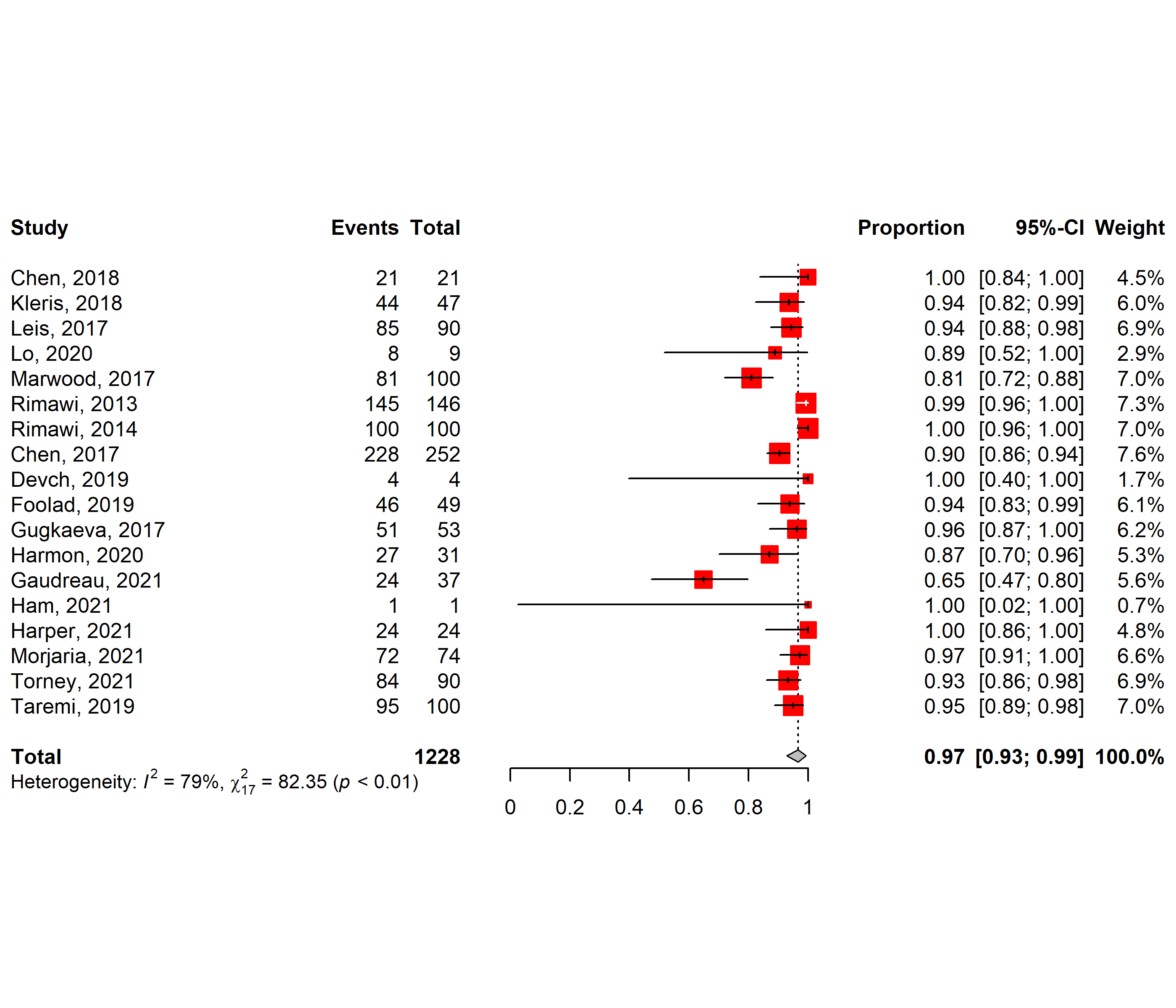


Figure 16 Forest plot showing the proportion of tested patients successfully de-labelled by ST/OC or IVC. (Atkinson 1971 removed)

**Appendix 12 extraction check**

Twenty-six data points were checked by a second reviewer. The data points with identified errors are shown in the table below. There were no errors in extraction for the following data points in any of the 7 studies where data was extracted by a second author: "Study type", "abstract, brief report, or full paper", "Country, State, City", "Setting / context", "age", "gender", "Hospital specialties / wards", "targeted all antibiotics, all beta-lactams, penicillin only", "exclusion criteria", "low risk patients, moderate risk patients, high risk patients", "The penA de-label method used ", The interventions to enable the de-labelling", The number of patients with a penicillin allergy record successfully de-labelled", "proportion of patients de-labelled (tested)", "Any measured antimicrobial stewardship impact", "Any measured healthcare system impact", "Any measured healthcare system impact", "Any unintended harm associated with the de-label process", " number of patients experiencing unintended harm ", "Proportion experiencing unintended harm associated with the penA de-label process". Combined, there were 7 errors across 182 data points (3.8%) identified.

| **Reference** | **adult or paeds** | **inclusion criteria** | **Healthcare workers involved in the de-label process** | **The number of adults with a penicillin allergy record successfully de-labelled** | **Number of patients in study (screened)** | **number of pen A patients penA tested** | **proportion of patients de-labelled (screened)** | **correct number of extraction data points** | **total data points** |
| --- | --- | --- | --- | --- | --- | --- | --- | --- | --- |
| Blumenthal KG, Shenoy ES, Varughese C, Hurwitz S, Hooper D, Banerji A. 2015.^84^ |  |  |  | should be 36, not 26 as originally stated | should say "not stated" | should be 36, not 26 as originally stated |  | 23 | 26 |
| Eischens MR, Wolf LM, Dumkow LE, Anderson AM, Jameson AP, Br, et al.^28^ |  |  | should read "Pharmacists plus others (not stated)" instead of ED Pharmacists & ED providers" |  |  |  |  | 25 | 26 |
| Nguyen CT, Sahbani O, Pisano J, Pursell K, Pettit NN. 2019.^81^ |  |  |  |  |  |  |  | 26 | 26 |
| Rahbani P. 2019.^38^ | should be "not reported" instead of adults | should say "not reported" instead of "penicillin allergy" |  |  |  |  |  | 24 | 26 |
| Steenvoorden L, Bjoernestad EO, Kvesetmoen T-A, Gulsvik AK. 2021.^76^ |  |  |  |  |  |  |  | 26 | 26 |
| Torney NP, Tiberg MD. 2021.^79^ |  |  |  |  |  |  |  | 26 | 26 |
| Vyles D, Chiu A, Routes J, Castells M, Phillips EJ, Visotcky A, et al. 2020.^95^ |  |  |  |  |  |  | should say "indeterminable" instead of "21.8% of screened (but 50% were randomised to be tested and half not tested)" | 25 | 26 |
